# Intracellular delivery and deep tissue penetration of nucleoside triphosphates using photocleavable covalently bound dendritic polycations†

**DOI:** 10.1039/d3sc05669d

**Published:** 2024-04-02

**Authors:** Jiahui Ma, Johanna Wehrle, Dennis Frank, Lina Lorenzen, Christoph Popp, Wolfgang Driever, Robert Grosse, Henning J. Jessen

**Affiliations:** a Institute of Organic Chemistry, Faculty of Chemistry and Pharmacy, University of Freiburg Albertstr. 21 79104 Freiburg Germany henning.jessen@oc.uni-freiburg.de; b CIBSS—Centre for Integrative Biological Signaling Studies, University of Freiburg 79104 Freiburg Germany; c Faculty of Biology, University of Freiburg Hauptstr. 1 79104 Freiburg Germany; d Institute of Experimental and Clinical Pharmacology and Toxicology, Medical Faculty, University of Freiburg Albertstr. 25 79104 Freiburg Germany

## Abstract

Nucleoside triphosphates (NTPs) are essential in various biological processes. Cellular or even organismal controlled delivery of NTPs would be highly desirable, yet *in cellulo* and *in vivo* applications are hampered owing to their negative charge leading to cell impermeability. NTP transporters or NTP prodrugs have been developed, but a spatial and temporal control of the release of the investigated molecules remains challenging with these strategies. Herein, we describe a general approach to enable intracellular delivery of NTPs using covalently bound dendritic polycations, which are derived from PAMAM dendrons and their guanidinium derivatives. By design, these modifications are fully removable through attachment on a photocage, ready to deliver the native NTP upon irradiation enabling spatiotemporal control over nucleotide release. We study the intracellular distribution of the compounds depending on the linker and dendron generation as well as side chain modifications. Importantly, as the polycation is bound covalently, these molecules can also penetrate deeply into the tissue of living organisms, such as zebrafish.

## Abstract 2

Polycationic dendrons covalently linked to photocaged nucleoside triphosphates enable cellular delivery and deep tissue penetration.

## Graphical abstract

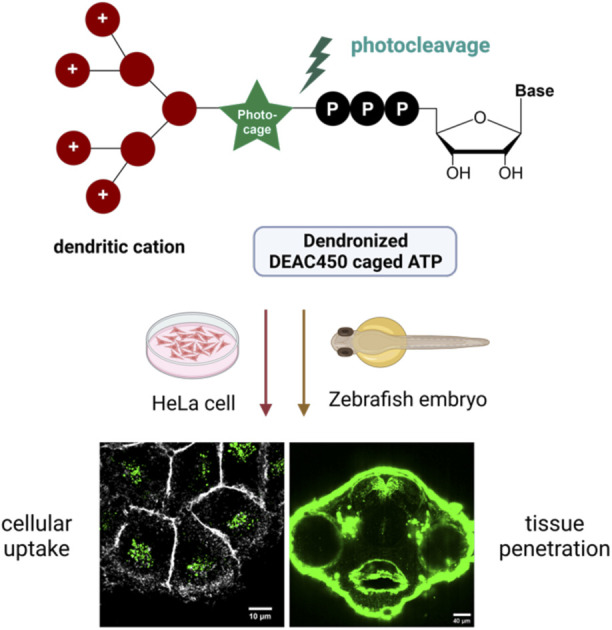

Nucleoside triphosphates (NTPs) play a fundamental role in most biological processes. They are not only essential precursors for DNA and RNA synthesis, but also provide energy for cellular reactions. In addition, they are involved in a number of cellular functions, for instance, signaling and transport,^1^ and are also essential in the synthesis of glycogen, lipids, and cofactors.^2^ Unnatural NTP analogues have important applications in anticancer and antiviral chemotherapy.^3–8^ Usually, the nucleoside has to be phosphorylated in cells to the biologically active triphosphate. To avoid anabolic bottlenecks, also NMP, NDP, and more recently NTP prodrugs have been introduced.^3,4,9–13^ Common prodrug approaches can result in limited uptake efficiency into tissues, as the phosphate masking groups are designed to be quickly removed in cells, which hampers deep tissue penetration. Likewise, clinically used aryl amidate prodrugs, such as sofosbuvir, tenofovir alafenamide, and remdesivir are also based on enzymatic hydrolysis, in which the activation of the prodrugs are not potentially subject to an extracellular trigger.^14^

Recent developments rely on lipophilic modifications of γ-phosphates of NTP analogues, which facilitate uptake and release the active molecules by enzymatic cleavage at physiological pH.^3,4^ Polymer-based drug carriers containing positive charges are also capable of loading and delivering molecules into cells *via* formation of non-covalent complexes.^5–7^ In another study, a polyamine linker was introduced to an ATP-biotin covalently, resulting in a cell-permeable ATP analogue, however in this design, release of the native NTP cargo is not possible.^15^ A more general NTP transporter was established based on a combination of using a per-6-amino-β-cyclodextrin as a receptor, which forms complexes with NTPs and an arginine-rich molecular transporter as a cell-penetrating agent.^16^ These efforts have greatly contributed to the study of NTPs and their analogues inside cells.

Nucleotide delivery is enhanced by removal of negative charge and/or by addition of net positive charge to the molecule.^15^ Positively charged delivery systems can rely on, for example, polyamidoamine (PAMAM) dendrons and guanidinium-rich molecular transporters (GRTs). Both approaches are extensively investigated for molecule delivery.^17–19^ The surface primary amines of dendrons and guanidine groups from GRTs are partially protonated at physiological pH,^19–22^ providing positive charges. Of note, lower generation PAMAM dendrons are less cytotoxic and more biocompatible than those of higher generations.^23,24^ In an alternative design, oligonucleotide delivery has also been described relying on thiol-mediated exchange, representing a novel delivery paradigm.^25^

To enable a more precise control over molecule delivery and release, one strategy is to use photocages. Photocages are a great boon to study functions of biomolecules by first rendering them biologically inert and subsequently activating them by irradiation. Using light as an external trigger allows us to manipulate a certain biological process induced by the active molecule spatially, temporally, and in precise dosage.^26,27^ Among the numerous known photocages, those based on a coumarin scaffold have several advantages for their use in living systems, such as single cells or even whole organisms. For instance, coumarins have a high biocompatibility,^28–31^ relatively high uncaging efficiency,^27,32,33^ flexibility of structural modifications with tunable photophysical and photochemical properties^27,34^ and a well-studied mechanism of photocleavage.^35–37^ A broad variety of functional groups have been released from their coumarin-caged precursors including phosphates.^38–45^

Importantly, Ellis-Davies has previously shown that the coumarin scaffold, specifically 7-diethylaminocoumarin 450 (DEAC450), can be functionalized with PEG-dendrons using click chemistry. This modification led to largely reduced interactions of the caged molecule (γ-amino butyric acid, GABA) with its receptor prior to uncaging due to steric crowding.^46^ They also applied the same strategy to introduce other larger dendrons derived from 2,2-bis(methylol)propionic acid. This further reduced the antagonistic effects.^47^ Also the group of Nadler has designed related clickable cages and applied them to lipids. This particular study was also addressing the subcellular targeting of the lipid, which is an important new aspect of photocage design.^48^

Based on these promising results, we envisioned to use DEAC450 with polycationic modifications to achieve organismal delivery of NTPs. In this approach, dendronized DEAC450 would be responsible for (sub)cellular delivery of the cargo and, due to the covalent binding of the delivery/transport vehicle, might enable deep tissue penetration of the probe. After delivery, one could remove the combined cage and delivery substructure in a traceless way by photouncaging. We chose ATP as a model compound for our studies, but it is important to keep in mind that a wide variety of natural and unnatural nucleoside oligophosphates could be delivered and released using our general “transporter cage” design.

The following Fig. 1 gives an overview of the design and synthesis of transporter caged ATP: a red-shifted clickable photocage installed on the γ-phosphate of ATP using P-amidite chemistry^49,50^ is subsequently modified with dendronized polyamines and polyguanidines.

**Fig. 1:**
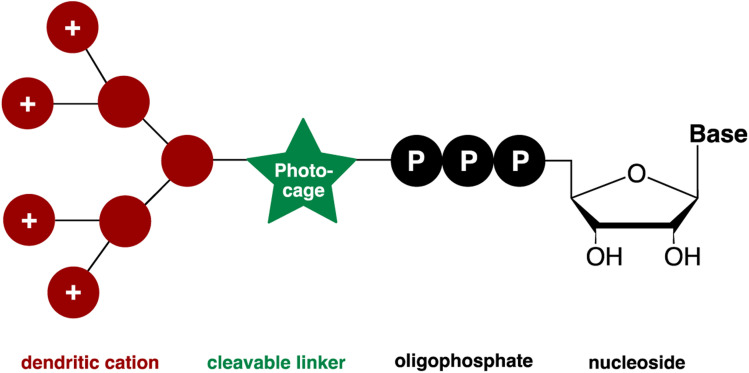
Designed molecule to enable cellular uptake and spatiotemporal control of the release of nucleotides.

The synthesis of photocage 3 (Scheme 1A) was based on DEAC450 1 modification. 1 was obtained according to a previously reported synthesis (Scheme S1†).^51^ Afterwards, an alkyne group was introduced to this photocage by peptide coupling to generate a clickable version of DEAC450 2 (Scheme 1A). Deprotection of the silyl group afforded alcohol 3, which reacted with phosphordiamidite 4, giving phosphoramidite 5 (Scheme 1B). Phosphoramidite 5 was used as a precursor for generating DEAC450 caged ATP 6 (Scheme 1B) ready for click chemistry.^52^ The synthesis strategy of coupling, oxidation and deprotection to obtain modified P-anhydrides has been previously described.^49,50,53,54^ One of its salient features is the possibility to run oligophosphate synthesis without protecting groups on the substrates, in this case ADP, and – by extension – a large variety of phosphorylated molecules.

**Scheme 1:**
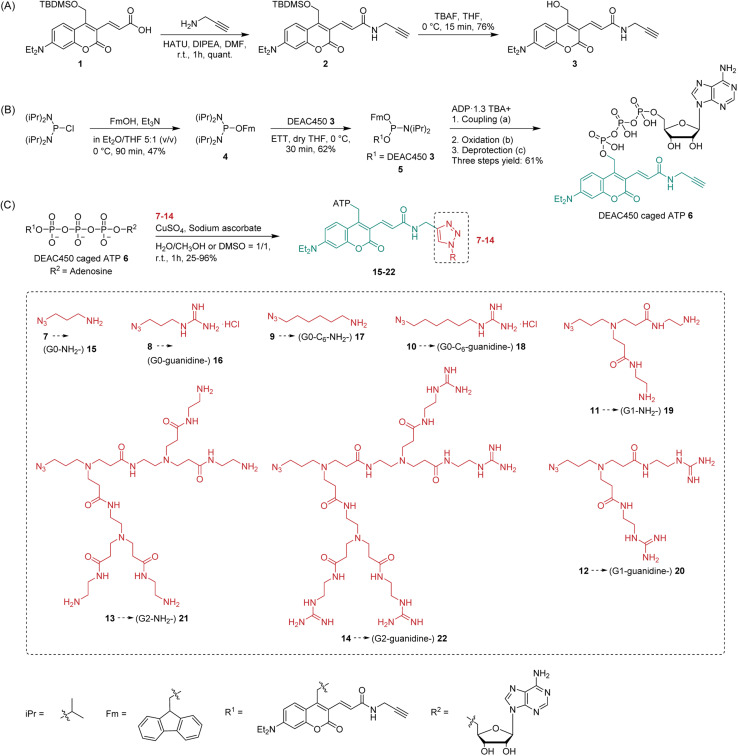
(A) Synthesis of clickable version of DEAC450 3. (B) Synthesis of DEAC450 caged ATP 6. (a) ETT, DMF, r.t., 5 min. (b) *m*CPBA, DMF, 0 °C, 5 min. (c) 5–10% (v/v) piperidine, r.t., 40 min, in DMF. The product is obtained as triethylammonium (TEA) salt after purification. (C) Synthesis of dendronized DEAC450 caged ATPs with different transporters attached and structures of dendrons used in this study.

Taking into consideration both the number of positive charges provided by dendrons and their associated cytotoxicity, we initiated our study from PAMAM generations G0–G2. Azide-functionalized PAMAM dendrons G0–G2 7, 11, 13 were synthesized according to previously reported synthetic routes *via* repetition of Michael addition and amidation starting from 7.^55^ To convert the surface amino groups of the PAMAM dendrons to guanidinium groups, 1*H*-pyrazole-1-carboxamidine hydrochloride was applied as guanidinylation reagent.^56^ It was reported that different alkyl spacers with guanidine functionalization can lead to different subcellular localizations.^57^ Thus, terminal amine 9 and guanidine 10 with a longer alkyl chain containing six carbons were synthesized from 1,6-dibromohexane. 9 was synthesized according to a previously reported procedure.^58^ Afterwards, guanidinylation of the terminal amine group was applied to obtain 10. In total, eight potential transporter modifications of various size and functionalized with different terminal groups were synthesized (Scheme 1C).

To install transporters covalently onto DEAC450 caged ATP 6, copper(i)-catalyzed azide–alkyne cycloaddition (CuAAC)^59^ was conducted (Scheme 1C). All eight potential transporters 7–14 were introduced to caged ATP 6 by click chemistry, giving molecules 15–22 in 25–96% yield. The diversity of complex structures obtained highlights the modularity of the approach (Scheme 1C).

Absorption (*λ*^max^_abs_) and emission (*λ*^max^_flu_) maxima of photocage 3 and caged molecule 6 were determined (Fig. S1†). The results were comparable, giving absorption maxima around 450 nm and emission maxima around 550 nm. Thus, compared to the DEACM photocage, a significant red-shift is achieved that is potentially beneficial for *in vivo* uncaging experiments. We next investigated uncaging of 6 in aqueous solution by performing a photolysis study with a LED setup. (Fig. S2;† Mightex® High-Power LED Collimator Sources, 22 mm aperture, 490 nm, typical output power 140 mW.) The uncaging process was analyzed by HPLC-UV, showing ATP was cleanly released upon irradiation (Fig. 2A). The uncaging kinetics were obtained from HPLC analysis (Table S1†), demonstrating almost full release of ATP within 7 minutes (Fig. 2B).

**Fig. 2:**
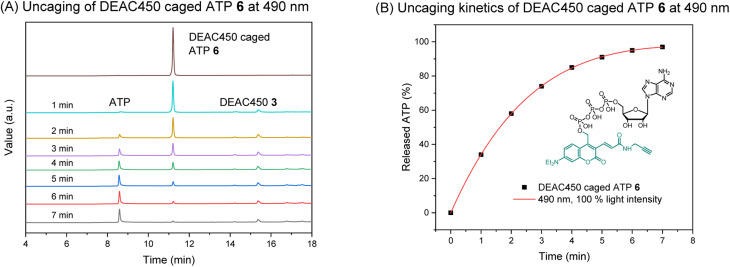
(A) HPLC analysis of the uncaging of DEAC450 caged ATP 6 at 490 nm. Concentration: 100 μM. Volume: 1 mL. (B) Uncaging kinetics of DEAC450 caged ATP 6 at 490 nm.

To evaluate the cellular uptake of molecules 15–22 (Scheme 1C), experiments were performed using HeLa cells. Fluorescence activated cell sorting (FACS) analytics were used for an initial screening of *e.g.* compounds 6, and 15–20. The FACS results are shown in the ESI (Fig. S3†). Compared to the controls (blank and compound 6), cells treated with 15–19 had significantly increased fluorescence values. The detriment of this method is that there is no information on the localization of the compounds and that compounds sticking to the cell surface would be counted as positive signals in this analysis. For this reason, we further evaluated the uptake of these compounds using fluorescence microscopy. 1 mM stock solution of compounds in DMEM was added to dishes containing HeLa cells with a final concentration of 50 μM. Cells were incubated in the dark at 37 °C for 5 h, then washed with PBS buffer and stained with CellMask™ deep red plasma membrane stain, which is compatible with the DEAC450 emission. Finally, cells were imaged with super-resolution microscopy (Elyra 7, Zeiss). Compounds 15–22 were stable for several hours under incubation conditions as judged by HPLC (Fig. S4†).

From the live imaging, cells treated with most compounds showed an enhancement in intracellular fluorescence, with the notable exception of compound 16 (Fig. 3). These results demonstrate that the combination of a photocage with an uptake-enhancing dendron is principally a successful design. Especially those molecules containing larger sized transporters — (G1–NH_2_–) 19, (G2–NH_2_–) 21, (G2–guanidine–) 22 exhibited high levels of intracellular fluorescence. Interestingly, the molecules were not evenly distributed within the cytoplasm but appeared as localized spots (Fig. 3). This may indicate energy-dependent uptake through the endosomal pathway.

**Fig. 3:**
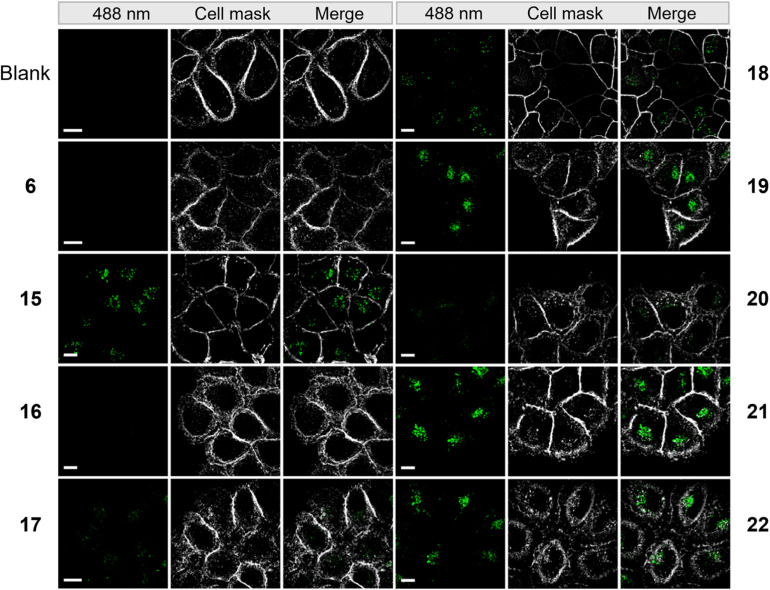
Microscopy images of HeLa cells incubated with 50 μM of compounds from a super resolution Lattice SIM microscope. Scale bar represents 10 μm.

To elucidate whether the uptake of these molecules is energy dependent, cells treated with (G1–NH_2_–) 19 were incubated at 4 °C and 37 °C, respectively, and analyzed by flow cytometry. As can be seen in the ESI (Fig. S5†), the fluorescence signal of the compounds was significantly reduced when the incubation was performed at 4 °C. This indicates that the uptake of the molecules is energy dependent and therefore not occurring by simple passive diffusion.

To elucidate the whereabouts of the caged nucleotides, we continued our study with colocalization experiments. It was previously reported that PAMAM–NH_2_ localize in the mitochondria and nuclei preferentially, due to the surface positive charges.^60^ It was also reported that guanidinium-rich molecular transporters are able to traverse the nuclear membrane and accumulate in the nucleus, leading to interesting applications in drug delivery.^61–64^ We conducted experiments to elucidate the distribution of molecules 19, 21, 22, which showed the most interesting properties. SiR-DNA was used to identify cell nuclei. HeLa cells were incubated with (G1–NH_2_–) 19 and (G2–guanidine–) 22 for 5 h. Afterwards, the cells were washed with PBS buffer. Subsequently, staining solution containing SiR-DNA was added, and then cells were imaged by Lattice SIM microscopy. This experiment showed no colocalization of 19 and 22 with the nuclear stain. Instead, we found them adjacent to the nuclear periphery, which might be a result of accumulation in the Golgi apparatus (ESI, Fig. S6†). Therefore, colocalization was conducted using BODIPY® TR ceramide to stain the Golgi. The images from Golgi colocalization (Fig. 4A) of compounds (G1–NH_2_–) 19, (G2–NH_2_–) 21, (G2–guanidine–) 22 showed that 2% of Golgi signal colocalizes with the compounds 19, 22 and 19% of Golgi signal colocalizes with the compound 21. This indicates a significant portion of 21 localizes within the Golgi apparatus, while most of 19 and 22 do not reside within the Golgi. Colocalization analysis of 21 and 22 showed changes of the distribution at different incubation times (from 0.5 h to 5 h) (Fig. 4B). The images showed that no significant amount of 21 and 22 reached the Golgi when they were incubated for 0.5 h only. The percentage of 21 that accumulates in the Golgi increased from 7% to 19%, when the incubation time was extended from 2 h to 5 h. However, no significantly increased amount of 22 localizes to Golgi, although more intense fluorescence generated from compounds was observed in Golgi area. This indicates that 21 partially targets the Golgi, while 22 does not. This reveals an important feature of the dendronized structure: one can control (to some extent) the (time-dependent) localization of the compounds.

**Fig. 4:**
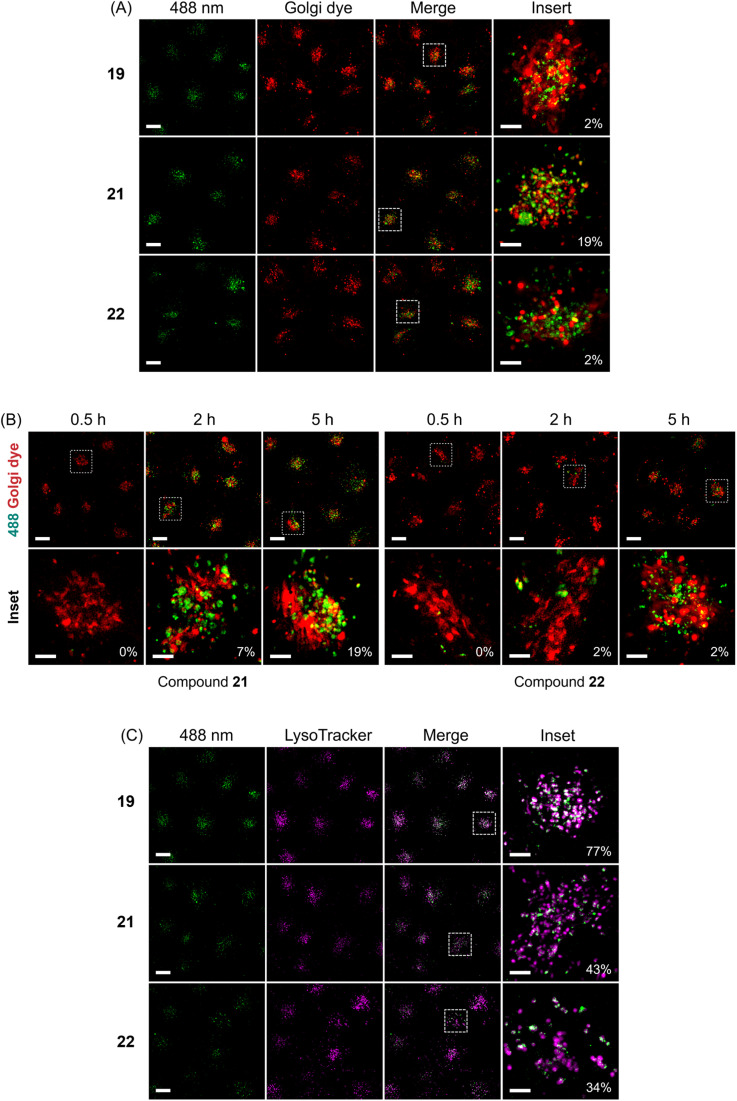
(A) Colocalization analysis of compounds (G1–NH_2_–) 19, (G2–NH_2_–) 21, (G2–guanidine–) 22 with Golgi stain — BODIPY® TR ceramide. Green dots indicate fluorescence generated from compounds. Red color indicates Golgi. The orange color indicates colocalized pixels. (B) Colocalization analysis of compounds (G2–NH_2_–) 21, (G2–guanidine–) 22 with Golgi stain — BODIPY® TR ceramide at different time points. Green dots indicate fluorescence generated from compounds. Red color indicates Golgi. The orange color indicates colocalized pixels. (C) Colocalization analysis of compounds (G1–NH_2_–) 19, (G2–NH_2_–) 21, (G2–guanidine–) 22 with LysoTracker® Deep Red. Green dots indicate fluorescence generated from compounds. Purple dots indicate lysosomes. The white color indicates colocalized pixels. HeLa cells were incubated with 50 μM of compound in the dark at 37 °C for 5 h. Scale bar for the overview represents 10 μm. Scale bar for the inset represents 1 μm. Colocalization indicated as percentage.

Another potential destination of the caged nucleotides are lysosomes. Therefore, LysoTracker® Deep Red was used for staining in colocalization experiments. The images from lysosome colocalization were analyzed and showed that 77% of (G1–NH_2_–) 19, 43% of (G2–NH_2_–) 21, and 34% of (G2–guanidine–) 22 localize to lysosomes (Fig. 4C). Thus, a considerable amount of dendronized DEAC450 caged ATPs was found in lysosomes. This might be due to the acidic nature of lysosomes,^65^ where the lipophilic basic amine and guanidine moieties of dendronized DEAC450 caged ATPs are partially trapped. This is of potential interest, as ATP is actively pumped into lysosomes to guarantee their correct function. It was recently shown that lysosomes express significant amounts of the solute carrier protein SLC17A9 and that this protein is involved in increasing lysosomal ATP concentration. This ATP can be released from astrocytes through the lysosomal pathway to sustain calcium wave propagation.^66,67^

Time-dependent lysosome colocalization analysis showed changes at different incubation times (from 0.5 h to 5 h) (ESI, Fig. S7†). The images showed that no significant amount of 21 and 22 localize to lysosomes when they are incubated for 0.5 h only. Compound 21 then quite quickly accumulated in the lysosomes (47% after 2 hours) and then remained constant (42% after 5 h). This effect was less pronounced for 22, whose localization to lysosomes increased from 23% to 34% over 2 and 5 hours, respectively. This may indicate that the uptake efficiency of 21 is overall higher.

To further investigate the uptake pathway of these compounds, we ran a time-point (1 h, 2 h, 5 h) uptake study into early endosomes/recycling endosomes using transferrin as a marker. We observe limited accumulation in this pathway and only get *ca.* 10% of colocalization after five hours (ESI, Fig. S8†). Before that, there is limited presence of the caged molecules in the endosomes. As we showed the uptake is energy dependent, this might be due to limited uptake per endocytosis event so that the signal is initially not strong enough to be distinguished from background.

Clearly, the hybrid of photocage and transporter enabled cellular uptake of ATP with different but distinct subcellular localizations. We were hence interested to also study organismal uptake and distribution since covalent tethering of transporters should in principle enable deep tissue penetration for diverse *in vivo* applications. Therefore, the tissue penetration of (G1–NH_2_–) 19, (G1–guanidine–) 20, (G2–NH_2_–) 21, (G2–guanidine–) 22 was investigated using zebrafish embryos as a model organism. Initially, we focused on 1 day old embryos, a developmental stage when the heart beats and muscles begin to contract. Compounds were added to dechorionated embryos, and after 24 h or 48 h incubation time distribution of compound fluorescence was investigated. Larvae were washed and imaging performed using a laser scanning confocal microscope. We decided to image the head of the embryo, because its diameter of 500 μm enables to observe deep tissue penetration, and compound distribution in brain and retina may be analyzed. In addition, the epithelium of the nasal placode is directly exposed to compounds in the medium. Compared to the negative control, enhanced fluorescence from the nasal placode of zebrafish was first observed when (G1–NH_2_–) 19 was applied at 28.5 °C for 24 h (Fig. 5A(c)).

**Fig. 5:**
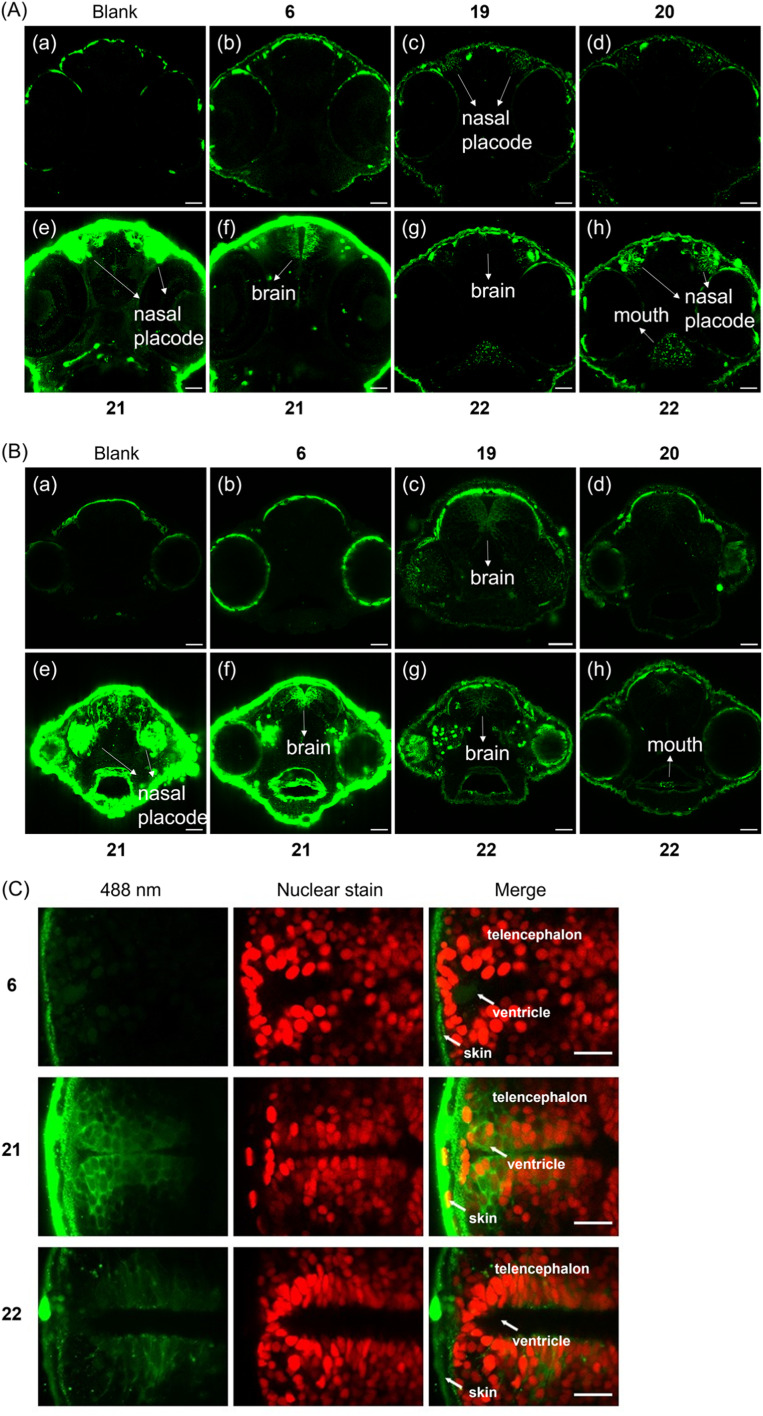
(A) Images of the heads of zebrafish embryos in tissue penetration experiments. Different cross-sections of one embryo for 21 and 22 are shown. Incubation time: 24 h. (B) Images of the heads of zebrafish embryos in tissue penetration experiments. Different cross-sections of one embryo for 21 and 22 are shown. Incubation time: 48 h. Final concentration of applied compounds: 50 μM. Green signals indicate either weak autofluorescence (see (a) blank) or the fluorescence generated from compounds. Scale bar represents 40 μm. (C) Transgenic zebrafish with *Tg(sox2: sox2-E2A-QF2)*^*m151*7^ and *Tg(QUAS: nls-mCardinal)*^*m1637*^ with red fluorescent nuclei in neural stem and progenitor cells were used and treated with 6, 21 and 22 respectively. Incubation time: 48 h. Final concentration of applied compounds: 50 μM. Temp.: 28.5 °C. Scale bar represents 20 μm.

Fig. 5A demonstrates that the simple DEAC450 modification of ATP 6 did not bestow tissue penetration properties on the nucleotide compared to the negative control. In contrast, zebrafish treated with (G2–NH_2_–) 21 after 24 h incubation showed much more intense fluorescence in the skin, nasal placode (Fig. 5A(e)) and brain (Fig. 5A(f)), compared to the zebrafish control groups (Fig. 5A(a and b)). This demonstrates that a significant amount of (G2–NH_2_–) 21 penetrated through the skin of zebrafish and entered the nasal placode and brain. Therefore, (G2–NH_2_–) 21 is able to penetrate tissue of zebrafish embryos efficiently. Likewise, zebrafish embryos incubated with (G2–guanidine–) 22 showed a fluorescence signal in the oral cavity, pharynx, nasal placode, but not in the brain (Fig. 5A(g and h)). The fluorescence signal was much less intense as compared to (G2–NH_2_–) 21. The only difference between the two molecules is the amine *versus* guanidine exchange in the dendron structure. This led to large differences in uptake efficiency. These experiments demonstrate that the uptake efficiency depends on the generation of the dendron (higher generation leads to improved uptake) and the peripheral modification of the dendron (amine is superior to guanidine at this specific timepoint in the experimental setup). Importantly, all tested compounds were found to be stable in zebrafish extracts over a period of 48 hours (Fig. S9†), underlining the efficiency of the caging approach.

Given the significant stability of the compounds, we wanted to learn if longer incubation time would contribute to deeper tissue penetration and increased uptake. With 48 h incubation, zebrafish embryos treated with (G2–NH_2_–) 21 also showed the highest relative level of fluorescence (Fig. 5B(e and f)). For the other compounds, (G1–NH_2_–) 19, (G1–guanidine–) 20, and (G2–guanidine–) 22, enhanced fluorescence in the nasal placode and brain was observed (Fig. 5B(c, d, g and h)), compared to 24 h incubation. In particular, the fluorescence in the brain significantly increased with longer incubation time. Enhanced uptake over time may be mediated through the skin, gills, oral cavity or the olfactory epithelium formed by the nasal placode (Fig. 5B(e)). The embryonic mouth starts to open at 60 hours post fertilization (hpf), potentially explaining the strong signal in the oral cavity after 48 h of incubation at 72 hpf, compared to 48 hpf (compare Fig. 5A(e and f) with Fig. 5B(e and f), and Fig. 5A(g and h), with Fig. 5B(g and h)).

Of note, 21 and 22 showed different behavior in uptake into living HeLa cells (see above, Fig. 3) and penetrating the tissue of zebrafish embryos (Fig. 5A and B). Thus, the subcellular destinations of these molecules in zebrafish are important to investigate as well. To visualize compound distribution in the brain neuroepithelium, transgenic zebrafish *Tg(sox2: sox2-E2A-QF2)*^*m1517*^ crossed with *Tg(QUAS: nls-mCardinal)*^*m1637*^ were used to label cell nuclei of neural stem and progenitor cells *in vivo* fluorescently red. Embryos were treated with 21 and 22, respectively, to investigate subcellular distribution. Fig. 5C shows that the localization of 21 in the brain of the zebrafish appear to be predominantly cytoplasmic, while 22 appears to be also associated with membranes and vesicles.

For the assays shown in Fig. 5A and B, fifteen zebrafish embryos were incubated with the respective compound of each group, and for Fig. 5C three embryos for each compound were studied. Importantly, after 24 h and 48 h of incubation, none of the embryos had died, which demonstrates that the compounds we studied are highly biocompatible, enabling organismal delivery of caged nucleotides into healthy living tissues.

In conclusion, we developed a highly modular intra(sub)cellular delivery platform for ATP (and by extension nucleoside oligophosphates). The molecule features a red-shifted photocage that is responsive to 490 nm LED irradiation within minutes and that bears a clickable residue. As a P-amidite, it can be installed to virtually any P-anhydride containing molecule. Subsequently, it can be modified using click chemistry with dendronized polyamines and guanidines for subcellular delivery and release. Importantly, due to the covalent nature, also deep tissue penetration *e.g.* into the brain of zebrafish embryos is possible, providing ample opportunities for control-of-function experiments.

## Ethical statement

All animal procedures were performed in accordance with state and federal guidelines for care and use of laboratory animals in Germany. Holding and breeding of zebrafish was approved by the Regierungspräsidium Freiburg, Germany, permit number 35-9185.64/1.1.

## Data availability

Additional datasets are available in the ESI† and from the corresponding author upon request.

## Author contributions

J. M. synthesized the molecules and evaluated their photophysical properties. J. M. contributed to fluorescence microscopy evaluations. J. M. wrote the first draft of the manuscript and prepared the figures. J. W. conducted Zebrafish embryo experiments. D. F. and L. L. conducted superresolution microscopy and colocalization studies and prepared the figures. C. Popp synthesized photocages. W. D. and R. G. planned and supervised zebrafish embryo experiments and superresolution microscopy and evaluated data. H. J. J. conceived the project and provided feedback on the draft manuscript. All authors contributed to finalizing the manuscript.

## Conflicts of interest

The authors declare no conlicts of interest.

## Supplementary Material

SC-015-D3SC05669D-s001
