# Supplementary material for: Intracellular delivery and deep tissue penetration of nucleoside triphosphates using photocleavable covalently bound dendritic polycations
Source: Chem Sci. 2024 Apr 2;15(17):6478–87. doi: 10.1039/d3sc05669d (PMC11062083; doi:10.1039/d3sc05669d)
Supplement: SC-015-D3SC05669D-s001 [file SC-015-D3SC05669D-s001.pdf]

Electronic Supplementary Material (ESI) for Chemical Science.
This journal is © The Royal Society of Chemistry 2024

Supporting Information

Intracellular Delivery and Deep Tissue Penetration of Nucleoside Triphosphates using Photocleavable Covalently Bound Dendritic Polycations

Jiahui Ma [1,2], Johanna Wehrle [2,3], Dennis Frank [2,4], Lina Lorenzen [4], Christoph Popp [1], Wolfgang Driever [2,3], Robert Grosse [2,4], Henning J. Jessen [1,2]*

Contents

1. General remarks 1
2. Supplementary figures 3
3. Live imaging 11
   3.1. Membrane staining 11
   3.2. Nucleus colocalization 11
   3.3. Lysosome colocalization 12
   3.4. Golgi colocalization 13
   3.5 Quantification of colocalization 13
   3.6 Transferrin colocalization 16
4. Flow cytometry experiments 18
5. Tissue penetration experiments 20
6. Synthesis 21
   6.1. Click chemistry 27
7. References 44
8. NMR-spectra and HRMS data 45

1. General remarks

Reagents

Reagents were purchased from commercial suppliers (Acros, Merck, *etc.*) and used without further purification, unless noted otherwise. Solvents were obtained in analytical grade and used as received for reactions, extractions, chromatography and precipitation.

Thin Layer Chromatography

Thin Layer Chromatography was carried out using Merck silica gel 60 F254 plates, visualized with UV light.

Lyophilization

Lyophilization was performed using Christ Freeze Dryer Alpha 1-4 LD+ and Zirbus Technology VaCo 5 freeze dryer.

Centrifugation

Centrifugation was performed with an Eppendorf centrifuge 5804R.

High resolution mass spectrometry (HRMS)

High resolution mass spectra were recorded by C. Warth (analytical department of the university of Freiburg, institute for organic chemistry) using a Thermo LCQ Advantage (spray voltage: 2.5 – 4.0 kV, spray current: 5 µA, ion transfer tube: 250 (150)°C, evaporation temperature: 50-400°C).

Preparative Reverse-Phase-MPLC (RP-MPLC)

Preparative Reverse-Phase-MPLC (RP-MPLC) was performed with the Flash Chromatography System PuriFlash® 5.125 from Interchim® using an Interchim® PF-C18 AQ column.

Analytical RP-HPLC-MS

Analytical RP-HPLC-MS was performed on an HPLC-MS from Thermo Scientific equipped with a Dionex UltiMate 3000 Pump, Dionex UltiMate 3000 Autosampler, Dionex UltiMate 3000 Colum Compartment, Dionex UltiMate 3000 Diode Array Detector, Dionex UltiMate 3000 Fluorescence Detector and MSQ Plus single-quadrupole mass spectrometer using an Isera ISAspher 100-3 C18 AQ, 150 × 3.0 mm column at a flow rate of 0.5 mL/min.

Nucleotides

Nucleotides were obtained as sodium salts. The corresponding tetrabutylammonium (TBA) salts of these nucleotides were obtained by cation exchange, as described later on**.**

NMR spectroscopy

The ^{1}H-, ^{13}C-, ^{31}P-NMR spectra were measured on a Bruker Avance III HD 300 MHz (282 MHz for ^{19}F, 122 MHz for ^{31}P) and on a Bruker Avance Neo 400 MHz (101 MHz for ^{13}C, 377 MHz for ^{19}F, 162 MHz for ^{31}P) NMR spectrometer. All signals were referenced to an internal solvent signal (^{1}H-NMR: $CDCl_3$: δ = 7.29 ppm, D_2O: δ = 4.79 ppm; ^{13}C-NMR: $CDCl_3$: δ = 77.16 ppm). The signals of ^{19}F- and ^{31}P-NMR spectra were referenced to an external standard. The chemical shifts are quoted in ppm. The splitting patterns are labeled as: singlet (s), broad singlet (br s), doublet (d), triplet (t), quartett (q), septet (sep), multipet (m). The coupling constants J are given in Hertz (Hz). The evaluation of NMR-spectra was done using the software MestreNova from Mestrelab Research.

2. Supplementary figures

Photophysical properties

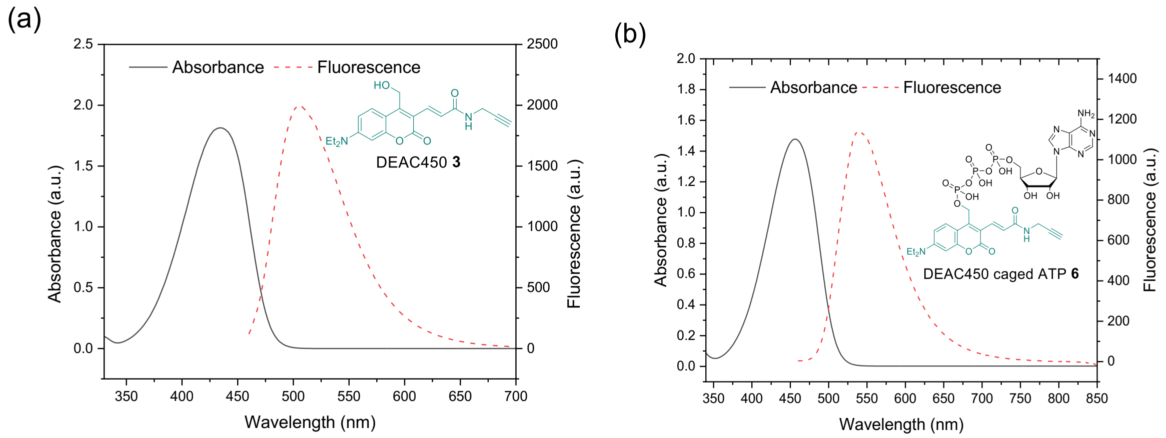

Figure S1. Absorbance and fluorescence of photocage and the caged molecule. (a) UV-absorbance and fluorescence spectra of DEAC450 **3**. Solvent: DMSO. (b) UV-absorbance and fluorescence spectra of DEAC450 caged ATP **6**. Solvent: H_2O. Concentration: Absorbance (50 µM). Fluorescence (50 nM).

Protocol of uncaging experiments

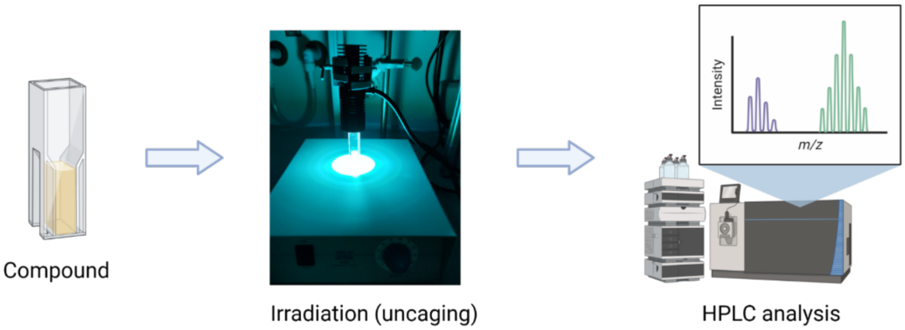

Figure S2. Uncaging setup and protocol. Created with BioRender.com.

Uncaging experiments were performed using a commercially available LED setup with a wavelength of 490 nm. (Mightex® High-Power LED Collimator Sources, Martinsried, Germany. 490 nm LED: 22 mm aperture, typical output power 140 mW. 100% light intensity.). 1 ml of 100 µM sample solution was stored in 1 cm quartz cuvette and the surface of the solution was kept around 3 cm away from light source. The cuvette was set on a Velp Scientifica™ AGE magnetic stirrer. During uncaging, the solution was stirred at highest speed. After irradiation, 40 µl of solution from experiment was analyzed by HPLC-MS (Figure S2). UV detection: 254 nm.

Uncaging kinetics

Table S1. Uncaging data extracted from HPLC analysis.

Time (min)	Peak area (mAU)	Starting material left (%)	Released ATP (%)
0	36.51	100	0
1	24.07	66	34
2	15.45	42	58
3	9.48	26	74
4	5.64	15	85
5	3.25	9	91
6	1.81	5	95
7	0.99	3	97

Flow cytometry 01

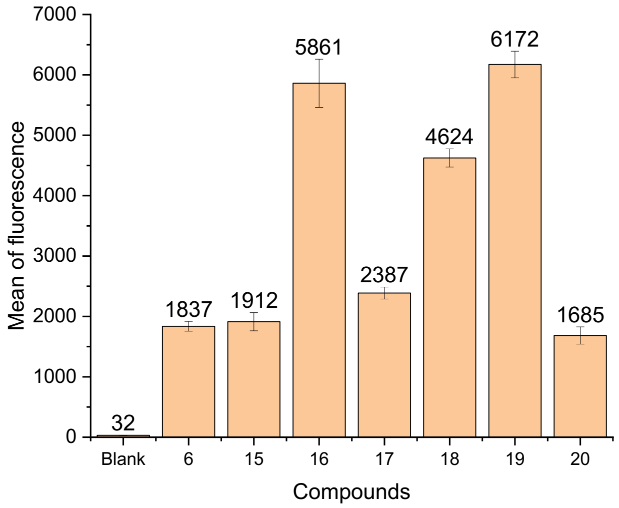

Figure S3. Mean value from FACS analysis of the fluorescence generated from the cells incubated with caged ATPs. The incubation temperature was 37°C. (Final sample concentration: 50 µM. Excitation: 488 nm.)

Stability evaluation 01

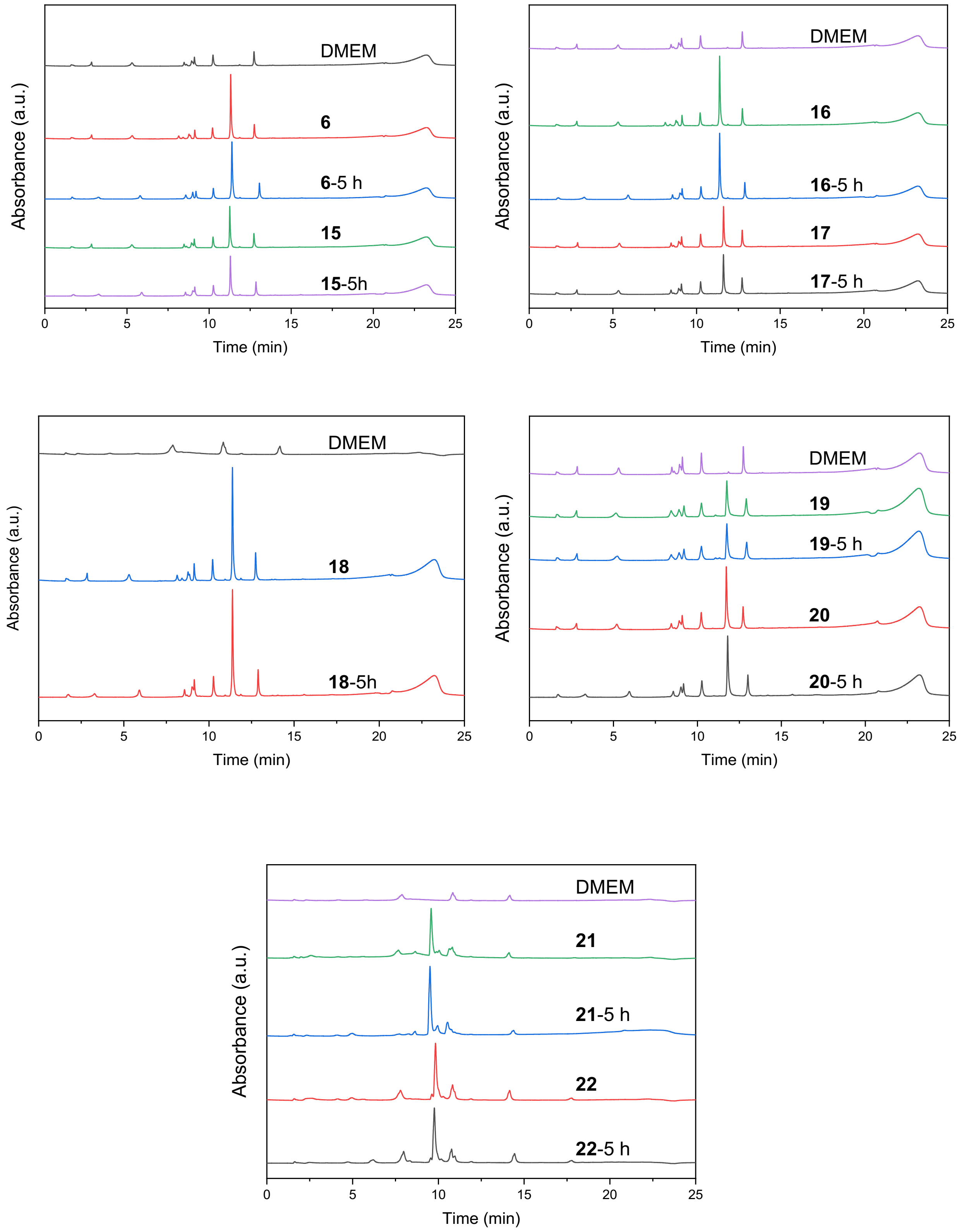

Figure S4. HPLC analysis of stability of DEAC450 caged compounds in DMEM at 37 °C in the incubator with 5% of CO_2. (Column: Pronto SIL 100-3 C18 AQ, 150 × 3.0 mm. Flow rate: 0.5 mL/min. Solvent A: water, Solvent B: CH_3CN, Solvent D: 100 mM TEAA for all compounds, except **21** and **22**. Solvent A: water with 0.1% formic acid, Solvent B: CH_3CN with 0.1% formic acid for **21** and **22** analysis.) Concentration: 50 µM.

Flow cytometry 02

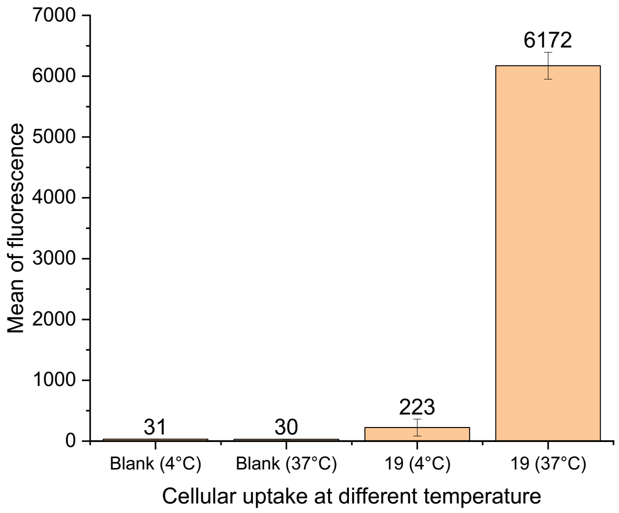

Figure S5. Mean value from flow cytometry analysis of the fluorescence generated from the cells incubated with (G1-NH2-) **19**. The incubation temperature was 4°C and 37°C. (Excitation: 488 nm.)

Nuclear colocalization experiment

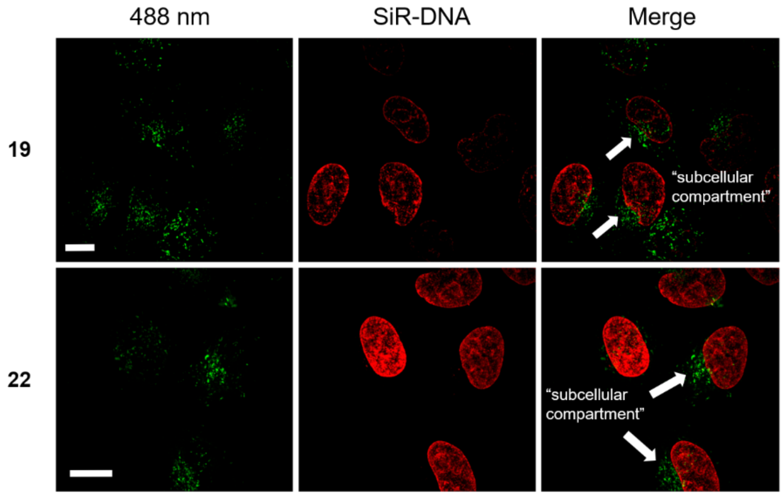

Figure S6. Colocalization analysis of (G1-NH$_2$-) **19**, (G2-guanidine-) **22** with nuclear stain — SiR-DNA. Green dots indicate fluorescence generated from compounds. Red colour indicates nucleus. Hela cells were incubated with 50 µM of compound in the dark at 37 °C for 5 h. Scale bar represents 10 µm.

Lysosome colocalization experiment

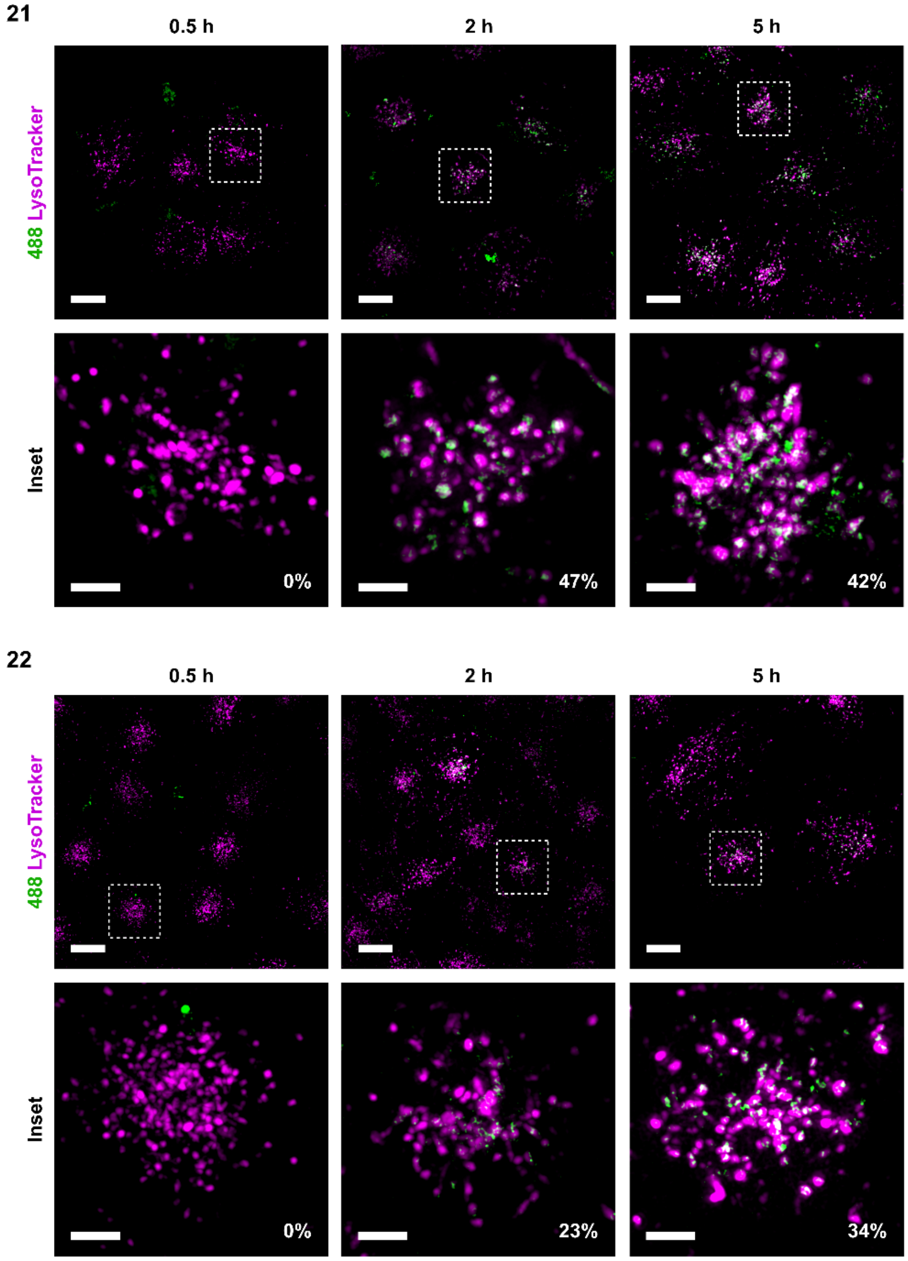

Figure S7. Colocalization analysis of compounds (G2-NH_2-) **21**, (G2-guanidine-) **22** with LysoTracker® Deep Red. Green dots indicate fluorescence generated from compounds. Purple dots indicate lysosomes. The white color indicates colocalized pixels. Hela cells were incubated with 50 µM of the compound in the dark at 37 °C. Scale bar for the overview represents 10 µm. Scale bar for the inset represents 1 µm. Colocalization indicated as percentage.

Transferrin colocalization experiment

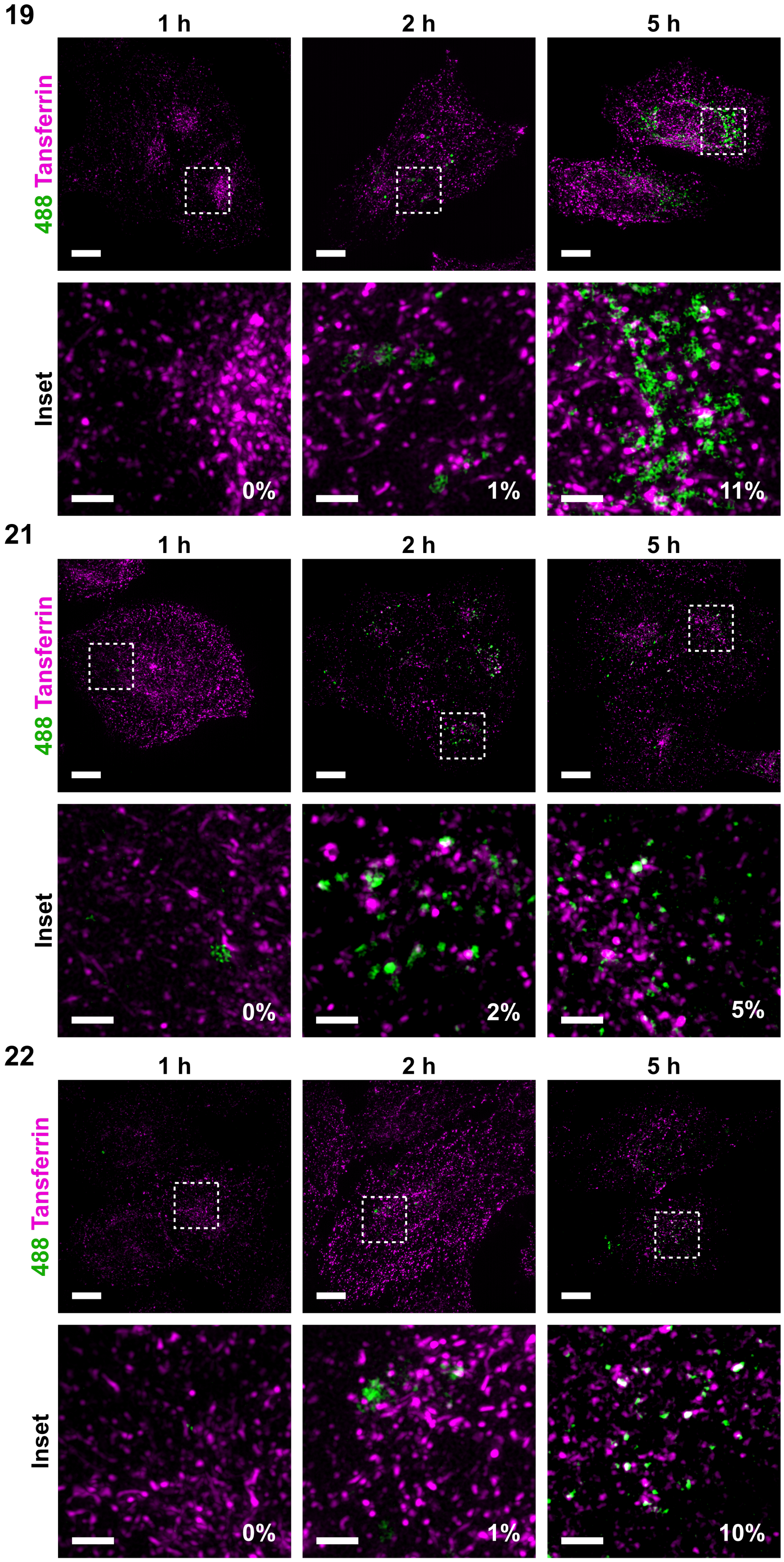

Figure S8. Colocalization analysis of compounds (G1-NH_2-) **19**, (G2-NH_2-) **21**, (G2-guanidine-) **22** with CF®640R Human Transferrin (biotium). Green dots indicate fluorescence generated from compounds. Purple dots indicate lysosomes. The white color indicates colocalized pixels. Hela cells were incubated with 50 µM of the compound in the dark at 37 °C. Scale bar for the overview represents 10 µm. Scale bar for the inset represents 1 µm. Colocalization indicated as percentage.

Stability evaluation 02

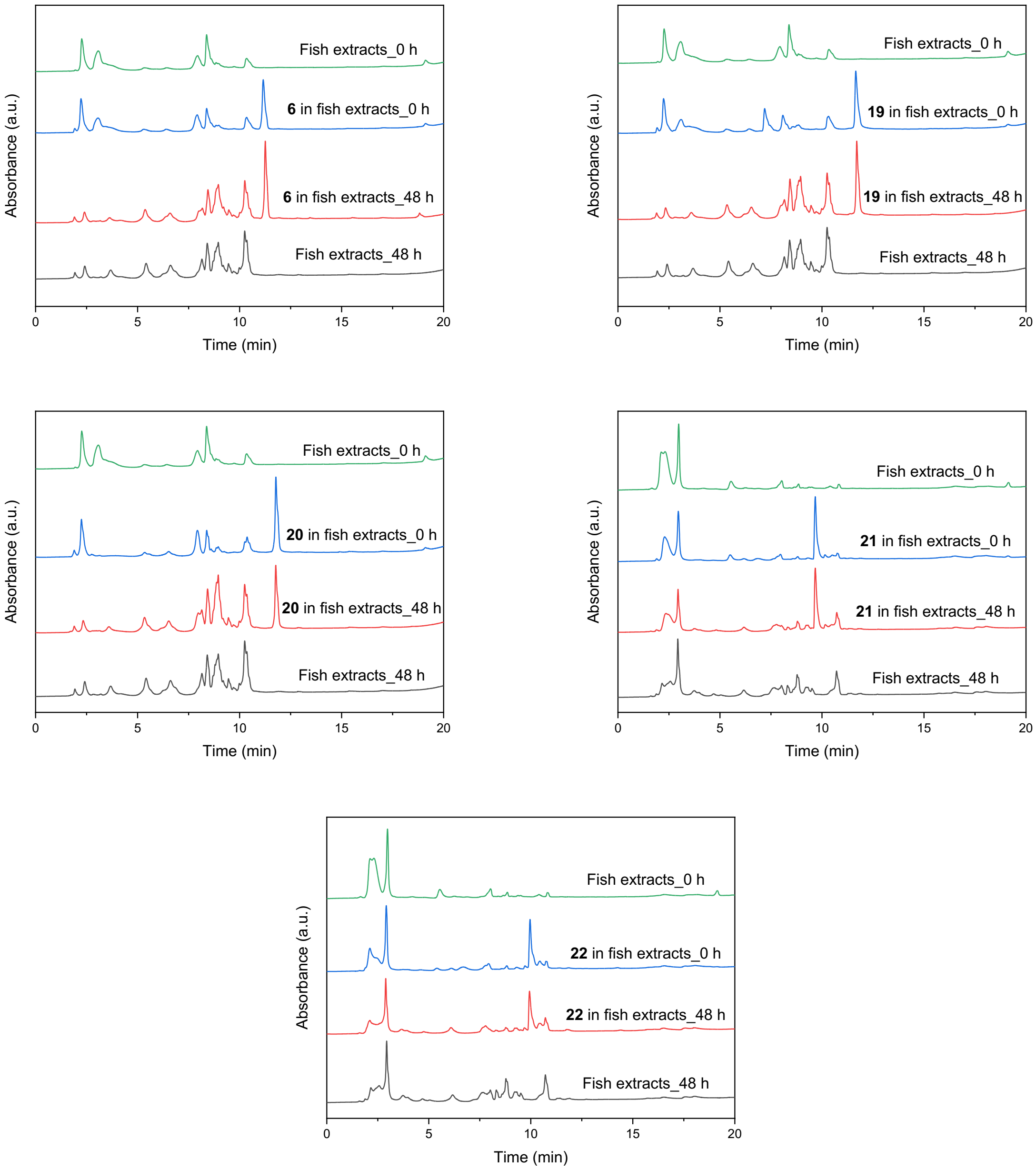

Figure S9. HPLC analysis of stability of DEAC450 caged compounds in fish extracts at 28 °C in the incubator in the dark within 48 h. (Column: Pronto SIL 100-3 C18 AQ, 150 × 3.0 mm. Flow rate: 0.5 mL/min. Solvent A: water, Solvent B: CH_3CN, Solvent D: 100 mM TEAA for **6**, **19**, **20** analysis. Concentration: 50 µM. For **21**, **22** analysis:

Solvent A: water containing 0.1% formic acid, Solvent B: CH_3CN containing 0.1% formic acid. Concentration: 100 µM.)

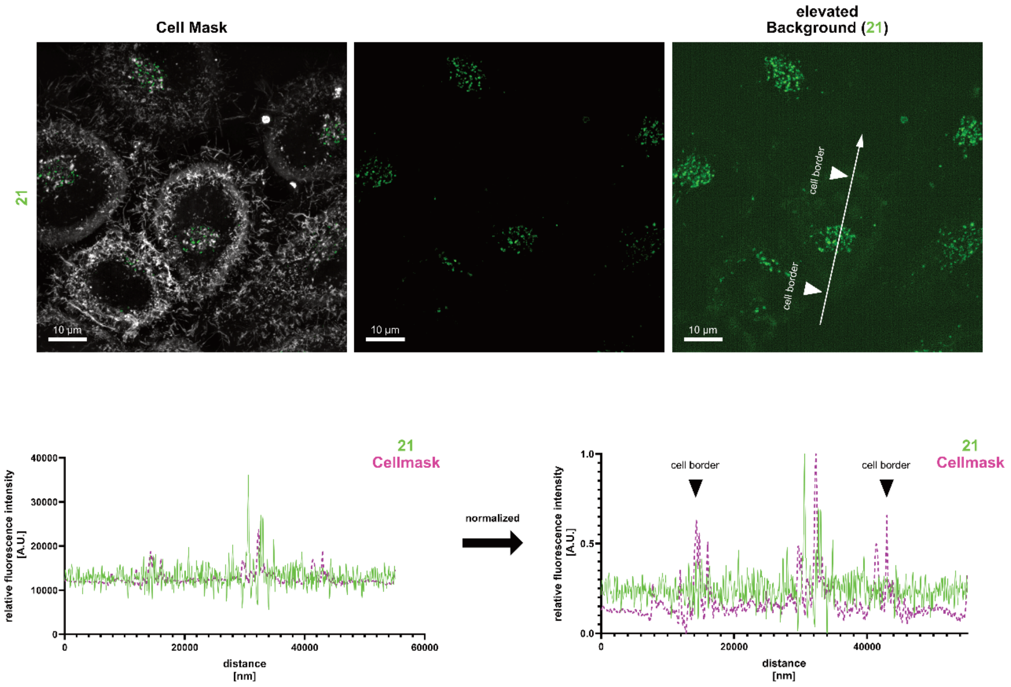

Figure S10. Fluorescence analysis.

3. Live imaging

3.1. Membrane staining

Cells grown on µ-Slide 8 Well (Ibidi) for 48h. On the day of the microscopy studies, the culture medium was replaced with serum-free medium containing 50 µM compound. Cells were then incubated at 37 °C for 5h, washed with PBS (300 ml × 2). Afterwards, 200 µl of staining solution containing CellMask™ Deep Red plasma membrane stain was added and incubated at 37 °C for 3 minutes. Subsequently, the staining solution was removed and cells were rinsed two times with DMEM (200 µl × 2). In the end, 200 µl of DMEM was added, and imaged by Elyra Super Resolution Microscope (Zeiss).

Experimental protocol

Day 1: Seed cells in µ-Slide 8 Well (Ibidi), grow for 48h.

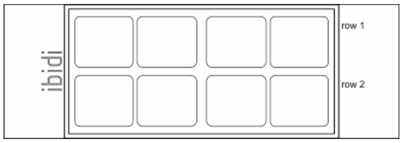

- Surface area (cm^2): 1
- Seeding density: 2.5×10^4 cells/ml
- Growth medium (µl): 300

Day 3:

1. Add compounds, incubate for 5h.
   - Sample well: 285 µl DMEM + 15 µl sample stock solution.
   - Blank well: 300 µl DMEM.
   - Final sample concentration: 50 µM.
2. Remove the medium and rinse cells two times with PBS buffer (200 µl × 2).
3. Add freshly prepared solution of the CellMask™ Deep Red plasma membrane stain in warm DMEM (1000 times dilution from commercially available stock solution.) and incubate for 3 minutes.
4. Remove the staining solution and rinse cells two times with DMEM (200 µl × 2).
5. Add 200 µl of DMEM and image.

3.2. Nucleus colocalization

Nuclear stain — SiR-DNA was used in this experiment. It has an absorption maximum at 652 nm and emission maximum at 674 nm. Hela cells were incubated with compounds for 5h. Afterwards,

medium was removed, and cells were rinsed two times with PBS buffer. Subsequently, staining solution containing SiR-DNA (1 μM) was added, incubated for 30 minutes, and then cells were imaged by Elyra Super Resolution Microscope (Zeiss).

Experimental protocol

Day 1: Seed cells in μ-Slide 8 Well (Ibidi), grow for 48h.

- Surface area (cm^2): 1
- Seeding density: 2.5×10^4 cells/ml
- Growth medium (μl): 300

Day 3:

1. Add compounds, incubate for 5h.
   - Sample well: 285 μl DMEM + 15 μl sample stock solution.
   - Blank well: 300 μl DMEM.
   - Final sample concentration: 50 μM.
2. Remove the medium and rinse cells two times with PBS buffer (200 μl × 2).
3. Add the prewarmed (37°C) probe-containing medium (300 μl) and incubate for 30 min.
4. Observe the cells using Elyra Super Resolution Microscope (Zeiss).

3.3. Lysosome colocalization

LysoTracker® deep red was used for lysosome staining. It has an absorption maximum at 647 nm and emission maximum at 668 nm. The probe is commercially available as 1 mM stock solution in anhydrous DMSO. The stock solution was diluted to the final working concentration — 50 nM in the growth medium (DMEM) for using.

Experimental protocol

Day 1: Seed cells in μ-Slide 8 Well (Ibidi), grow for 48h.

- Surface area (cm^2): 1
- Seeding density: 2.5×10^4 cells/ml
- Growth medium (μl): 300

Day 3:

1. Add compounds, incubate for 5h.
   - Sample well: 285 μl DMEM + 15 μl sample stock solution.
   - Blank well: 300 μl DMEM.

- Final sample concentration: 50 µM.

2. Remove the medium from the dish and rinse cells two times with DMEM (200 µl × 2).
3. Add the prewarmed (37°C) probe-containing medium (300 µl) and incubate the cells for 30 minutes under growth conditions.
4. Remove the loading solution, and rinse cells two times with DMEM (200 µl × 2).
5. Add DMEM (200 µl) and observe the cells using Elyra Super Resolution Microscope (Zeiss).

3.4. Golgi colocalization

BODIPY® TR ceramide was used for golgi staining. It has an absorption maximum at 589 nm and emission maximum at 617 nm. The probe is commercially available, but the received dye needs to be complexed to BSA, made as 5µM sphingolipid + 5µM BSA solution in Hanks' buffered salt solution (HBSS) + 10 mM HEPES (pH 7.4) for using.

Experimental protocol

Day 1: Seed cells in µ-Slide 8 Well (Ibidi), grow for 48h.

- Surface area (cm^2): 1
- Seeding density: 2.5×10^{4} cells/ml
- Growth medium (µl): 300

Day 3:

1. Add compounds, incubate for 5h.
    - Sample well: 285 µl DMEM + 15 µl sample stock solution.
    - Blank well: 300 µl DMEM.
    - Final sample concentration: 50 µM.
2. Remove the medium from the dish and rinse cells two times with DMEM (200 µl × 2).
3. Incubate the cells for 30 minutes at 4°C with 5 µM ceramide — BSA in HBSS/HEPES.
4. Rinse the sample three times (200 µl × 3) with ice-cold medium.
5. Incubate the cells in fresh medium at 37°C for a further 30 minutes.
6. Wash the sample two times with fresh medium (200 µl × 2) and observe the cells using Elyra Super Resolution Microscope (Zeiss).

3.5 Quantification of colocalization

Method: Golgi

Acquired Z-stracks were converted into imaris files. The 'Golgi-staining' (BODIPY® TR ceramide) channel was 3D-rendered with the 'surface' function of Imaris. The compound channel was 3D-rendered with the surface function as well. The comound surface was used as a mask over the 'Golgi-staining' channel to extract colocalizing compound with the golgi-apperatus. The extracted colocalized channel was then 3D-rendered with the same threshold values as the first 'Golgi-staining' channel. Single golgi apperatuses were selected and the total Golgi volume per cell was extracted and the ratio of colocalizing compound per golgi apperatus was calculated.

**19**	**Golgi Vol (µm)**	**Coloc Vol (µm)**	**Ratio (%)**	**Pearson's coefficient in coloc volume**	**Pearson's coefficient in whole dataset**
	135	2,97	0,0220	**-0,0683**	**0,0473**
	114	1,1	0,0096		
	151	3,27	0,0217		
	135	0,432	0,0032		
	47,4	0,39	0,0082		
	141	3,17	0,0225		
	46,4	1,39	0,0300		
	31,5	0,0731	0,0023		
**Average**	**100,1625**	**1,5994**	**0,0149**		

**21**	**Golgi Vol (µm)**	**Coloc Vol (µm)**	**Ratio (%)**	**Pearson's coefficient in coloc volume**	**Pearson's coefficient in whole dataset**
	61,6	9,33	0,1515	**-0,0389**	**0,1917**
	34,3	9,86	0,2875		
	29	5,64	0,1945		
	54,4	6,17	0,1134		
**Average**	**44,8250**	**7,7500**	**0,1867**		

22	Golgi Vol (µm)	Coloc Vol (µm)	Ratio (%)	Pearson's coefficient in coloc volume	Pearson's coefficient in whole dataset
	112	1,51	0,0135	**-0,1201**	**0,067**
	139	4,33	0,0312		
	186	5,56	0,0299		
	202	0,682	0,0034		
	146	0,631	0,0043		
	412	11,6	0,0282		
**Average**	**199,5**	**4,05216667**	**0,018396532**		

Method: Lysosomes

The 'Lysosome-staining' (LysoTracker® deep red) channel was 3D-rendered with the 'surface' function of Imaris. The compound channel was 3D-rendered with the surface function as well. The 3D-rendered lysosomes were filtered, if their volume overlaped with the volume of the 3D-rendered compound spots. These lysosomes were categorized as 'colocalizing lysosomes'. The total amound of lysosomes per cell was extracted from Imaris and the ratio of colocalizing lysosomes to the total of lysosomes per cell was calculated.

19	Number of Lysosomes	Number of Lysosomes colocalizing	Percentage of coloc Lysosomes	Pearson's coefficient in coloc volume	Pearson's coefficient in whole dataset
	209	156	0,746411483	**0,082**	**0,1537**
	135	116	0,859259259		
	134	91	0,679104478		
	112	89	0,794642857		
	107	87	0,813084112		
	121	94	0,776859504		
	74	55	0,743243243		
**Average**	**127,4285714**	**98,28571429**	**0,773229277**		

21	Number of Lysosomes	Number of Lysosomes colocalizing	Number of Lysosomes colocalizing	Pearson's coefficient in coloc volume	Pearson's coefficient in whole dataset
	127	35	0,275590551	**-0,0058**	**0,0966**
	163	83	0,509202454		
	71	39	0,549295775		
	139	58	0,417266187		
	100	56	0,56		
	68	17	0,25		
**Average**	**111,3333333**	**48**	**0,426892494**		

22	Number of Lysosomes	Number of Lysosomes colocalizing	Percentage of coloc Lysosomes	Pearson's coefficient in coloc volume	Pearson's coefficient in whole dataset
	207	69	0,3333	**-0,0247**	**0,1012**
	78	44	0,5641		
	105	25	0,2381		
	173	40	0,2312		
	128	36	0,2813		
	214	75	0,3505		
	129	50	0,3876		
**Average**	**147,7143**	**48,4286**	**0,3409**		

3.6 Transferrin colocalization

CF®640R Human Transferrin (biotium) was used for transferrin staining. It has an absorption maximum at 642 nm and emission maximum at 662 nm. HeLa cells were incubated with compounds for up to 5h. Staining solution was added 15 min before imaging. Medium containing compound and staining solution was removed and cells were rinsed twice with DMEM.

Experimental protocol

Day 1: Seed cells in µ-Slide 8 Well (Ibidi), grow for 48h.

- Surface area (cm^2): 1
- Seeding density: 2.5×10^4 cells/ml
- Growth medium (µl): 300

Day 3:

1. Add compounds, incubate for 5h.

- Sample well: 285 µl DMEM + 15 µl sample stock solution.
- Blank well: 300 µl DMEM.
- Final sample concentration: 50 µM.
- Add CF®640R Human Transferrin (25 µg/mL final concentration) 15 minutes before imaging

2. Remove the medium from the dish and rinse cells twice with DMEM
3. Observe cells using Elyra 7 Super Resolution Microscope

4. Flow cytometry experiments

Cells grown on 6 Well plates (Costar®, Corning® CellBIND® Surface) for 48h till the confluency reaches 70%–80%. On the day of the microscopy studies, the culture medium was replaced with serum-free medium containing 50 μM compound. Cells were then incubated at 37 °C for 5h, washed with PBS (1 ml × 2), and then digested with 0.25% Trypsin-EDTA (1 ml × 1) for Flow Cytometry analysis. Flow cytometer: BD Fortessa. Excitation wavelength: 488 nm.

Materials

- Trypsin, PBS
- Complete growth medium (DMEM + 10% FBS)
- Serum-free medium (DMEM)
- 15-ml conical centrifuge tubes
- Compounds in stock solution (1 mM in DMEM)

Experimental protocol

Day 1: Seed cells in six well plate, grow for 48h.

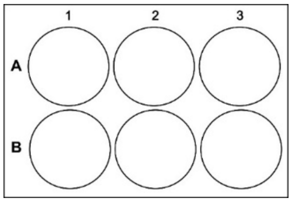

- Surface area (cm^2): 9.6
- Seeding density: 5×10^{4} cells/ml
- Cells at confluency: 1.2×10^{6}
- Trypsin volume (ml): 1
- Growth medium (ml): 2

Day 3:

1. Add compounds, incubate for 5h.
   - Sample well: 1900 μl DMEM + 100 μl sample stock solution.
   - Blank well: 2000 μl DMEM.
   - Final sample concentration: 50 μM.
2. Remove the medium and rinse two times with PBS buffer (1 ml × 2).
3. Detach cells from the plate using Trypsin (1 ml), 3 min of incubation in incubator.

4. Add 3 ml of complete growth medium and place cells into a 15 ml of conical centrifuge tube. Centrifugate the cell suspension at 200 × g for 5 minutes and discard the medium.

5. Resuspend the pellet in 1 ml of PBS and centrifugate it, discard the supernatant — PBS.

6. Resuspend the pellet in 0.5 ml of PBS for flow cytometry analysis.

19

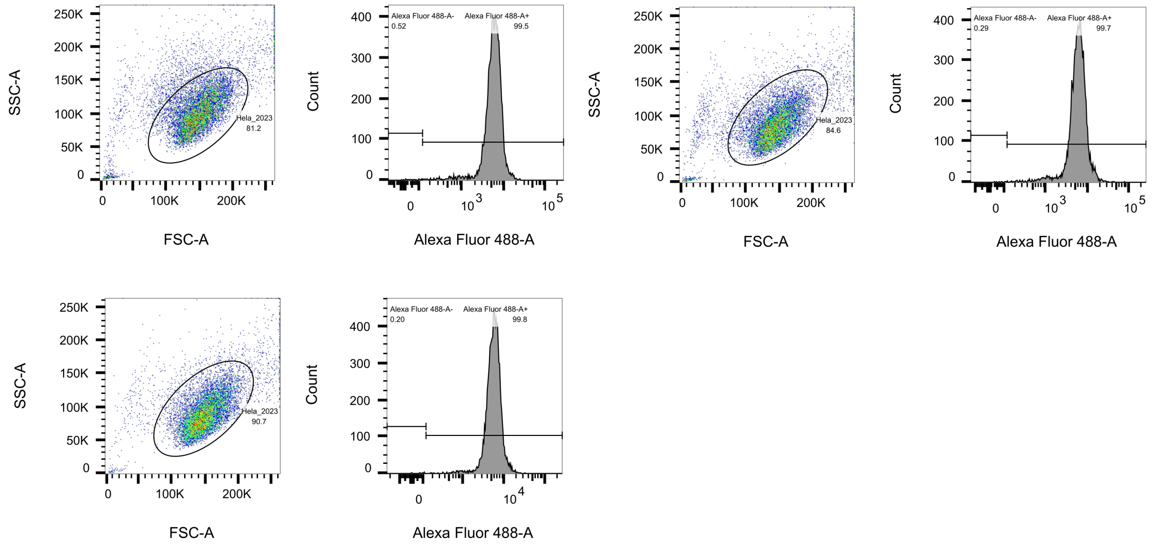

19_4°C

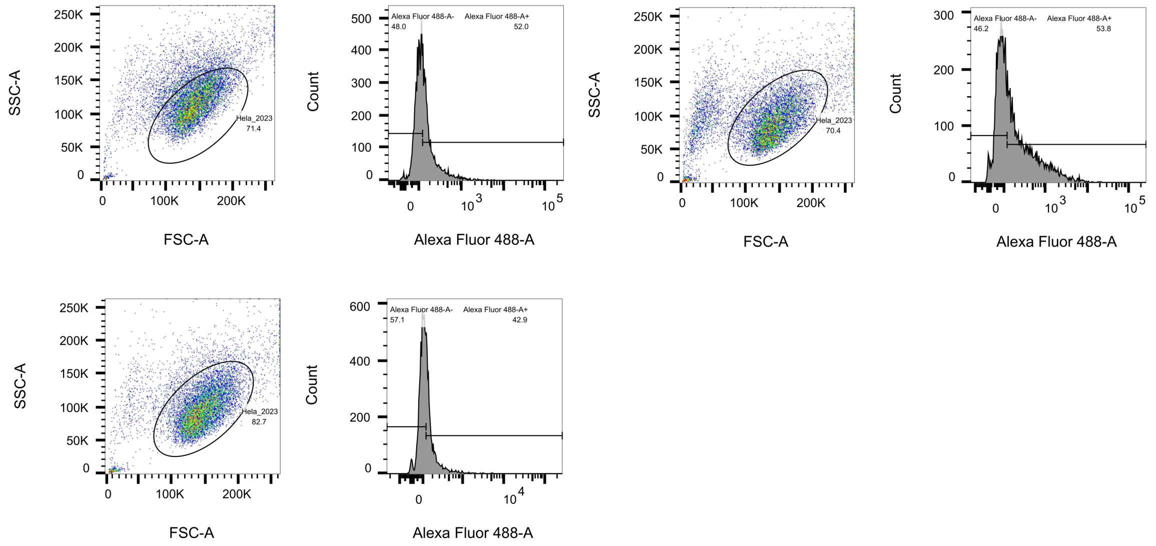

Figure S11. Results from flow cytometry measurements.

5. Tissue penetration experiments

Materials and Methods

Zebrafish breeding was performed as described by Westerfield, 1993. ABTL strain embryos were staged according to Kimmel et al., 1995 and raised at 28.5 °C in E3 medium (50 mM NaCl, 1.7mM KCl, 3.3 mM $CaCl_2$, 3.3 mM $MgSO_4$; pH 7.4) with PTU (0.2 mM) to prevent pigmentation. Tissue penetration was examined using embryos treated in E3 with 50 µM **6**, **19**, **20**, **21** (1 mM dissolved in water, diluted 1:20 in E3), or **22** (1mM dissolved in DMEM, diluted 1:20 in E3) added at 24 hours post fertilization (hpf), and incubated for 24 h and 48 h at 28.5 °C in the dark. Live embryos were embedded in 1% Agarose in E3 using a filter protecting from blue light (Red 25 Kodak Wratten; Edmund Optics Ltd #53-699). Imaging for Figure 5A, B was performed with the LSM-U-NLO/ZEISS LSM 880 confocal microscope, lens W-Plan Apochromat 20×/1,0 DIC421452-9800, Argon laser 458 nm line with 0.06 mW, 17% laser power. Imaging conditions were 512×512 pixel, 8.19 µs pixel dwell time and 2-fold line averaging.

Imaging with live fluorescent nuclear stain was achieved by using the zebrafish line *Tg(sox2: sox2-E2A-QF2*^{m1517} (a Crispr/Cas9 knockin of E2A-QF2 into the *sox2* locus to drive expression in neural stem cells; C. Altbürger and W. Driever, unpublished) crossed with *Tg(QUAS: nls-mCardinal)*^{m1637} (a QUAS responder line to drive expression of red fluorescent mCardinal in cell nuclei; C. Altbürger and W. Driever, unpublished). At 24 hpf, embryos treated in E3 with 50 µM **6**, **21** (1 mM dissolved in water, diluted 1:20 in E3), or **22** (1mM dissolved in DMEM, diluted 1:20 in E3) added at 24 hpf, and incubated for 48 h at 28.5 °C in the dark. Embedding was performed as above. Imaging was using Argon laser 488 nm wavelength with 0.38 mW, 17 % laser power, and a 633 nm diode laser. Imaging conditions: 512×512 pixel, 8.19 µs pixel dwell time, and 4-fold line averaging.

<u>Zebrafish extract</u>

Methods: Extracts of zebrafish were obtained by pooling 1000 embryos at 24 hpf in 1ml 1 × TE (10 mM Tris-Hcl, 1mM EDTA, pH 8). The protease inhibitor PMSF (1mM in Ethanol, diluted 1:100) was added. Embryos were sonicated using a Braun-Sonic 125 sonifier, centrifuged and the supernatant frozen at -80 °C. For analysis, samples were thawn and a PMSF added again at the same concentration. Compounds were added with a final concentration of 50 µM. All the samples were incubated in the incubator at 28.5 °C in the incubator in the dark for 48 h and analyzed by HPLC-UV.

6. Synthesis

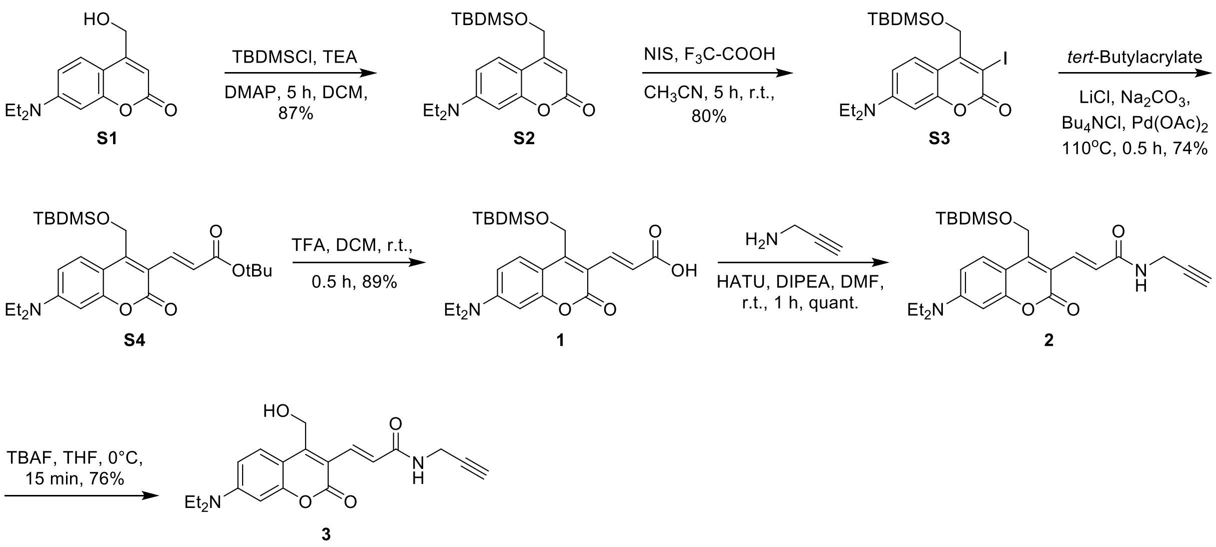

Scheme S1. Synthesis of clickable DEAC450 **3**.

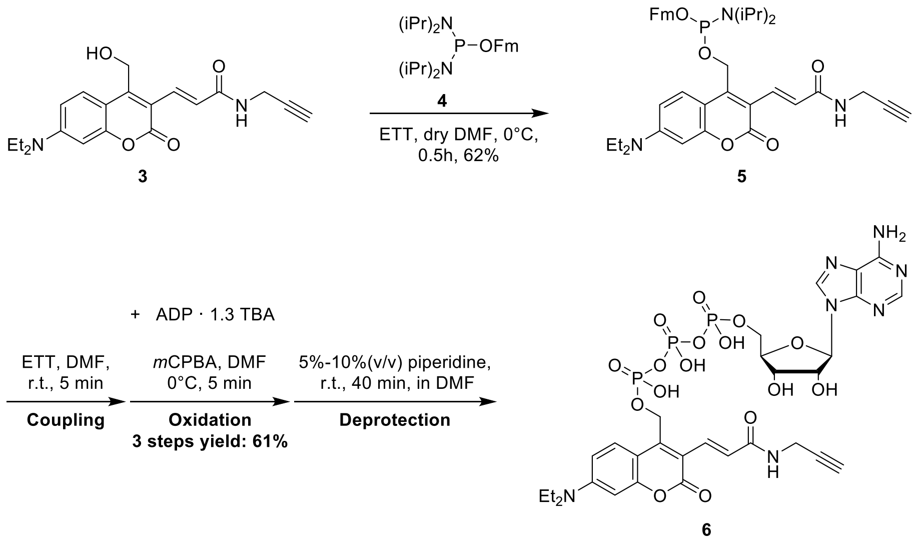

Scheme S2. Synthesis of clickable DEAC450 caged ATP **6**.

S1

S1 (DEACM) was synthesized from a previously reported procedure (Timo Weinrich, Markus Granz, Christian Grunewald, Thomas F. Prisner and Michael W. Gobel. "Synthesis of a Cytidine Phosphoramidite with Protected Nitroxide Spin Label for EPR Experiments with RNA". *Eur. J. Org. Chem.* **2017**, 491–496.[1]) and (Jiahui Ma, Alexander Ripp, Daniel Wassy, Tobias Dürr, Danye Qiu, Markus Häner, Thomas Haas, Christoph Popp, Dominik Bezold, Sabine Richert, Birgit Esser

and Henning J. Jessen. “Thiocoumarin Caged Nucleotides: Synthetic Access and Their Photophysical Properties”. *Molecules* **2020**, 25, 5325.[2])

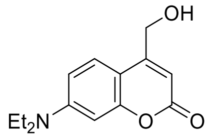

Chemical Formula: $C_{14}H_{17}NO_3$
Exact Mass: 247.1208

Analytical data was identical to the literature. **^{1}H NMR** (300 MHz, $CDCl_3$) δ 7.33 (d, J = 9.0 Hz, 1H), 6.57 (dd, J = 9.0, 2.6 Hz, 1H), 6.48 (d, J = 2.6 Hz, 1H), 6.31 – 6.26 (m, 1H), 4.87 – 4.80 (d, *J* = 4.9 Hz, 2H), 3.40 (q, J = 7.1 Hz, 4H), 3.07 – 2.94 (m, 1H), 1.20 (t, J = 7.1 Hz, 6H). **^{13}C NMR** (126 MHz, $CDCl_3$) δ 162.97, 156.12, 155.26, 150.55, 124.45, 108.68, 106.40, 105.32, 97.72, 60.90, 44.75 (2C), 12.50 (2C).

1 synthesis was modified from a previously reported procedure (Jeremy P. Olson, Hyung-Bae Kwon, Kevin T. Takasaki, Chiayu Q. Chiu, Michael J. Higley, Bernardo L. Sabatini, and Graham C. R. Ellis-Davies. “Optically Selective Two-Photon Uncaging of Glutamate at 900 nm”. *J. Am. Chem. Soc.* **2013**, 135, 5954−5957.[3]).

Acrylic acid substituted DEACM (**1**)

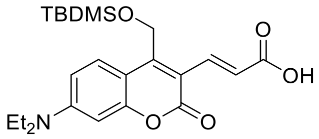

Chemical Formula: $C_{23}H_{33}NO_5Si$
Exact Mass: 431.2128

S4 (8256 mg, 17 mmol, 1 eq.) was dissolved in 120 ml of dry DCM, 72 ml of trifluoroacetic acid was added and stirred for 1h (DCM: TFA = 5: 3 (v/v)). The reaction was monitored by TLC till the reaction finished. The reaction mixture was poured into a 1 L of beaker. 120 ml of DCM was added to dilute the concentration of TFA. 200 ml of sat. $NaHCO_3$ was slowly added to the solution to remove TFA. The water phase was extracted by DCM (50 ml × 3). The organic phase was combined and dried over Na_2SO_4. Solvent was removed under reduced pressure and the residue was purified by a PF-30SIHP-F0120 column chromatography using a MPLC system. It was eluted with increasing concentration of CH_3OH in DCM from 0-10% (15 CV, 2 ×). Compound **1** (6271 mg, 14.5 mmol, 85%) was obtained as yellow solid. Rf: 0.3 (Cyclohexane/EA = 2/1). Analytical data was identical to the literature. **^{1}H NMR** (400 MHz, $CDCl_3$) δ 8.03 (d, *J* = 15.6 Hz, 1H), 7.68

(d, J = 9.3 Hz, 1H), 7.20 (d, J = 15.6 Hz, 1H), 6.66 (dd, J = 9.3, 2.6 Hz, 1H), 6.50 (d, J = 2.6 Hz, 1H), 4.95 (s, 2H), 3.47 (q, J = 7.1 Hz, 4H), 1.25 (t, J = 7.1 Hz, 6H), 0.93 (s, 9H), 0.19 (s, 6H). **^{13}C NMR** (101 MHz, $CDCl_3$) δ 172.77, 160.36, 156.19, 152.56, 151.42, 138.25, 127.54, 120.99, 112.92, 109.34, 108.27, 97.17, 57.47, 44.90 (2C), 25.78 (2C), 18.26, 12.53 (2C), -5.22 (2C).

Alkyne functionized DEAC450 (**2**)

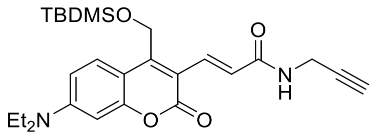

Chemical Formula: $C_{26}H_{36}N_2O_4Si$
Exact Mass: 468.2444

2 was synthesized from a general procedure for amidation using HATU, DIPEA as coupling reagents. **1** (431 mg, 1 mmol, 1 eq.) was dissolved in 5 ml of DMF, and then HATU (456 mg, 1.2 mmol, 1.2 eq.), DIPEA (168 mg, 0.2 ml, 1.3 mmol, 1.3 eq.) was added, stirred for 5 minutes. Afterwards, propargylamine (61 mg, 71 µl, 1.1 mmol, 1.1 eq.) was added and the reaction mixture was stirred at r.t for 1 h. When reaction finished, H_2O (50 ml) was added, extracted with EA (20 ml × 3). Organic lawyers were combined and concentrated, 503 mg product **2** was obtained as light-yellow solid. The yield was quantitative. (Alternative purification: The crude product was purified by a PF-30SIHP-F0040 column chromatography using a MPLC system. It was eluted with increasing concentration of CH_3OH in DCM from 0-10%). Rf: 0.8 (DCM/CH_3OH=10/1). **^{1}H NMR** (400 MHz, $CDCl_3$) δ 7.89 (d, J = 15.1 Hz, 1H), 7.72 (d, J = 9.3 Hz, 1H), 7.30 (d, J = 15.1 Hz, 1H), 6.65 (dd, J = 9.2, 2.6 Hz, 1H), 6.49 (d, J = 2.6 Hz, 1H), 6.01 (t, J = 5.2 Hz, 1H), 4.21 (dd, J = 5.3, 2.6 Hz, 2H), 3.46 (q, J = 7.1 Hz, 4H), 2.26 (t, J = 2.6 Hz, 1H), 1.27 – 1.23 (m, 6H), 0.92 (s, 9H), 0.19 (s, 6H). **^{13}C NMR** (101 MHz, $CDCl_3$) δ 166.36, 160.90, 155.80, 151.99, 151.09, 132.78, 127.71, 123.78, 112.88, 109.25, 108.43, 97.01, 79.66, 71.55, 57.52, 44.85 (2C), 29.39, 25.82 (3C), 12.53 (2C), -5.12 (2C). **HRMS** (ESI): *m/z* calcd. for $C_{26}H_{36}N_2O_4SiNa$ $[M+Na^+]^+$: 491.2337, found: 491.2332.

TBDMS deprotected alkyne functionized DEAC450 (**3**)

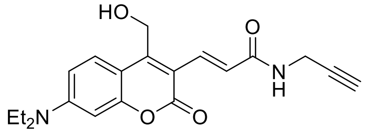

Chemical Formula: $C_{20}H_{22}N_2O_4$
Exact Mass: 354.1580

3 was synthesized applying a general procedure for deprotection of silyl ether protecting groups using TBAF. **2** (3277 mg, 7 mmol, 1 eq.) was dissolved in THF (150 ml) and stirred at 0 °C for 10 min. Afterwards, TBAF (8.4 mmol, 8.4 ml, 1.2 eq.) was added dropwise into the reaction solution and TLC was used to monitor the reaction till the reaction finished. The reaction finished in 30 min. Then 200 ml of ice H_2O was added, orange solid precipitated in the solution. The precipitation was filtered, dried over high vacuum. No further purification was needed. Compound **3** (1941 mg, 5.5 mmol, 79%) was obtained as orange solid. (Alternative work up: The solvent can be immediately removed under reduced pressure and the residue can be purified by a PF-30SIHP-F0120 (30 μm) column chromatography using a MPLC system. It can be eluted with increasing concentration of CH_3OH in DCM from 0-20%. Product came at around 7% CH_3OH in DCM.) **^{1}H NMR** (400 MHz, DMSO-*d*$_6$) δ 8.62 (t, *J* = 5.6 Hz, 1H), 7.77 (d, *J* = 9.3 Hz, 1H), 7.69 (d, *J* = 15.4 Hz, 1H), 7.13 (d, *J* = 15.4 Hz, 1H), 6.78 (dd, *J* = 9.3, 2.6 Hz, 1H), 6.54 (d, *J* = 2.6 Hz, 1H), 5.57 (t, *J* = 5.4 Hz, 1H), 4.73 (d, *J* = 5.4 Hz, 2H), 3.97 (dd, *J* = 5.6, 2.5 Hz, 2H), 3.47 (q, *J* = 7.0 Hz, 4H), 3.11 (t, *J* = 2.5 Hz, 1H), 1.14 (t, *J* = 7.0 Hz, 6H). **^{13}C NMR** (101 MHz, DMSO) δ 166.04, 160.31, 155.76, 153.22, 151.30, 132.31, 128.47, 124.77, 112.62, 109.89, 108.05, 96.71, 81.71, 73.35, 55.68, 44.58 (2C), 28.49, 12.87 (2C). **HRMS** (ESI): *m/z* calcd. for $C_{20}H_{22}N_2O_4Na$ $[M+Na^+]^+$: 377.1472, found: 377.1472.

Phosphorodiamidite (**4**)

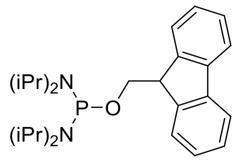

Chemical Formula: $C_{26}H_{39}N_2OP$
Exact Mass: 426.2800

9-Fluorenylmethanol (6.03 g, 30.6 mmol, 1.02 eq.) was dried for 1 h under high vacuum. Afterwards, it was dissolved in dry Et_2O/THF 5:1 (v/v; 60 ml) under argon atmosphere, dry Et_3N (4.2 ml, 30.6 mmol, 1 eq.) was added and the mixture was cooled to 0 ºC. After the addition of bis(diisopropylamino)-chlorophosphine (8.16 g, 30.6 mmol, 1.00 eq.), it was stirred at 0 ºC for 1.5 h and the formed precipitate was quickly filtered off over neutral Al_2O_3. The filtrate was concentrated under reduced pressure and immediately purified by recrystallization from pentane 20 ml. (The product containing pentane was heated till all product dissolved in it, then filtered it through filter paper, the filtrate containing flask was kept in -20 ºC freezer for 5h, the crystal formed

inside.) After filtration, the crystal was dried under reduced pressure (0.3 mbar) for 5h. Compound **4** (6.2 g, 14.5 mmol, 47%) was obtained as colorless, transparent crystals. Analytical data are consistent with those reported in the literature. **^{1}H NMR** (300 MHz, $CDCl_3$) δ 7.80 – 7.68 (m, 4H), 7.40 (td, *J* = 7.5, 1.2 Hz, 2H), 7.32 (td, *J* = 7.4, 1.2 Hz, 2H), 4.22 (t, *J* = 6.7 Hz, 1H), 3.92 (t, *J* = 6.8 Hz, 2H), 3.57 (sep, *J* = 10.7, 6.7 Hz, 4H), 1.19 (dd, *J* = 8.3, 6.8 Hz, 24H). **^{13}C NMR** (101 MHz, $CDCl_3$) δ 145.31 (2 C), 141.35 (2 C), 127.21 (2 C), 126.73 (2 C), 125.25 (2 C), 119.71 (2 C), 66.99 (d, J=22.5 Hz, 1 C), 49.78 (2 C), 49.68 (2 C), 44.56 (d, J=12.4 Hz, 1 C), 24.59 (2 C), 24.51 (2 C), 23.95 (2 C), 23.90 (2 C). **^{31}P NMR** (122 MHz, CDCl3) δ 121.85.

DEACM-450 Fm phosphoramidite (**5**)

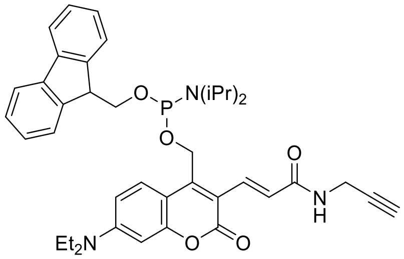

Chemical Formula: $C_{40}H_{46}N_3O_5P$
Exact Mass: 679.3175

3 (354 mg, 1.0 mmol, 1 eq.) and **4** (555 mg, 1.1 mmol, 1.1 eq.) were separately co-evaporated with dry CH_3CN (2 × 3 ml) and then dissolved in dry DMF (25 ml) with activated 3 Å molecular sieves. The mixture was cooled to 0 °C. ETT (143 mg, 1.1 mmol, 1.1 eq.) was also co-evaporated with dry CH_3CN (2 × 3 ml), then dissolved in dry DMF (3 ml), and then added to the reaction mixture. The mixture was stirred at 0 °C for 2 h. The reaction was monitored by ^{31}P-NMR. When there was no starting material **4** left, the reaction finished. The reaction mixture was poured into cold water (100 ml), yellow precipitate formed. EA was added for extraction (50 ml × 3). The organic layer was combined, washed with H_2O (30 ml × 3), and dried over Na_2SO_4, concentrated under reduced pressure. The crude product was kept in EA (5 ml) in -20 °C freezer overnight. Pure product formed in the solution as yellow solid. The solid was filtered and dried by a high vacuum. Product **5** was obtained as light-yellow power (467 mg, 62%). Rf: 0.5 (Cyclohexane/EA = 2/1). Note: Residue in solution was purified by a PF-30SIHP-F0040 (30 µm) column chromatography using a MPLC system. It was eluted with increasing concentration of EA in cyclohexane from 0-100%. Product came at around 50% EA in cyclohexane. **^{31}P NMR** (122 MHz, $CDCl_3$) δ 149.22. **^{1}H NMR** (400 MHz, $CDCl_3$) δ 7.91 (d, *J* = 15.0 Hz, 1H), 7.74 (ddt, *J* = 7.6, 4.6, 1.0 Hz, 2H), 7.65 (d, *J* = 9.2 Hz,

1H), 7.63 – 7.60 (m, 1H), 7.55 (dq, J = 7.5, 0.9 Hz, 1H), 7.37 (tdt, J = 7.5, 4.0, 0.9 Hz, 2H), 7.27 – 7.23 (m, 3H), 6.54 (dd, J = 9.2, 2.6 Hz, 1H), 6.48 (d, J = 2.6 Hz, 1H), 5.94 (t, J = 5.3 Hz, 1H), 4.93 – 4.86 (m, 2H), 4.22 – 4.10 (m, 3H), 4.04 (dt, J = 9.9, 6.6 Hz, 1H), 3.82 (dt, J = 9.9, 7.3 Hz, 1H), 3.66 (dhept, J = 10.4, 6.8 Hz, 2H), 3.43 (q, J = 7.1 Hz, 4H), 2.24 (t, J = 2.5 Hz, 1H), 1.24 – 1.20 (m, 12H), 1.16 (d, J = 6.8 Hz, 6H). **^{13}C NMR** (101 MHz, $CDCl_3$) δ 166.18, 160.81, 155.70, 151.05, 150.47 (d, J = 8.6 Hz, 1C), 144.95, 144.48, 141.38, 141.21, 132.87, 127.72, 127.70, 127.35, 126.83, 126.82, 125.47, 125.13, 124.20, 119.77, 119.70, 113.68, 109.22, 108.39, 97.01, 79.71, 71.52, 66.14 (d, J = 17.1 Hz, 1C), 57.13 (d, J = 18.5 Hz, 1C), 53.45, 49.06 (d, J = 7.6 Hz, 1C), 44.86 (2C), 43.32 (d, J = 12.3 Hz, 1C), 29.37, 24.80, 24.73, 24.65, 24.57, 12.52 (2C). **HRMS** (ESI): *m/z* calcd. for $C_{40}H_{47}N_3O_5P$ $[M+H^+]^+$: 680.3248, found: 680.3251.

DEAC450 caged ATP (**6**)

6 was synthesized from a previously reported procedure by Alexandre Hofer.[4] With the developed method, ADP was coupled to clickable DEACM-450 Fm phosphoramidite **5**. In this step, ADP was converted to a mixed P(III)-P(V) intermediate. Then *m*CPBA was used to oxidize P(III) to P(V), and piperidine was added to remove Fm protecting group.

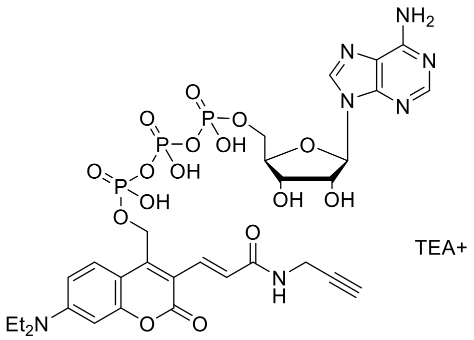

Chemical Formula: $C_{30}H_{36}N_7O_{16}P_3$
Exact Mass: 843.1431

ADP · 1.3 TBA (240 mg, 330 µmol, 1 eq.) and ETT (430 mg, 3.3 mmol, 10 eq.) were separately co-evaporated with dry CH_3CN (2 × 2 ml) and then dissolved in 5 ml of dry DMF. P-amidite **5** (269 mg, 396 µmol, 1.2 eq.) was added to the reaction mixture and stirred for 10 minutes at r.t. Afterwards, the solution was cooled to 0 °C, using an ice bath. *m*CPBA (57 mg, 330 µmol, 1 eq.) was added. After 10 minutes, piperidine (0.5 ml) was added and the mixture was stirred for 30 minutes at r.t. The product was then precipitated by adding the reaction mixture dropwise to a cold diethyl ether solution (3 ml of reaction mixture to 35 ml of diethyl ether solution). The precipitate was isolated via centrifugation (7500 rpm, 5 min, 0 °C) and the oily solid was purified by a PF-

C18AQ-F0040 (30 µm) column chromatography using a MPLC system (ELSD detection, no UV detection). It was eluted with increasing concentration of CH_3CN in H_2O from 0-50% using TEAA as buffer. Product came at around 20% CH_3CN in H_2O. The product containing fractions, were combined and lyophilized. Compound **6** (267 mg, ATP · 4.6 TEA^+, 0.2 mmol, 61%) was obtained as orange solid. **^{31}P NMR** (162 MHz, D_2O) δ -11.48 (d, *J* = 19.7 Hz), -12.02 (d, *J* = 19.1 Hz), -23.15 (t, *J* = 19.5 Hz). **^{1}H NMR** (400 MHz, D_2O) δ 8.16 (s, 1H), 7.94 (s, 1H), 7.49 – 7.40 (m, 2H), 6.74 (d, *J* = 15.8 Hz, 1H), 6.62 (dd, *J* = 9.3, 2.6 Hz, 1H), 6.24 (d, *J* = 2.6 Hz, 1H), 5.73 – 5.65 (m, 1H), 4.92 – 4.86 (m, 2H), 4.38 – 4.33 (m, 2H), 4.32 – 4.26 (m, 1H), 4.18 – 4.10 (m, 2H), 4.02 (dd, *J* = 2.5, 1.7 Hz, 2H), 3.36 (q, *J* = 7.2 Hz, 4H), 2.56 (t, *J* = 2.5 Hz, 1H), 1.12 (t, *J* = 7.1 Hz, 6H). **^{13}C NMR** (101 MHz, D_2O) δ168.44, 162.90, 154.76, 154.56, 151.90, 151.60, 148.51 (d, J = 9.9 Hz, 1C), 147.93, 138.92, 132.70, 127.39, 123.55, 117.93, 112.39, 110.60, 107.32, 95.94, 86.92, 83.10 (d, J = 9.7 Hz, 1C), 79.80, 74.84, 71.74, 69.61, 64.86 (d, J = 5.6 Hz, 1C), 59.73 (d, J = 5.0 Hz, 1C), 44.62 (2C), 28.94, 11.85 (2C). **HRMS** (ESI): *m/z* calcd. for $C_{30}H_{34}N_7O_{16}P_3$ $[M-2H^+]^{2-}$: 420.5643, found: 420.5642 (Z=2).

6.1. Click chemistry

Introduction

Click chemistry, specifically Cu(I)-catalyzed azide-alkyne cycloaddition (CuAAC) was used to introduce Azido-PAMAM-dendrons and their guanidino derivatives to DEAC450 caged ATP **6**. Most reactions were performed in a scale of around 20 mg of caged ATP **6** (1 eq.), and Azido moiety (1.5 eq.), copper(II) sulfate (0.5 eq.), sodium L-ascorbate (1.0 eq.) were used. All the reactions were conducted at room temperature and monitored by HPLC-MS and HPLC-UV. Basically, 1 ml of H_2O was used for the reaction, Azido moiety was dissolved in 1 ml of THF or DMSO or CH_3OH and added to reaction mixture. Reaction time was around 1 h.

After the reaction finished, THF or CH_3OH was removed under vacuum (DMSO was not removed), and the resulting solution was purified by a PF-C18AQ-F0040 (30 µm) column chromatography using a MPLC system (ELSD detection, no UV detection). It was eluted with increasing concentration of CH_3CN in H_2O from 0-50% using TEAA as buffer. Products came at around 20-30% CH_3CN in H_2O. The product containing fractions were combined and lyophilized. Copper was removed by Chelex® 100 sodium form (10 mg Chelex® 100 was added to sample solution, stirred for 10 min, repeated for 3 times.). TEA^+ was also removed by Chelex® 100.

Synthesis of **7**, **8** and their click reactions.

7, 8 was synthesized from a previously reported procedure (Rujing Zhang, Nan Zheng, Ziyuan Song, Lichen Yin, Jianjun Cheng. “The effect of side-chain functionality and hydrophobicity on the gene delivery capabilities of cationic helical polypeptides”. *Biomaterials.* 35. **2014,** 3443-3454.[5])

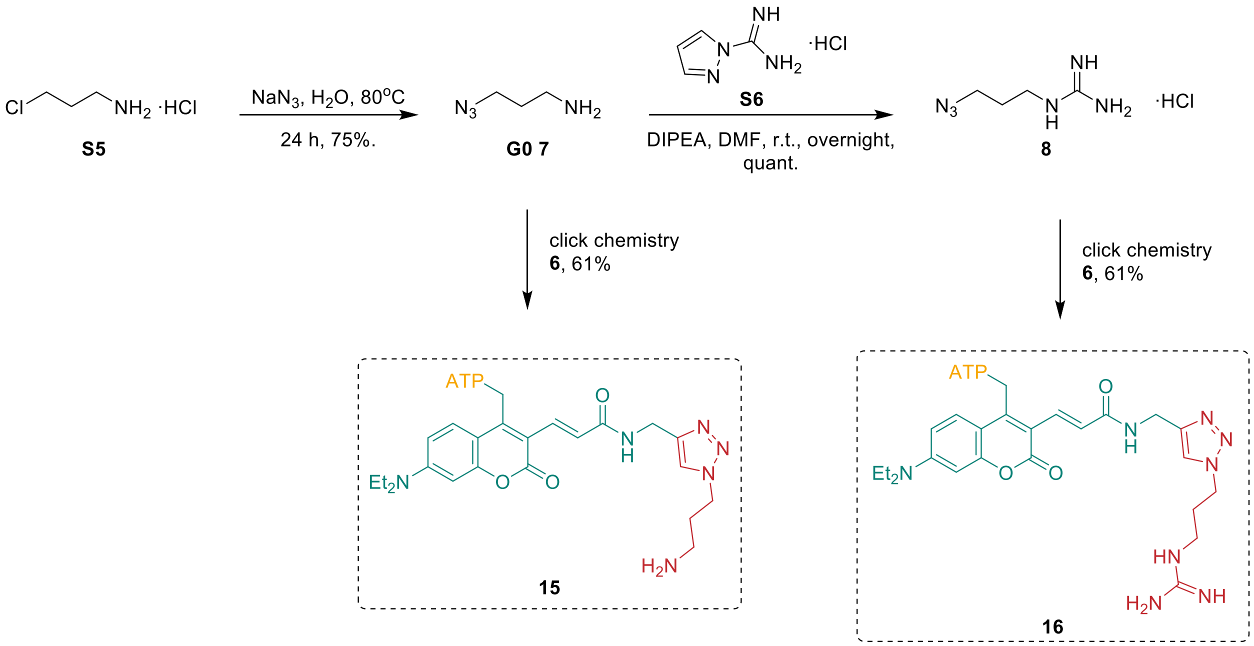

Scheme S3. Synthesis of G0 **7**, its guanidinium derivative **7** and clickable DEAC450 caged ATPs with different transporters attached **15**, **16**.

3-azidopropan-1-amine (**7**)

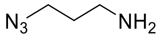

Chemical Formula: $C_3H_8N_4$
Exact Mass: 100.0749

3-Chloropropylamine hydrochloride **S5** (5.0 g, 38.5 mmol, 1 eq.) and sodium azide (7.5 g, 115.5 mmol, 3 eq.) were added into 60 ml ultrapure water and the solution was stirred at 80 °C for 20 h. After the reaction mixture cooled down in an ice-water bath to room temperature, sodium hydroxide (2.5 g) was added into the solution. The solution was extracted with diethyl ether (4 × 50 ml), and then the combined organic layer was dried by sodium sulfate. Diethyl ether was removed at 25 °C under reduced pressure (650 mbar) with rotavap within 10 h (**Note:** The boiling point of **7** is 50 °C). Compound **7** was obtained (4.2 g, 42 mmol, quant.) as light-yellow liquid (**Note:** This compound is potentially explosive, so the solvent diethyl ether was not completely

removed on rotovap. In the end, the quantification of diethyl ether can be realized by ^{1}**H** NMR spectrum. In this case, the amount of product **7**/diethyl ether = 3/2). Analytical data is consistent with those reported in the literature. **^{1}H NMR** (300 MHz, $CDCl_3$) δ 3.39 (t, *J* = 6.7 Hz, 2H), 2.82 (t, *J* = 6.8 Hz, 2H), 1.75 (p, *J* = 6.8 Hz, 2H). **HRMS** (ESI): *m/z* calcd. for $C_3H_9N_4$ $[M+H^+]^+$: 101.0822, found: 101.0821.

1-(3-azidopropyl)guanidine (**8**)

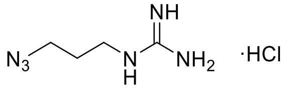

Chemical Formula: $C_4H_{10}N_6$
Exact Mass: 142.0967

Azidopropylamine **7** (200 mg, 2 mmol, 1 eq.), H-pyrazole-1-carboxamidine hydrochloride **S6** (293 mg, 2 mmol, 1 eq.) and DIPEA (284 mg, 0.4 ml, 2.2 mmol, 1.1 eq.) were dissolved in dry DMF (3 ml) and stirred overnight at r.t.. After the reaction finished, ether (30 ml) was added. The crude product was precipitated, collected, washed with ether, dried over vacuum. The yield was quant. Analytical data is identical to the literature. **^{1}H NMR** (300 MHz, D_2O) δ 3.37 (t, *J* = 6.6 Hz, 2H), 3.22 (t, *J* = 6.8 Hz, 2H), 1.84 – 1.74 (m, 2H). **HRMS** (ESI): *m/z* calcd. for $C_4H_{11}N_6$ $[M+1H^+]^+$: 143.1040, found: 143.1040.

($G0-NH_2$-) (**15**)

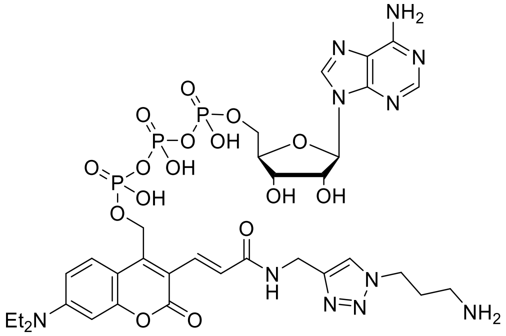

Chemical Formula: $C_{33}H_{44}N_{11}O_{16}P_3$
Exact Mass: 943.2180

15 was synthesized by click reaction between caged ATP **6** and compound **7**. Caged ATP **6** (20 mg, 17 µmol, 1 eq.) was used for the reaction. Compound **15** (13 mg, 82%) was obtained as orange solid. **^{31}P NMR** (162 MHz, D_2O) δ -11.39 (d, *J* = 19.9 Hz), -11.82 (d, *J* = 19.1 Hz), -23.07 (t, *J* = 19.5 Hz). **^{1}H NMR** (400 MHz, D_2O) δ 8.14 (s, 1H), 8.05 (s, 1H), 7.94 (s, 1H), 7.45 (d, *J* = 15.9 Hz, 1H), 7.41 (d, *J* = 9.3 Hz, 1H), 6.78 (d, *J* = 15.9 Hz, 1H), 6.60 (dd, *J* = 9.3, 2.5 Hz, 1H), 6.23 (d, *J*

= 2.5 Hz, 1H), 5.74 – 5.68 (m, 1H), 4.95 – 4.84 (m, 2H), 4.56 (d, *J* = 4.3 Hz, 2H), 4.52 (t, *J* = 6.7 Hz, 2H), 4.39 – 4.33 (m, 2H), 4.26 (ddd, *J* = 11.2, 4.1, 2.2 Hz, 1H), 4.17 – 4.06 (m, 2H), 3.36 (q, *J* = 7.1 Hz, 4H), 2.95 – 2.86 (m, 2H), 2.25 (ddd, *J* = 12.5, 8.5, 6.4 Hz, 2H), 1.14 (t, *J* = 7.1 Hz, 6H). **HRMS** (ESI): *m/z* calcd. for $C_{33}H_{43}N_{11}O_{16}P_3$ $[M-1H^+]^-$: 942.2108, found: 942.2109.

(G0-guanidine-) (**16**)

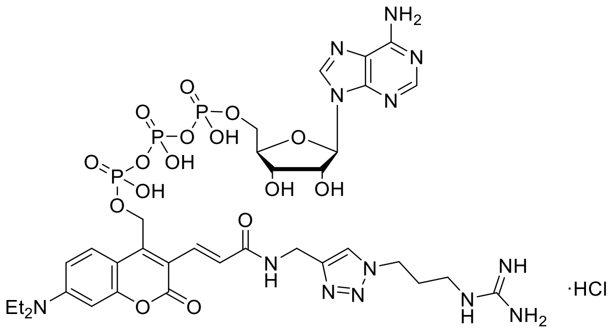

Chemical Formula: $C_{34}H_{46}N_{13}O_{16}P_3$
Exact Mass: 985.2398

16 was synthesized by click reaction between caged ATP **6** and compound **8**. Caged ATP **6** (20 mg, 15 µmol, 1 eq.) was used for the reaction. Compound **16** (9 mg, 61%) was obtained as orange solid. **^{31}P NMR** (162 MHz, D_2O) δ -11.38 (d, *J* = 19.8 Hz), -11.82 (d, *J* = 19.3 Hz), -23.03 (t, *J* = 19.5 Hz). **^{1}H NMR** (400 MHz, D_2O) δ 8.15 (s, 1H), 8.02 (s, 1H), 7.95 (s, 1H), 7.48 (d, *J* = 15.9 Hz, 1H), 7.43 (d, *J* = 9.3 Hz, 1H), 6.80 (d, *J* = 15.9 Hz, 1H), 6.61 (dd, *J* = 9.4, 2.6 Hz, 1H), 6.24 (d, *J* = 2.5 Hz, 1H), 5.75 – 5.68 (m, 1H), 4.98 – 4.86 (m, 2H), 4.56 (s, 2H), 4.52 – 4.46 (m, 2H), 4.39 – 4.33 (m, 2H), 4.31 – 4.24 (m, 1H), 4.18 – 4.09 (m, 2H), 3.37 (q, *J* = 7.1 Hz, 4H), 3.15 (t, *J* = 6.4 Hz, 2H), 2.19 (p, *J* = 6.4 Hz, 2H), 1.14 (t, *J* = 7.1 Hz, 6H). **HRMS** (ESI): *m/z* calcd. for $C_{34}H_{45}N_{13}O_{16}P_3$ $[M-1H^+]^-$: 984.2326, found: 984.2323. *m/z* calcd. for $C_{34}H_{47}N_{13}O_{16}P_3$ $[M+1H^+]^+$: 986.2471, found: 986.2484.

Synthesis of **9**, **10** and their click reactions.

9 was synthesized from a previously reported procedure (Camille Romuald, Eric Busseron, and Frédéric Coutrot. “Very Contracted to Extended *co*-Conformations with or without Oscillations in Two- and Three-Station [c2]Daisy Chains Camille”.[6]) **10** was synthesized from a previously reported procedure (Rujing Zhang, Nan Zheng, Ziyuan Song, Lichen Yin, Jianjun Cheng. “The effect of side-chain functionality and hydrophobicity on the gene delivery capabilities of cationic helical polypeptides Rujing”.[5])

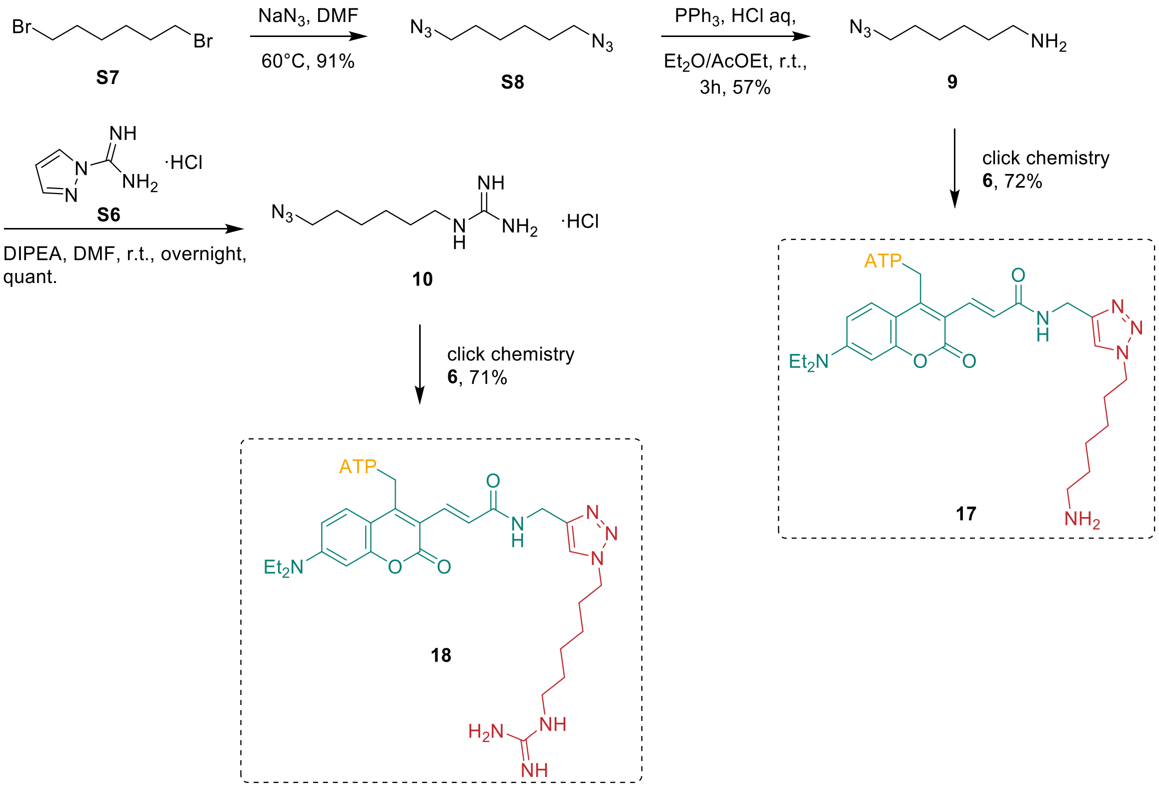

Scheme S4. Synthesis of **9**, its guanidinium derivative **10** and clickable DEAC450 caged ATPs with different transporters attached **17**, **18**.

6-azidohexan-1-amine (**9**)

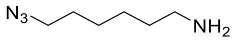

Chemical Formula: $C_6H_{14}N_4$
Exact Mass: 142.1218

Yield = 57%. Analytical data was identical to the literature. **^{1}H NMR** (400 MHz, $CDCl_3$) δ 3.28 (t, *J* = 6.9 Hz, 2H), 2.74 – 2.68 (m, 2H), 1.67 – 1.57 (m, 2H), 1.53 – 1.31 (m, 6H). **^{13}C NMR** (101 MHz, $CDCl_3$) δ 51.42, 42.11, 33.65, 28.83, 26.61, 26.45.

1-(6-azidohexyl)guanidine (**10**)

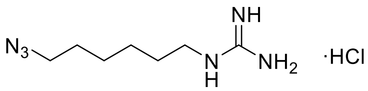

Chemical Formula: $C_7H_{16}N_6$
Exact Mass: 184.1436

Yield = 92%. Analytical data was identical to the literature. **^{1}H NMR** (300 MHz, $CDCl_3$) δ 3.31 (t, *J* = 6.8 Hz, 2H), 3.18 (q, *J* = 6.6 Hz, 2H), 1.63 (q, *J* = 5.9, 4.5 Hz, 4H), 1.45 (dt, *J* = 6.8, 4.1 Hz, 4H). **HRMS** (ESI): *m/z* calcd. for $C_7H_{17}N_6$ $[M+1H^+]^+$: 185.1509, found: 185.1510.

(G0-C_6-NH_2-) (**17**)

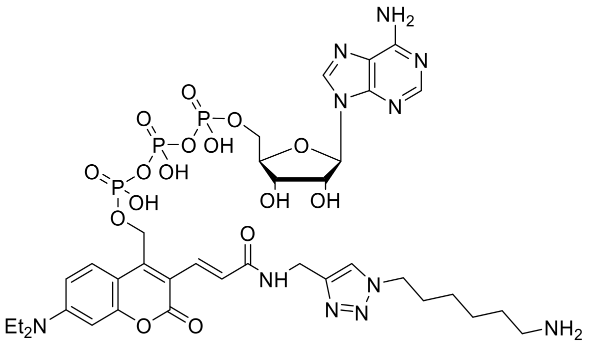

Chemical Formula: $C_{36}H_{50}N_{11}O_{16}P_3$
Exact Mass: 985.2650

17 was synthesized by click reaction between caged ATP **6** and compound **9**. Caged ATP **6** (26 mg, 29 µmol, 1 eq.) was used for the reaction. Compound **17** (21 mg, 72%) was obtained as yellow solid. **^{31}P NMR** (162 MHz, D_2O) δ -11.36 (d, *J* = 19.2 Hz), -11.81 (d, *J* = 19.1 Hz), -22.97 (t, *J* = 19.3 Hz). **^{1}H NMR** (400 MHz, D_2O) δ 8.24 (s, 1H), 8.04 (s, 1H), 8.03 (s, 1H), 7.60 – 7.48 (m, 2H), 6.90 (d, *J* = 15.8 Hz, 1H), 6.69 (dd, *J* = 9.4, 2.6 Hz, 1H), 6.31 (d, *J* = 2.5 Hz, 1H), 5.83 – 5.75 (m, 1H), 5.06 – 4.95 (m, 2H), 4.65 – 4.61 (m, 2H), 4.49 – 4.42 (m, 4H), 4.41 – 4.32 (m, 1H), 4.21 (ddt, *J* = 8.1, 5.2, 2.6 Hz, 2H), 3.45 (q, *J* = 7.1 Hz, 4H), 2.97 – 2.89 (m, 2H), 1.99 – 1.88 (m, 2H), 1.68 – 1.56 (m, 2H), 1.49 – 1.24 (m, 4H), 1.22 (t, *J* = 7.1 Hz, 6H). **HRMS** (ESI): *m/z* calcd. for $C_{36}H_{51}N_{11}O_{16}P_3$ $[M+1H^+]^+$: 986.2723, found: 986.2735; *m/z* calcd. for $C_{36}H_{48}N_{11}O_{16}P_3$ $[M-2H^+]^{2-}$: 491.6252, found: 491.6253 (Z=2).

(G0-C_6-guanidine-) (**18**)

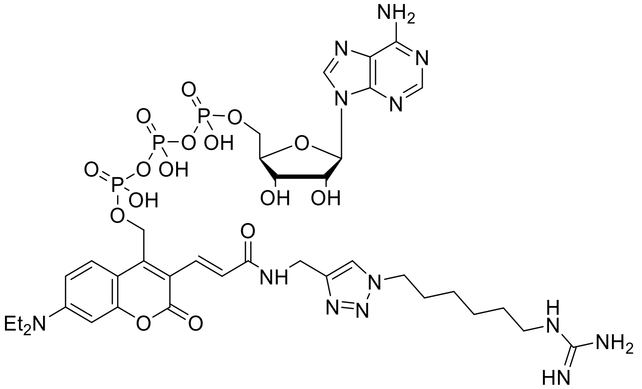

Chemical Formula: $C_{37}H_{52}N_{13}O_{16}P_3$
Exact Mass: 1027.2868

18 was synthesized by click reaction between caged ATP **6** and compound **10**. Caged ATP **6** (28 mg, 24 µmol, 1 eq.) was used for the reaction. Compound **18** (17 mg, 71%) was obtained as yellow

solid. **^{31}P NMR** (162 MHz, D_2O) δ -11.35 (d, *J* = 19.3 Hz), -11.86 (d, *J* = 19.0 Hz), -22.93 (t, *J* = 19.3 Hz). **^{1}H NMR** (400 MHz, D_2O) δ 8.16 (s, 1H), 7.94 (s, 1H), 7.93 (s, 1H), 7.46 – 7.41 (m, 2H), 6.80 (d, *J* = 15.8 Hz, 1H), 6.60 (dd, *J* = 9.3, 2.6 Hz, 1H), 6.20 (d, *J* = 2.6 Hz, 1H), 5.71 (d, *J* = 4.4 Hz, 1H), 4.96 – 4.86 (m, 2H), 4.54 (s, 2H), 4.40 – 4.33 (m, 4H), 4.28 (dt, *J* = 12.2, 3.6 Hz, 1H), 4.13 (td, *J* = 5.4, 2.6 Hz, 2H), 3.35 (q, *J* = 7.1 Hz, 4H), 2.92 (t, *J* = 7.1 Hz, 2H), 1.84 – 1.76 (m, 2H), 1.37 (p, *J* = 7.1 Hz, 2H), 1.15 – 1.10 (m, 10H). **HRMS** (ESI): *m/z* calcd. for $C_{37}H_{53}N_{13}O_{16}P_3$ $[M+1H^+]^+$: 1028.2941, found: 1028.2948. *m/z* calcd. for $C_{37}H_{50}N_{13}O_{16}P_3$ $[M-2H^+]^{2-}$: 512.6361, found: 512.6360 (Z=2).

Synthesis of Azido-PAMAM-dendrons, their guanidinium derivatives, and their click reactions.

Synthesis of **Dendrons** was modified from a previously reported procedure (WEI LIU, CHANG-MING DONG. “Versatile Synthesis of Asymmetrical Dendron-Like/Dendron-Like Poly(ε-Caprolactone)-b-Poly(γ-Benzyl-L-Glutamate) Block Copolymers”.[7] *J. Polym. Sci. Part A Polym. Chem.* **2011**, 49, 3491–3498.) and (Jae Wook Lee, Jung Hwan Kim and Byung-Ku Kim “Synthesis of azide-functionalized PAMAM dendrons at the focal point and their application for synthesis of PAMAM-like dendrimers”.[8] Tetrahedron Letters 47, **2006**, 2683–2686).

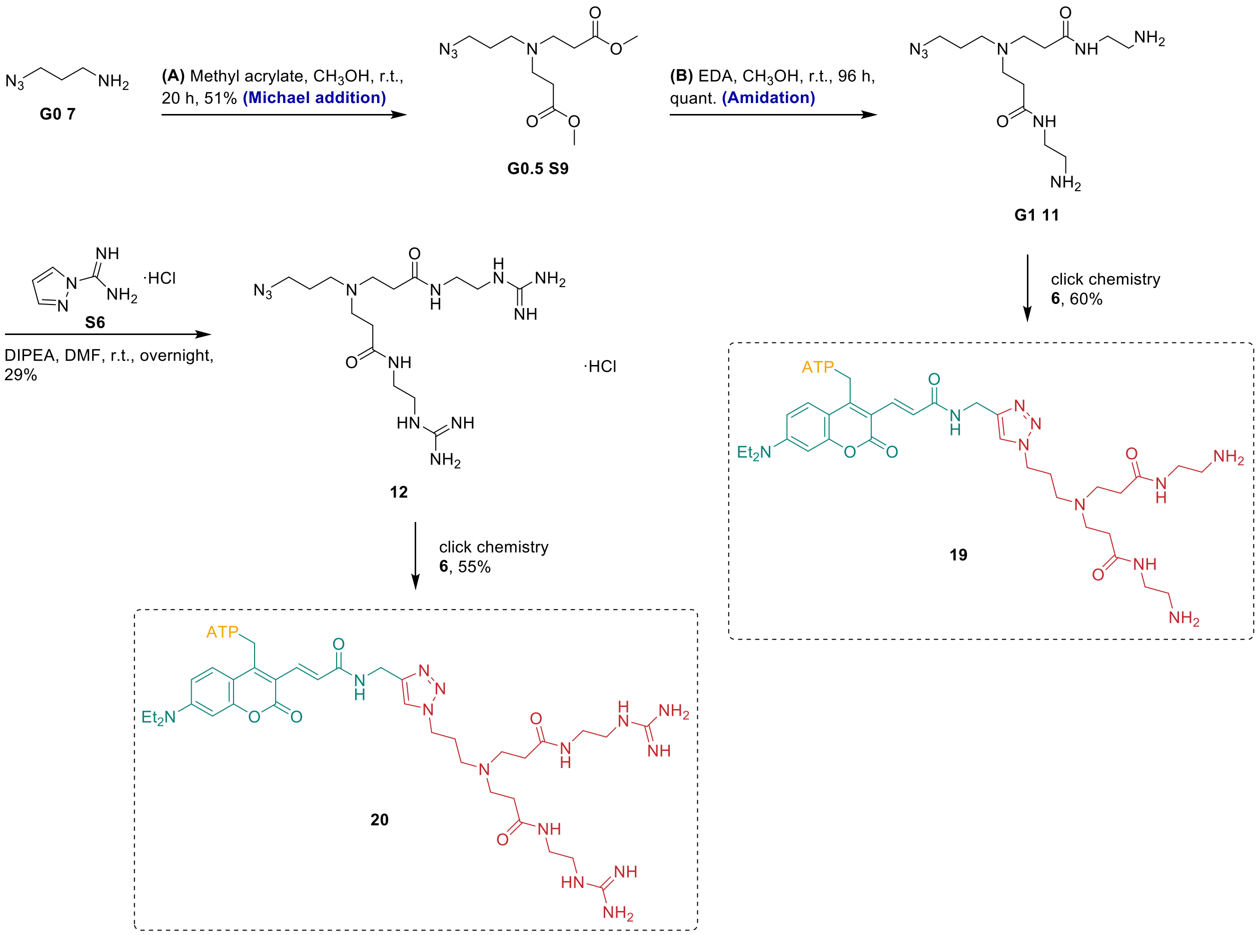

Scheme S5. Synthesis of **G1 11**, its guanidinium detivative **12** and clickable DEAC450 caged ATPs with different transporters attached **19**, **20**.

0.5 G dendron (**S9**)

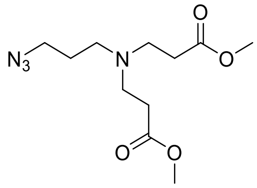

Chemical Formula: $C_{11}H_{20}N_4O_4$
Exact Mass: 272.1485

A solution of methyl acrylate (835 mg, 0.87 ml, 9.7 mmol, 3.5 eq.) in methanol (2 ml) was stirred in round flask at 0 °C (ice-water bath). After 10 min, a solution of **G0 7** (275.9 mg, 2.76 mmol, 1 eq.) in methanol (2 ml) was added dropwise to the cooled solution over 1 h. The reaction mixture was stirred at 0 °C for 1 h and then at r.t for an additional 20 h under Argon atmosphere in the dark. When the reaction finished, solvent was removed under reduced pressure and the residue was purified by silica gel column chromatography (n-hexane/EA, 10:1 to 2:1). Compound **S9** was

obtained (378 mg, 1.4 mmol, 51%) as colorless liquid. (**Note**: Methyl acrylate is light sensitive, so the reaction should run in the dark. **S9** is not UV visible, iodine staining was used.) Rf: 0.3 (n-hexane/EA=2:1). Rotavap temperature: 35 °C. Analytical data is consistent with those reported in the literature. **^{1}H NMR** (400 MHz, $CDCl_3$) δ 3.68 (s, 6H), 3.32 (t, *J* = 6.6 Hz, 2H), 2.79 – 2.71 (m, 4H), 2.48 (t, *J* = 6.6 Hz, 2H), 2.44 (t, *J* = 6.9 Hz, 4H), 1.70 (p, *J* = 6.6 Hz, 2H). **^{13}C NMR** (101 MHz, $CDCl_3$) δ 172.94 (2 C), 51.56 (2 C), 50.55, 49.28 (2 C), 49.17, 32.56 (2 C), 26.68. **HRMS (ESI)**: *m/z* calcd. for $C_{11}H_{21}N_4O_4$, $[M+H^+]^+$: 273.1557, found: 273.1555.

1G dendron (**11**)

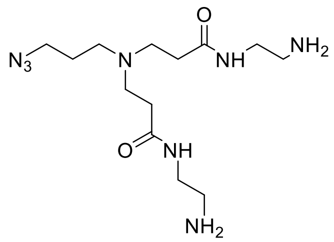

Chemical Formula: $C_{13}H_{28}N_8O_2$
Exact Mass: 328.2335

A solution of ethylenediamine (1.2 g, 1.33 ml, 20 mmol, 20 eq.) in methanol (2 ml) was stirred in round flask at 0 °C (ice-water bath). After 10 min, a solution of **S9** (272.3 mg, 1 mmol, 1 eq.) in methanol (2 ml) was added dropwise to the cooled solution over 1 h. The reaction mixture was stirred at 0 °C for 1 h and then at r.t. for 3 days under Argon atmosphere. When the reaction finished, solvent and excess ethylenediamine was removed under reduced pressure and dried in vacuum. Rotavap temperature: 35 °C. No need further purification. The product can be identified by **^{1}H** NMR spectrum. Compound **11** was obtained (346 mg, ≈ 1 mmol, quant.) **^{1}H NMR** (400 MHz, $CDCl_3$) δ 7.28 (d, *J* = 4.5 Hz, 2H), 3.28 – 3.18 (m, 6H), 2.78 – 2.74 (m, 4H), 2.67 – 2.62 (m, 4H), 2.42 (t, *J* = 6.8 Hz, 2H), 2.30 (m, *J* = 6.7, 5.5 Hz, 4H), 1.69 – 1.61 (m, 2H).

1G dendron (guanidine) (**12**)

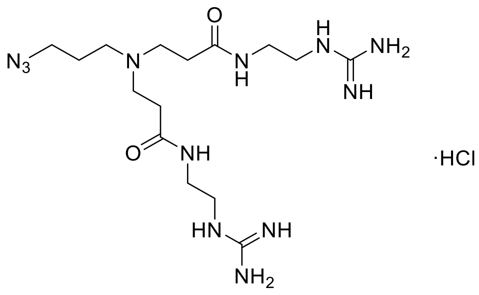

Chemical Formula: $C_{15}H_{32}N_{12}O_2$
Exact Mass: 412.2771

11 (223mg, 0.7 mmol, 1 eq.), 1H-Pyrazole-1-carboxamidine hydrochloride **S6** (440 mg, 3 mmol, 4.3 eq.), DIPEA (426 mg, 0.6 ml, 3.3 mmol, 4.7 eq.) were dissolved in 5 ml of dry DMF and stirred at r.t. overnight. When reaction finished, ether (60 ml) was added to precipitate the crude product. Centrifugation was conducted, ether layer was removed, repeated for three times. Crude product was purified by a MPLC system. It was eluted with increasing concentration of CH_3CN in H_2O from 0-50% using TEAA as buffer. Product **12** (162 mg, 29%) was obtained as colorless oil. **^{1}H NMR** (300 MHz, Methanol-d_4) δ 3.41 – 3.30 (m, 10H), 2.85 (t, J = 6.7 Hz, 4H), 2.63 (t, J = 7.0 Hz, 2H), 2.44 (t, J = 6.7 Hz, 4H), 1.84 – 1.71 (m, 2H). **HRMS** (ESI): *m/z* calcd. for $C_{15}H_{33}N_{12}O_2$ $[M+H^+]^+$: 413.2844, found: 413.2844.

(G1-NH_2-) (**19)**

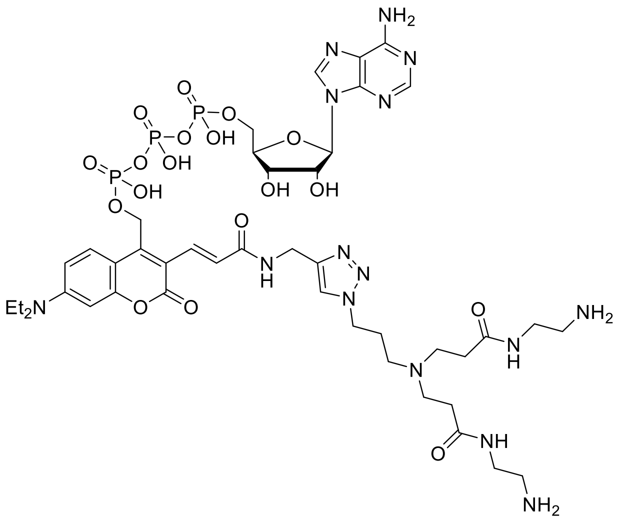

Chemical Formula: $C_{43}H_{64}N_{15}O_{18}P_3$
Exact Mass: 1171.3767

19 was synthesized by click reaction between caged ATP **6** and compound **11**. Caged ATP **6** (20 mg, 17 μmol, 1 eq.) was used for the reaction. Compound **19** (12 mg, 60%) was obtained as orange solid. **^{31}P NMR** (162 MHz, D_2O) δ -11.37 (d, J = 19.0 Hz), -11.94 (d, J = 19.2 Hz), -23.05. **^{1}H**

NMR (400 MHz, D_2O) δ 8.17 (s, 1H), 7.97 – 7.95 (m, 2H), 7.52 – 7.44 (m, 2H), 6.81 (d, J = 15.8 Hz, 1H), 6.65 (dd, J = 9.4, 2.6 Hz, 1H), 6.27 (d, J = 2.5 Hz, 1H), 5.71 (d, J = 4.4 Hz, 1H), 4.98 – 4.88 (m, 2H), 4.56 (s, 2H), 4.43 – 4.33 (m, 4H), 4.30 (dt, J = 12.1, 3.6 Hz, 1H), 4.14 (t, J = 4.8 Hz, 2H), 3.39 (q, J = 7.1 Hz, 4H), 3.11 (t, J = 6.3 Hz, 4H), 2.65 (dt, J = 18.4, 6.7 Hz, 10H), 2.37 – 2.30 (m, 2H), 2.26 (t, J = 7.3 Hz, 4H), 2.01 (t, J = 7.7 Hz, 2H), 1.15 (t, J = 7.1 Hz, 6H). **HRMS** (ESI): *m/z* calcd. for $C_{43}H_{65}N_{15}O_{18}P_3$ $[M+H^+]^+$: 1172.3839, found: 1172.3833; *m/z* calcd. for $C_{43}H_{63}N_{15}O_{18}P_3$ $[M-H^+]^-$: 1170.3694, found: 1170.3691.

(G1-guanidine-) (**20)**

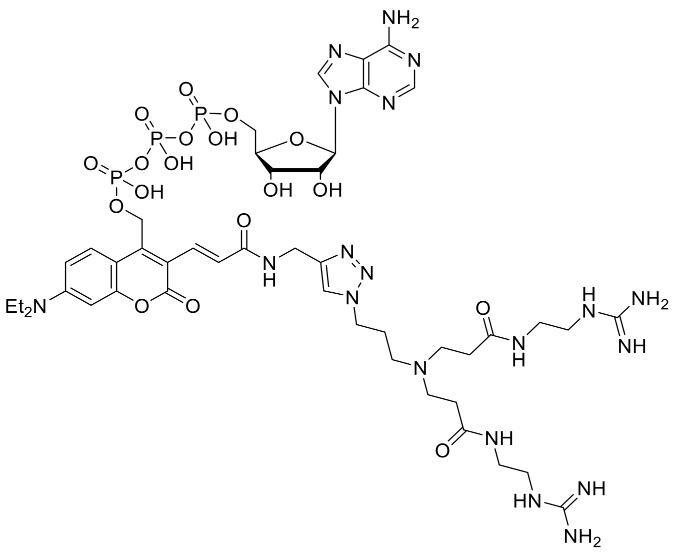

Chemical Formula: $C_{45}H_{68}N_{19}O_{18}P_3$
Exact Mass: 1255.4203

20 was synthesized by click reaction between caged ATP **6** and compound **12**. Caged ATP **6** (20 mg, 17 μmol, 1 eq.) was used for the reaction. Compound **20** (11 mg, 55%) was obtained as orange solid. **^{31}P NMR** (162 MHz, D_2O) δ -11.28 (d, *J* = 19.4 Hz), -11.94 (d, *J* = 18.5 Hz), -22.87 (t, *J* = 19.2 Hz). **^{1}H NMR** (400 MHz, D_2O) δ 8.19 (d, *J* = 4.6 Hz, 1H), 7.92 (d, *J* = 8.0 Hz, 2H), 7.48 – 7.34 (m, 2H), 6.88 (d, *J* = 15.7 Hz, 1H), 6.59 (d, *J* = 9.5 Hz, 1H), 6.21 (s, 1H), 5.74 (d, *J* = 4.9 Hz, 1H), 5.04 – 4.87 (m, 2H), 4.59 – 4.49 (m, 2H), 4.48 – 4.31 (m, 5H), 4.20 (d, *J* = 15.8 Hz, 2H), 3.37 (d, *J* = 7.5 Hz, 4H), 3.09 (d, *J* = 8.0 Hz, 8H), 2.52 (d, *J* = 10.6 Hz, 4H), 2.24 – 2.07 (m, 6H), 1.96 (s, 2H), 1.14 (t, *J* = 7.0 Hz, 6H). **HRMS** (ESI): *m/z* calcd. for $C_{45}H_{67}N_{19}O_{18}P_3$ $[M-1H^+]^-$: 1254.4130, found: 1254.4122; *m/z* calcd. for $C_{45}H_{69}N_{19}O_{18}P_3$ $[M+1H^+]^+$: 1256.4275, found: 1256.4282.

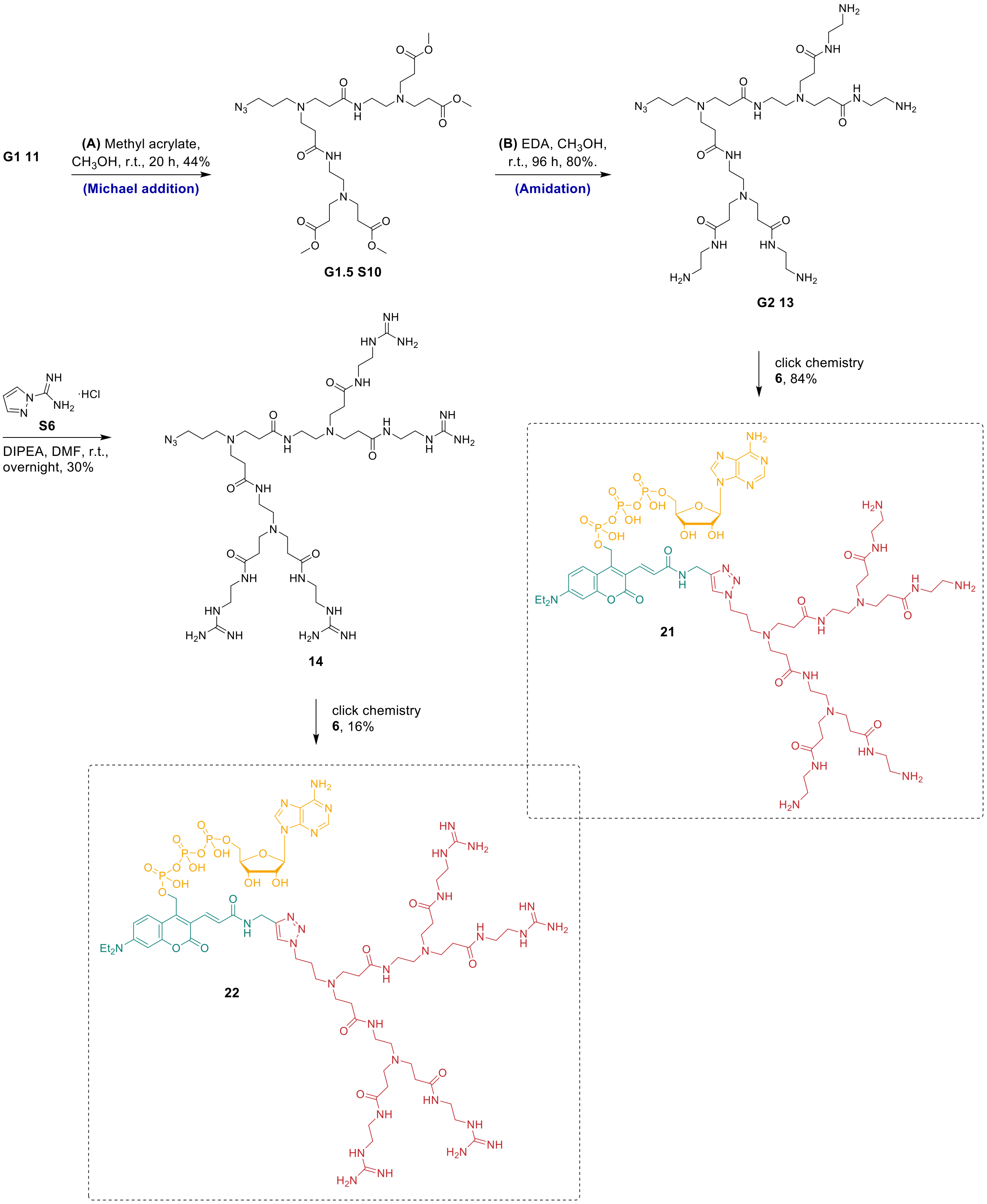

Scheme S6. Synthesis of **G2 13**, its guanidinium derivative **14** and clickable DEAC450 caged ATPs with different transporters attached **21**, **22**.

1.5 G dendron (**S10**)

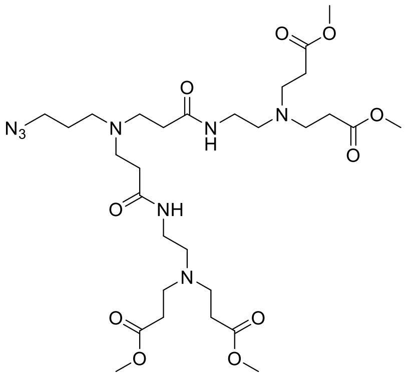

Chemical Formula: $C_{29}H_{52}N_8O_{10}$
Exact Mass: 672.3806

A solution of methyl acrylate (4.13 g, 4.32 ml, 48 mmol, 8 eq.) in methanol (15 ml) was stirred in round flask at 0 °C (ice-water bath). After 10 min, a solution of **G1 11** (1.97 g, 6 mmol, 1 eq.) in methanol (20 ml) was added dropwise to the cooled solution over 1 h. The reaction mixture was stirred at 0 °C for 1 h and then at r.t. for an additional 20 h under Ar atmosphere in the dark. When the reaction finished, solvent was removed under reduced pressure and the residue was purified by a PF-30SIHP-F0120 (30 μm) column chromatography using a MPLC system. It was eluted with increasing concentration of CH_3OH in DCM from 0-25%. Product came at around 10% CH_3OH in DCM. Product **S10** (1789 mg, 44%) was obtained as colorless oil. **Note:** The product is not visible at 254 nm and 365 nm, but it is detectable by UV at 200 nm and ELSD. TLC monitor and iodine staining are possible. Rf: 0.1 (DCM/CH_3OH = 10/1). Rotavap temperature: 35 °C. Analytical data is consistent with those reported in the literature. **^{1}H NMR** (300 MHz, $CDCl_3$) δ 6.96 (t, *J* = 5.2 Hz, 2H), 3.70 (s, 12H), 3.32 (dt, *J* = 9.4, 6.1 Hz, 6H), 2.83 – 2.75 (m, 12H), 2.55 (q, *J* = 7.1, 6.4 Hz, 6H), 2.46 (t, *J* = 6.7 Hz, 8H), 2.38 (t, *J* = 6.7 Hz, 4H), 1.75 (p, *J* = 6.7 Hz, 2H). **HRMS (ESI)**: *m/z* calcd. for $C_{29}H_{53}N_8O_{10}$ $[M+H^+]^+$: 673.3885, found: 673.3886.

2G dendron (**13**)

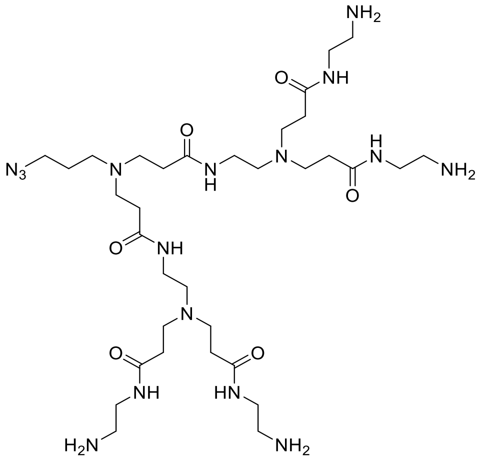

Chemical Formula: $C_{33}H_{68}N_{16}O_6$
Exact Mass: 784.5508

A solution of ethylenediamine (360.6 mg, 0.4 ml, 6 mmol, 40 eq.) in methanol (2 ml) was stirred in round flask at 0 °C (ice-water bath). After 10 min, a solution of **S10** (100 mg, 0.15 mmol, 1 eq.) in methanol (2 ml) was added dropwise to the cooled solution over 20 min. The reaction mixture was stirred at 0 °C for 1 h and then at r.t for 10 days under Ar atmosphere. When the reaction finished, solvent and excess ethylenediamine was removed under reduced pressure and dried in vacuum. Rotavap temperature: 35 °C. The product was identified by **^{1}H** NMR spectrum. Compound **13** was obtained (92 mg, 0.12 mmol, 80%) as light-yellow oil. This reaction was monitored by mass. The mass was taken after 3 days, 7 days, and 10 days. In the end, **13** was the main product. **HRMS (ESI)**: *m/z* calcd. for $C_{33}H_{69}N_{16}O_6$ $[M+H^+]^+$: 785.5581, found: 785.5579.

2G dendron (guanidine) (**14**)

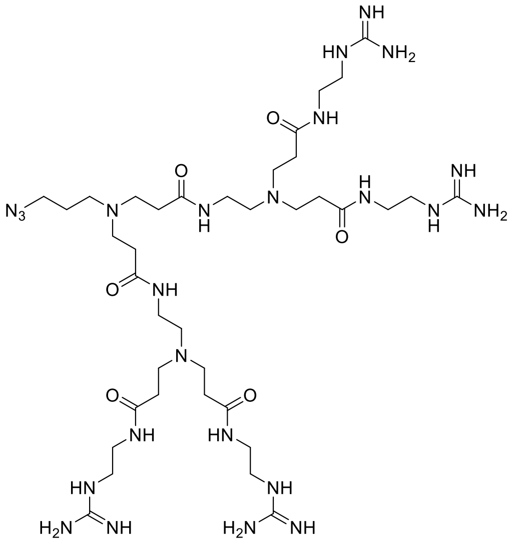

Chemical Formula: $C_{37}H_{76}N_{24}O_6$
Exact Mass: 952.6380

13 (200 mg, 255 μmol, 1 eq.), 1H-Pyrazole-1-carboxamidine hydrochloride **S6** (224 mg, 1.5 mmol, 6 eq.), DIPEA (194 mg, 261 μl, 1.5 mmol, 6 eq.) were dissolved in 3 ml of dry DMF and stirred at room temperature overnight. When reaction finished, ether (20 ml) was added to precipitate the crude product. Centrifugation was conducted, ether layer was removed, repeated for three times. Crude product was purified by a MPLC system. It was eluted with increasing concentration of CH_3CN in H_2O from 0-50% using TEAA as buffer. Product **14** (84 mg, 30%) was obtained as colorless oil. **HRMS** (ESI): *m/z* calcd. for $C_{37}H_{77}N_{24}O_6$ $[M+H^+]^+$: 953.6452, found: 953.6472. **HRMS** (ESI): *m/z* calcd. for $C_{37}H_{76}N_{24}O_6Na$ $[M+Na^+]^+$: 975.6272, found: 975.6286.

(G2-NH$_2$-) (**21**)

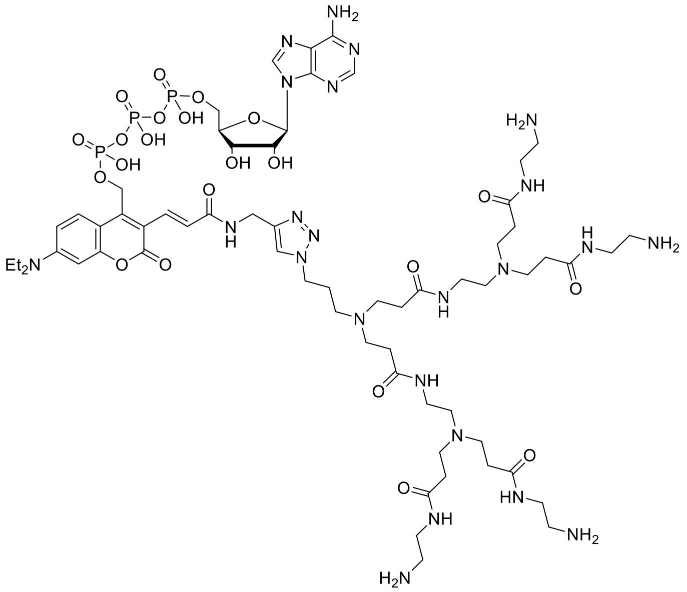

Chemical Formula: $C_{63}H_{104}N_{23}O_{22}P_3$
Exact Mass: 1627.6939

21 was synthesized by click reaction between caged ATP **6** and compound **13**. Caged ATP **6** (40 mg, 34 µmol, 1 eq.), copper(II) sulfate (4.6 mg, 17 µmol, 0.5 eq.), sodium L-ascorbate (6.8 mg, 34 µmol, 1.0 eq.) were dissolved in 2 ml of H_2O in a flask. Afterwards **13** (116 mg, 147 µmol, 4 eq.) was dissolved in 2 ml of CH_3OH and added to the flask. The reaction mixture was stirred at room temperature and monitored by HPLC-UV. After 4 h, reaction finished. When the reaction finished, CH_3OH was removed under vacuum. The resulting solution was purified by a PF-C18AQ-F0040 (30 µm) column chromatography using a MPLC system (ELSD detection, no UV detection). It was eluted with increasing concentration of CH_3CN in H_2O from 0-50% using TEAA as buffer. Products came at around 25% CH_3CN in H_2O. The product containing fractions were combined and lyophilized. Copper was removed by Chelex® 100 sodium form (5 mg Chelex® 100 was added to sample solution, stirred for 5 min, repeated for three times.). TEA^+ was also removed by Chelex® 100. Compound **21** (47 mg, 84%) was obtained as orange solid. **^{1}H NMR** (400 MHz, D_2O) δ 8.15 (d, *J* = 5.2 Hz, 1H), 7.93 (dd, *J* = 13.8, 2.9 Hz, 2H), 7.43 (dd, *J* = 15.5, 5.8 Hz, 1H), 7.34 (d, *J* = 9.3 Hz, 1H), 6.75 (d, *J* = 15.4 Hz, 1H), 6.52 (d, *J* = 9.3 Hz, 1H), 6.19 (d, *J* = 2.4 Hz, 1H), 5.70 (t, *J* = 4.1 Hz, 1H), 4.96 – 4.93 (m, 2H), 4.55 – 4.50 (m, 2H), 4.40 – 4.31 (m, 4H), 4.25 – 4.09 (m, 3H), 3.36 – 3.24 (m, 12H), 3.06 (t, *J* = 7.6 Hz, 4H), 2.92 (t, *J* = 6.0 Hz, 6H), 2.75 (dd, *J* = 10.9, 5.4 Hz, 4H), 2.60 (t, *J* = 7.3 Hz, 10H), 2.40 (t, *J* = 7.2 Hz, 6H), 2.28 – 2.17 (m, 12H), 1.99 (s, 2H), 1.76 – 1.67 (m, 2H), 1.10 (t, *J* = 6.9 Hz, 6H). **^{31}P NMR** (162 MHz, D_2O) δ -11.10 (d, *J* = 16.1 Hz), -

11.79 (d, *J* = 16.3 Hz), -21.40 – -21.86 (m). **HRMS** (ESI): *m/z* calcd. for $C_{63}H_{103}N_{23}O_{22}P_3$ [M-1H$^+$]$^-$: 1626.6866, found: 1626.6854; *m/z* calcd. for $C_{63}H_{106}N_{23}O_{22}P_3$ [M+2H$^+$]$^{2+}$: 814.8542, found: 814.8541 (Z=2).

(G2-guanidine-) (**22**)

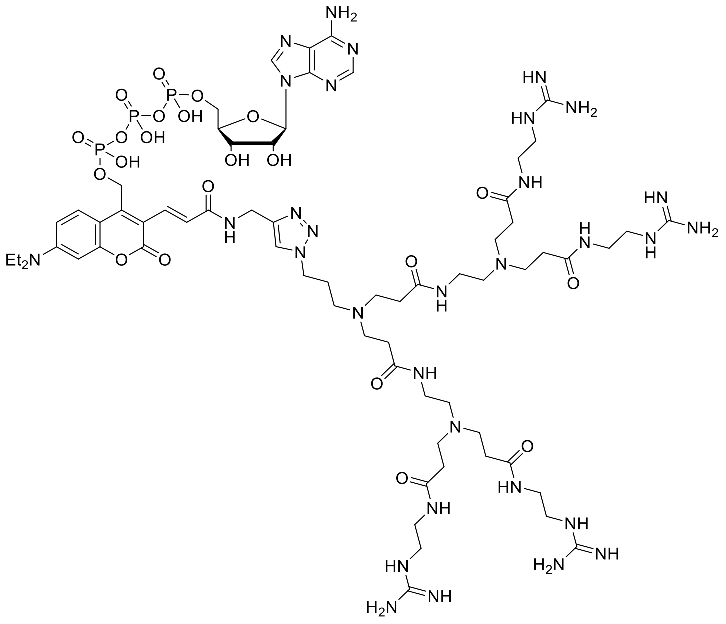

Chemical Formula: $C_{67}H_{112}N_{31}O_{22}P_3$
Exact Mass: 1795.7811

22 was synthesized by click reaction between caged ATP **6** and compound **14**. Caged ATP **6** (20 mg, 17 μmol, 1 eq.) was used for the reaction. **22** (6 mg, 16%) was obtained as orange solid. **^{31}P NMR** (162 MHz, D_2O) δ -11.16 (d, *J* = 16.9 Hz), -11.80 (d, *J* = 17.2 Hz), -22.00. **HRMS** (ESI): *m/z* calcd. for $C_{67}H_{114}N_{31}O_{22}P_3$ [M+2H$^+$]$^{2+}$: 898.8978 (Z=2), found: 898.8983 (Z=2); *m/z* calcd. for $C_{67}H_{115}N_{31}O_{22}P_3$ [M+3H$^+$]$^{3+}$: 599.6010 (Z=3), found: 599.6012 (Z=3).

7. References

[1] T. Weinrich, M. Gränz, C. Grünewald, T. F. Prisner, M. W. Göbel, *European J. Org. Chem.* **2017**, *2017*, 491–496.

[2] J. Ma, A. Ripp, D. Wassy, T. Dürr, D. Qiu, M. Häner, T. Haas, C. Popp, D. Bezold, S. Richert, B. Esser, H. J. Jessen, *Molecules* **2020**, *25*, 5325.

[3] J. P. Olson, H. B. Kwon, K. T. Takasaki, C. Q. Chiu, M. J. Higley, B. L. Sabatini, G. C. R. Ellis-Davies, *J. Am. Chem. Soc.* **2013**, *135*, 5954–5957.

[4] A. Hofer, G. S. Cremosnik, A. C. Müller, R. Giambruno, C. Trefzer, G. Superti-Furga, K. L. Bennett, H. J. Jessen, *Chem. - A Eur. J.* **2015**, *21*, 10116–10122.

[5] R. Zhang, N. Zheng, Z. Song, L. Yin, J. Cheng, *Biomaterials* **2014**, *35*, 3443–3454.

[6] C. Romuald, E. Busseron, F. Coutrot, *J. Org. Chem.* **2010**, *75*, 6516–6531.

[7] C.-M. D. WEI LIU, *J. Polym. Sci. Part A Polym. Chem.* **2011**, *49*, 3491–3498.

[8] J. W. Lee, J. H. Kim, B. K. Kim, *Tetrahedron Lett.* **2006**, *47*, 2683–2686.

8. NMR-spectra and HRMS data

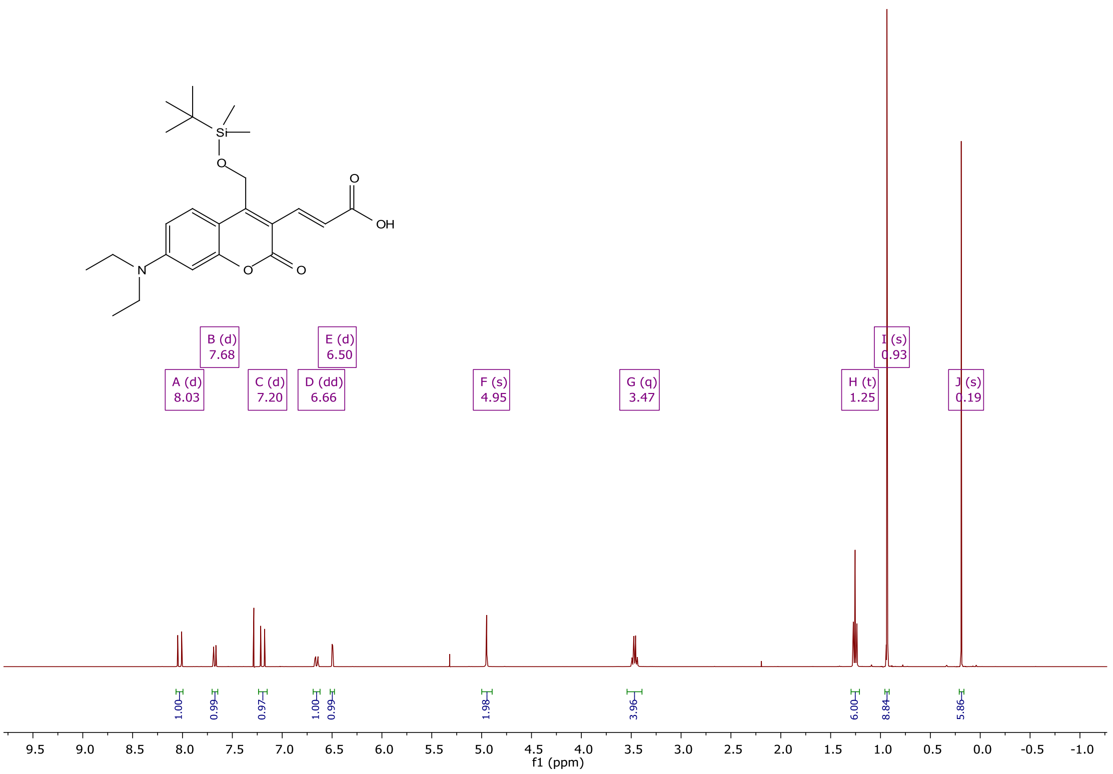

Figure 8.1. ^{1}**H** NMR spectrum of **1**.

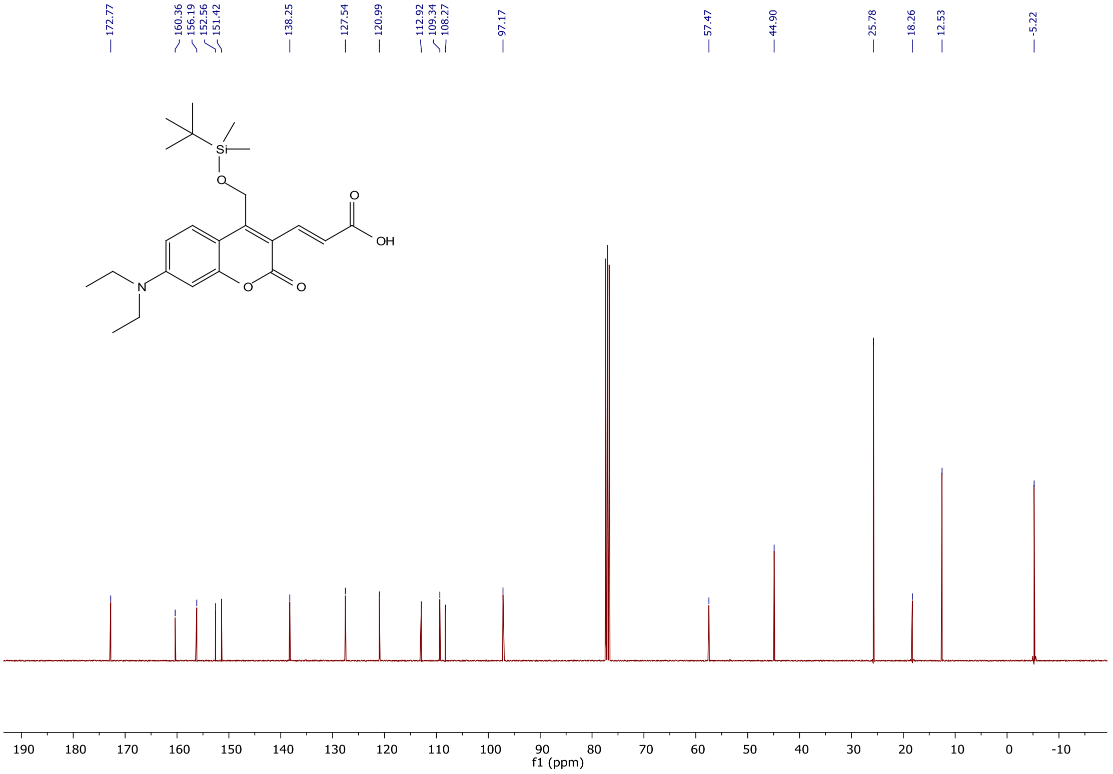

Figure 8.2. ^{13}C NMR spectrum of **1**.

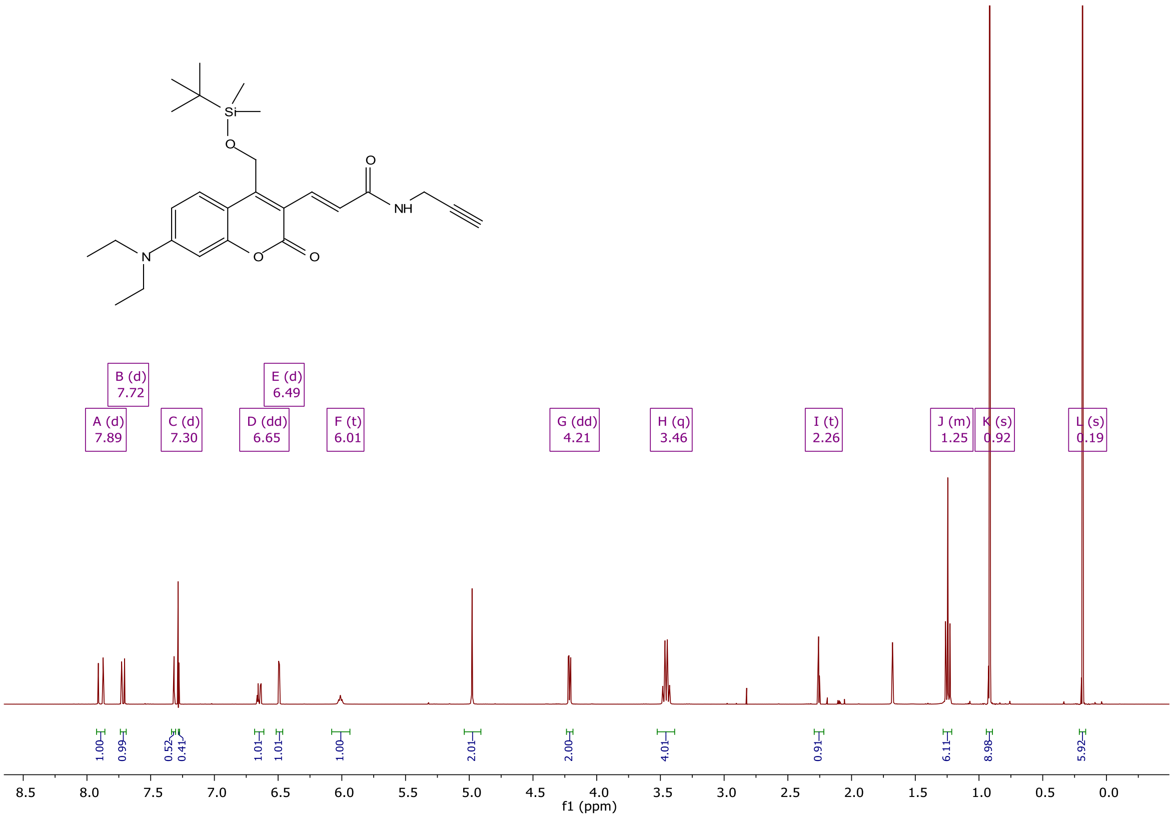

Figure 8.3. [1]**H** NMR spectrum of **2**.

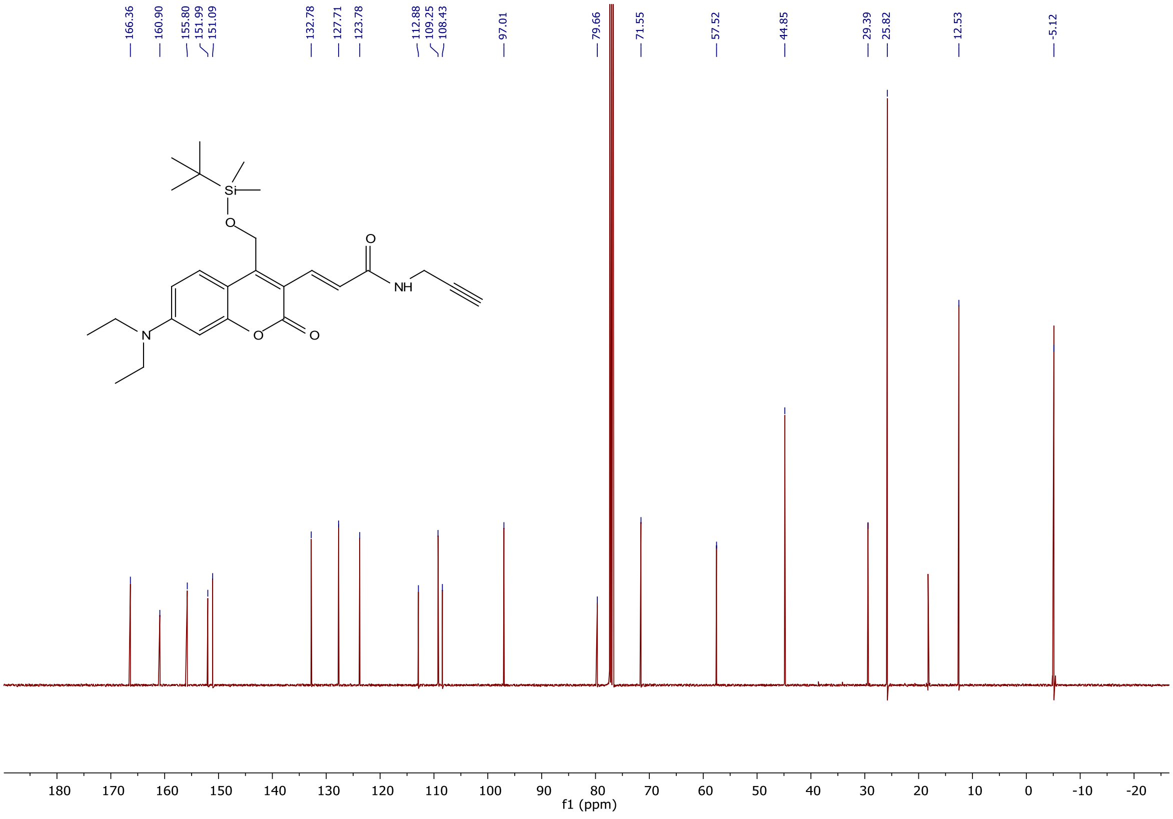

Figure 8.4. [13]**C** NMR spectrum of **2**.

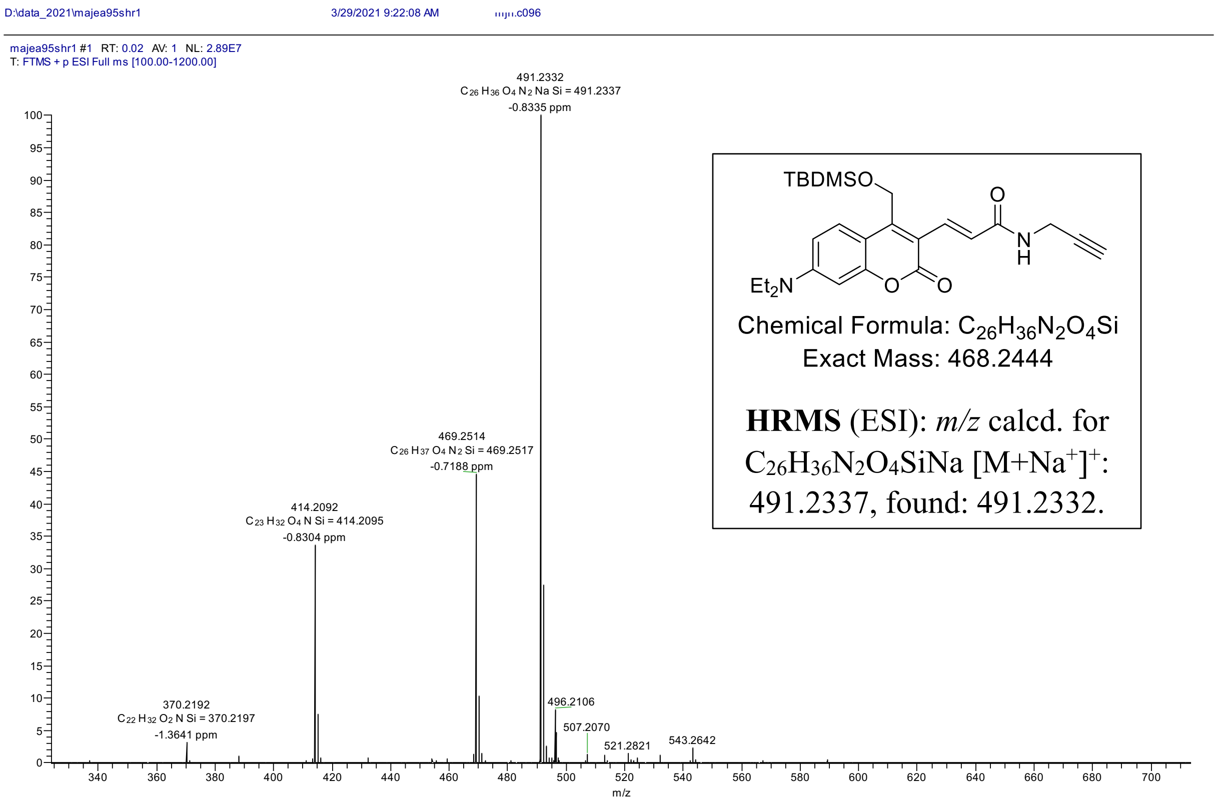

Figure 8.5. MASS spectrum of **2**.

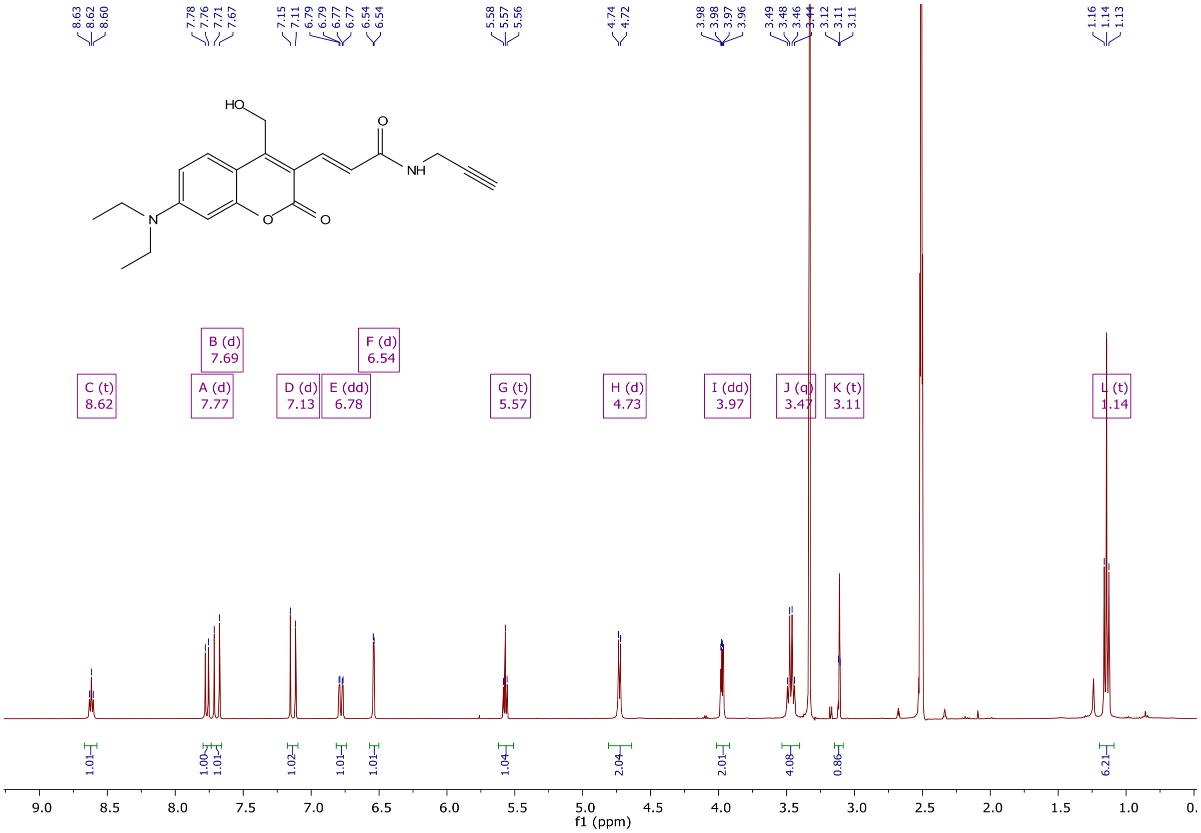

Figure 8.6. ^{1}**H** NMR spectrum of **3**.

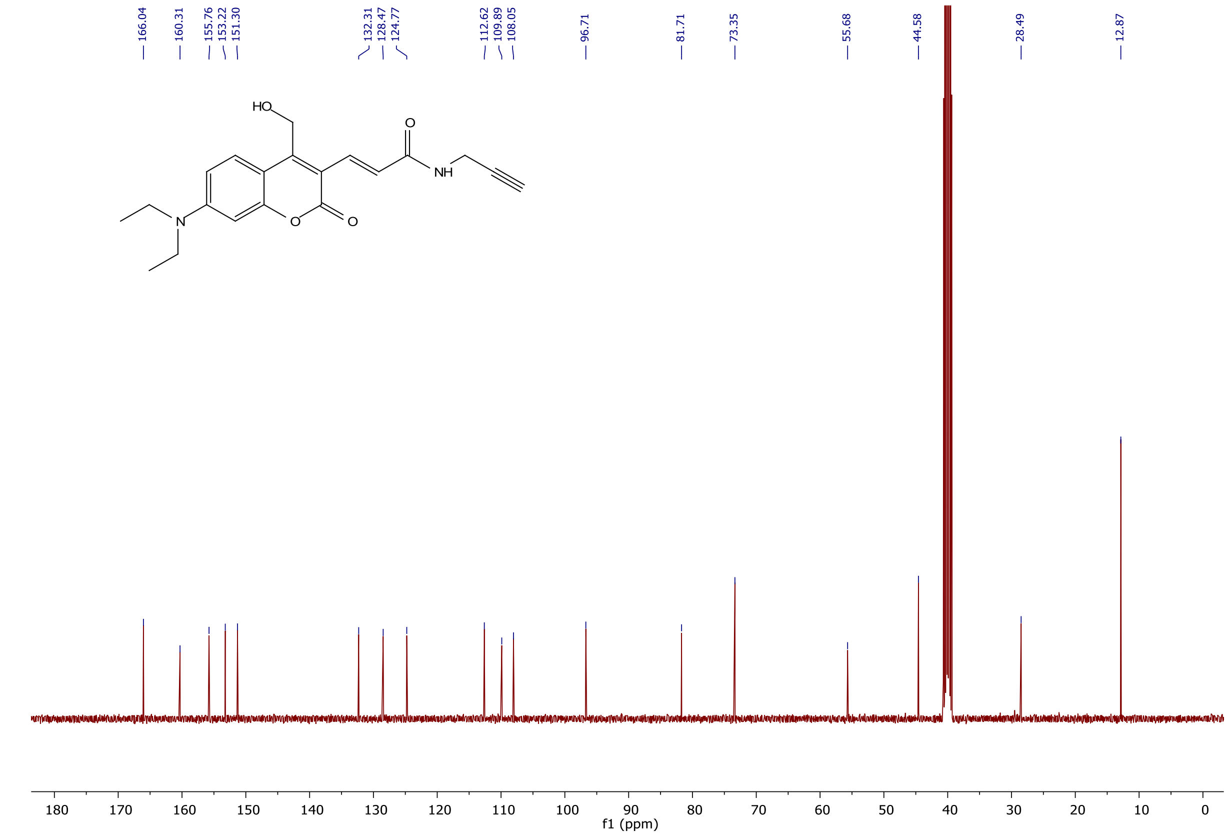

Figure 8.7. ^{13}C NMR spectrum of **3**.

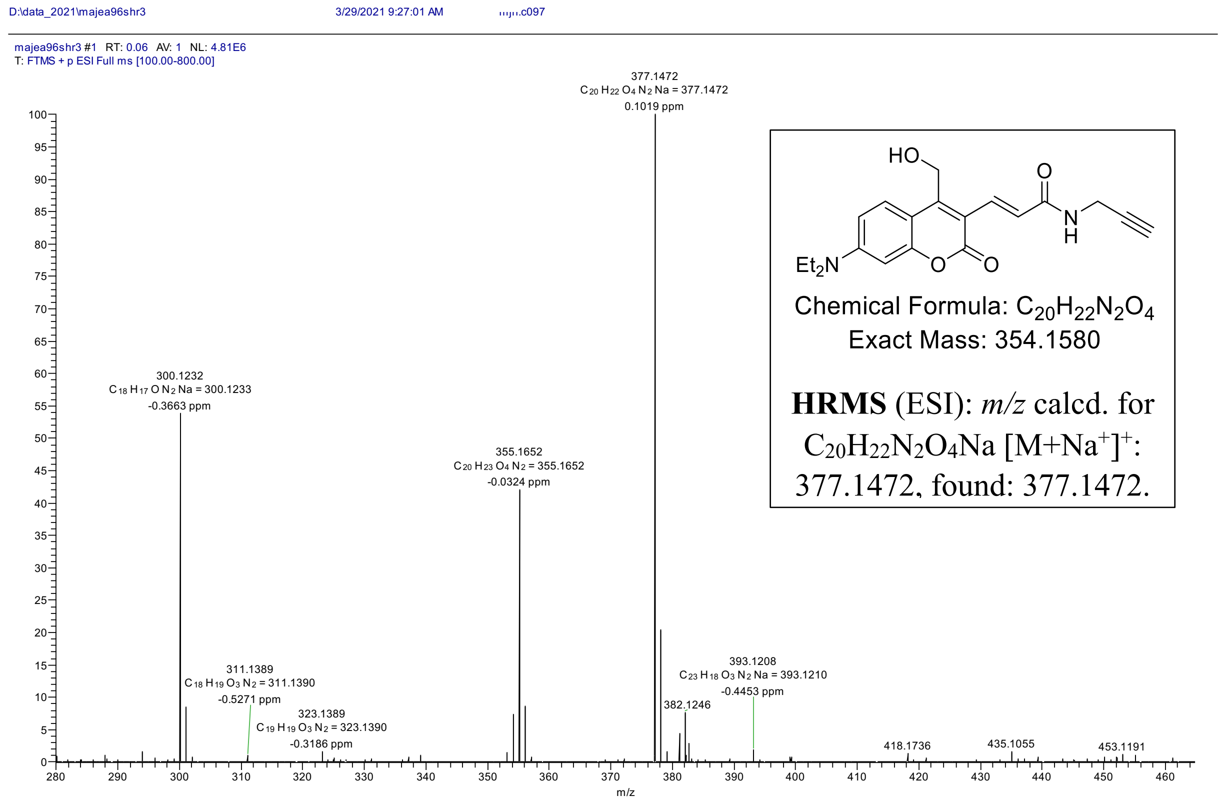

Figure 8.8. MASS spectrum of **3**.

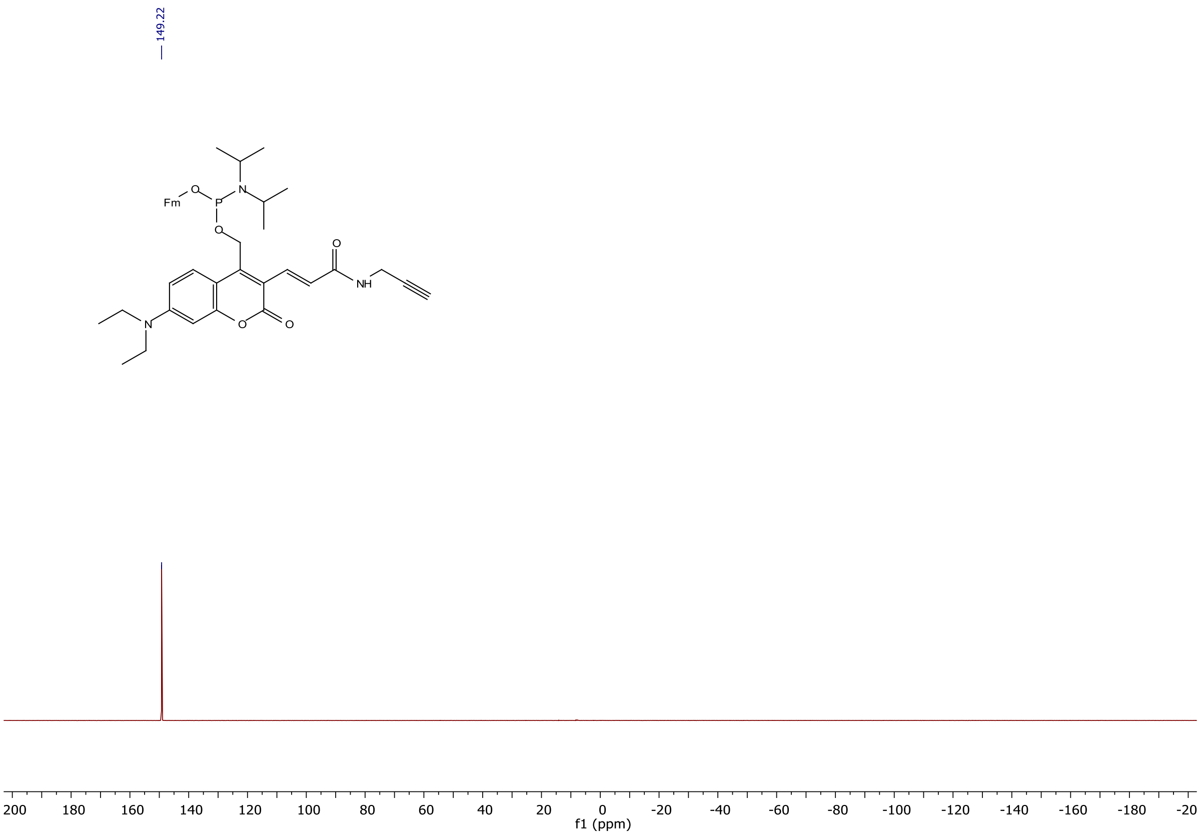

Figure 8.9. ^{31}**P** NMR spectrum of **5**.

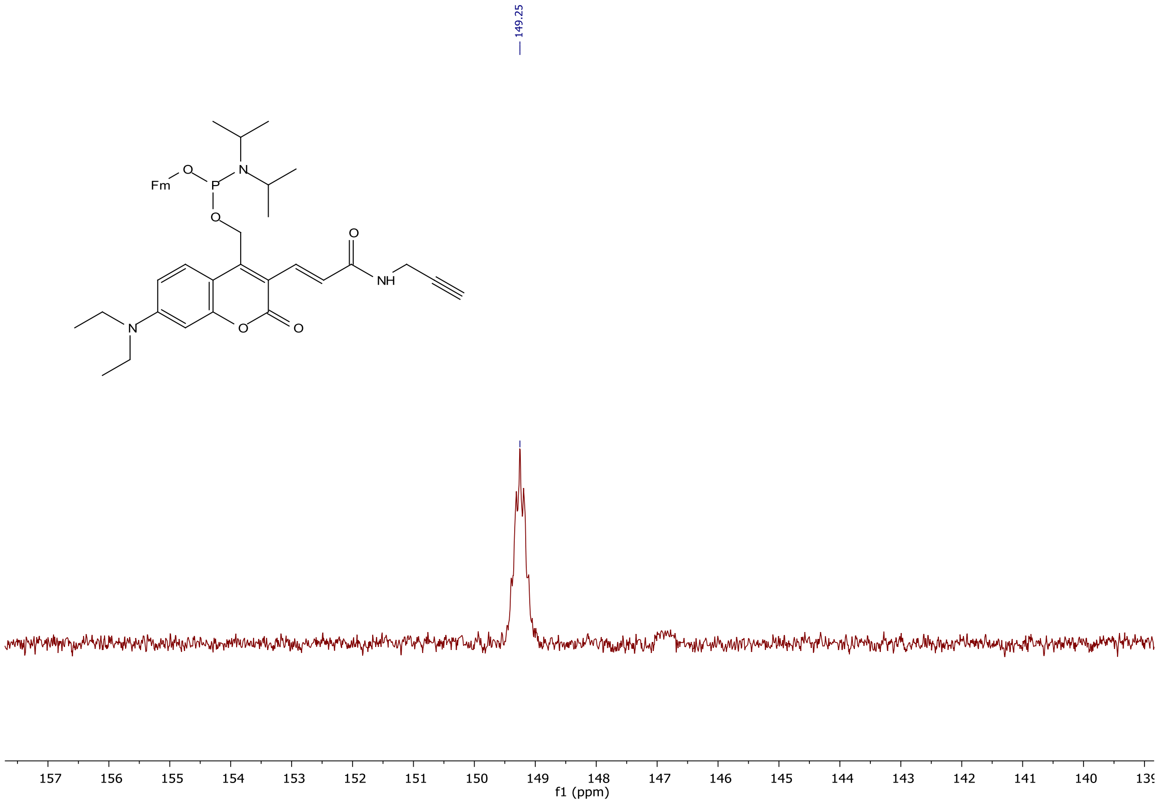

Figure 8.10. ^{31}**P coupled** NMR spectrum of **5**.

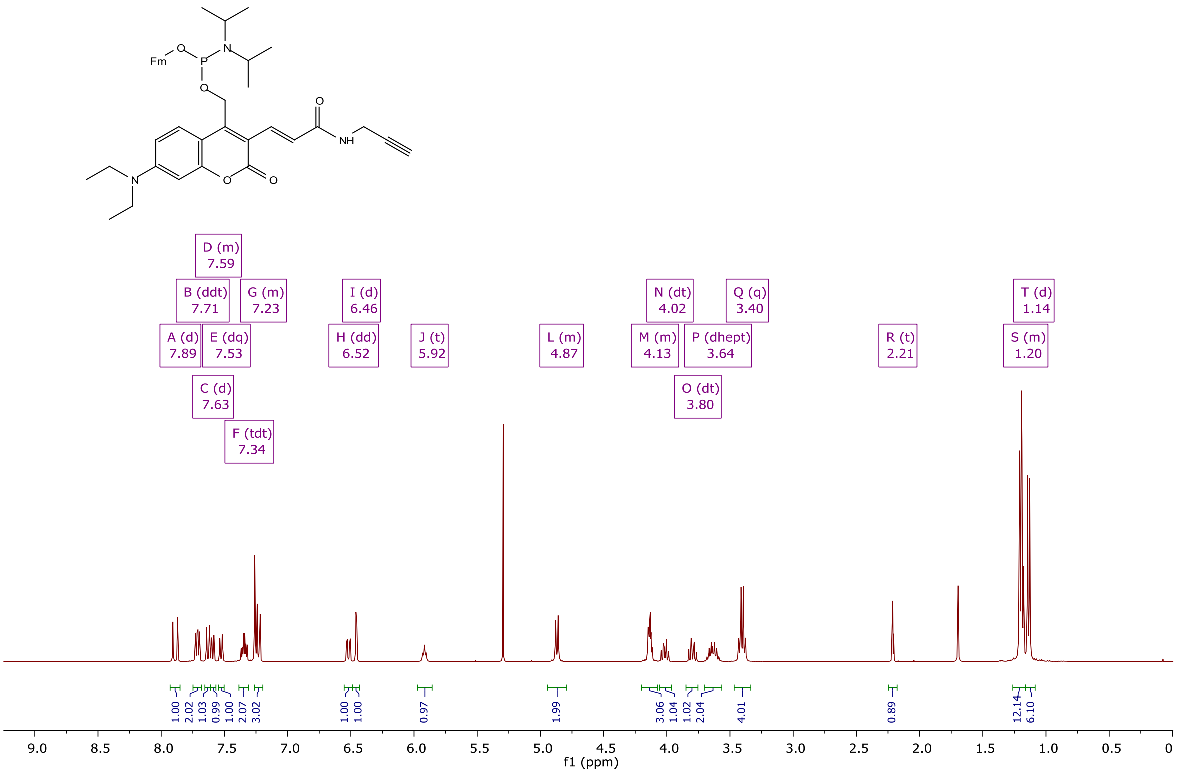

Figure 8.11. ^{1}H NMR spectrum of **5**.

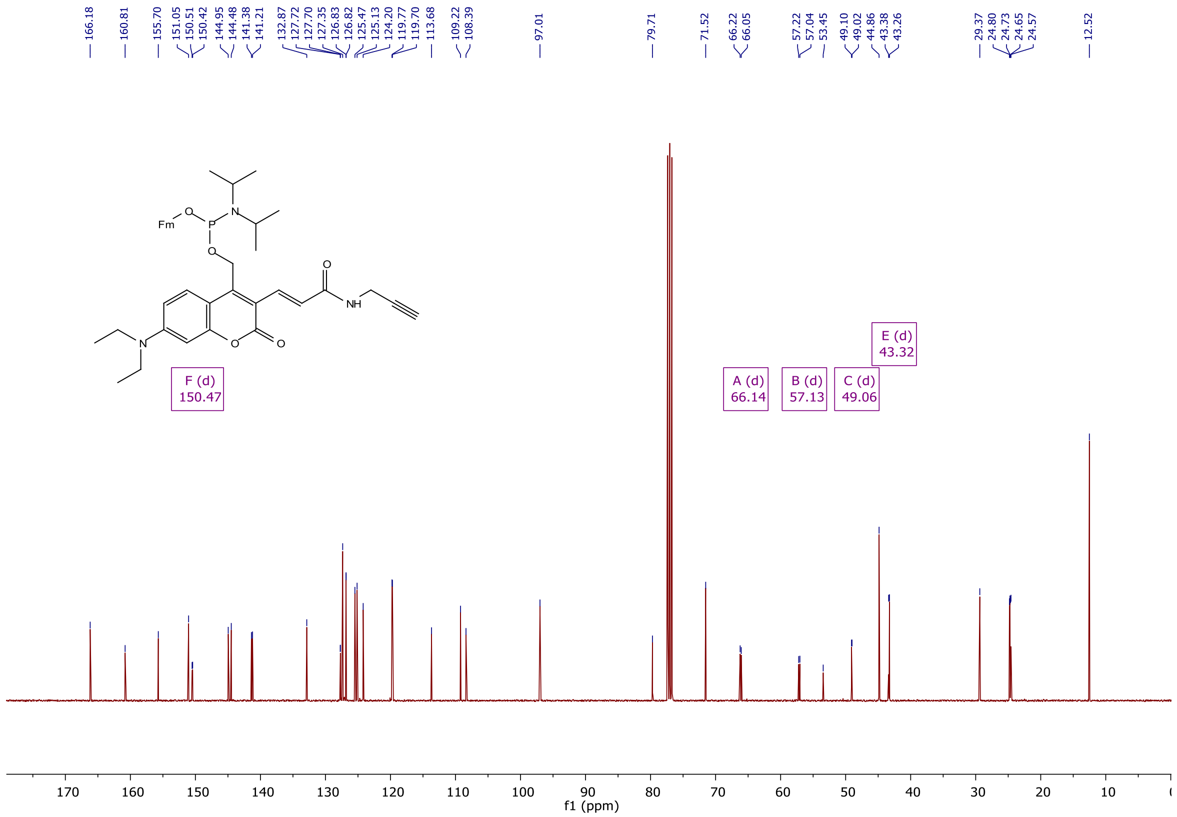

Figure 8.12. ^{13}C NMR spectrum of **5**.

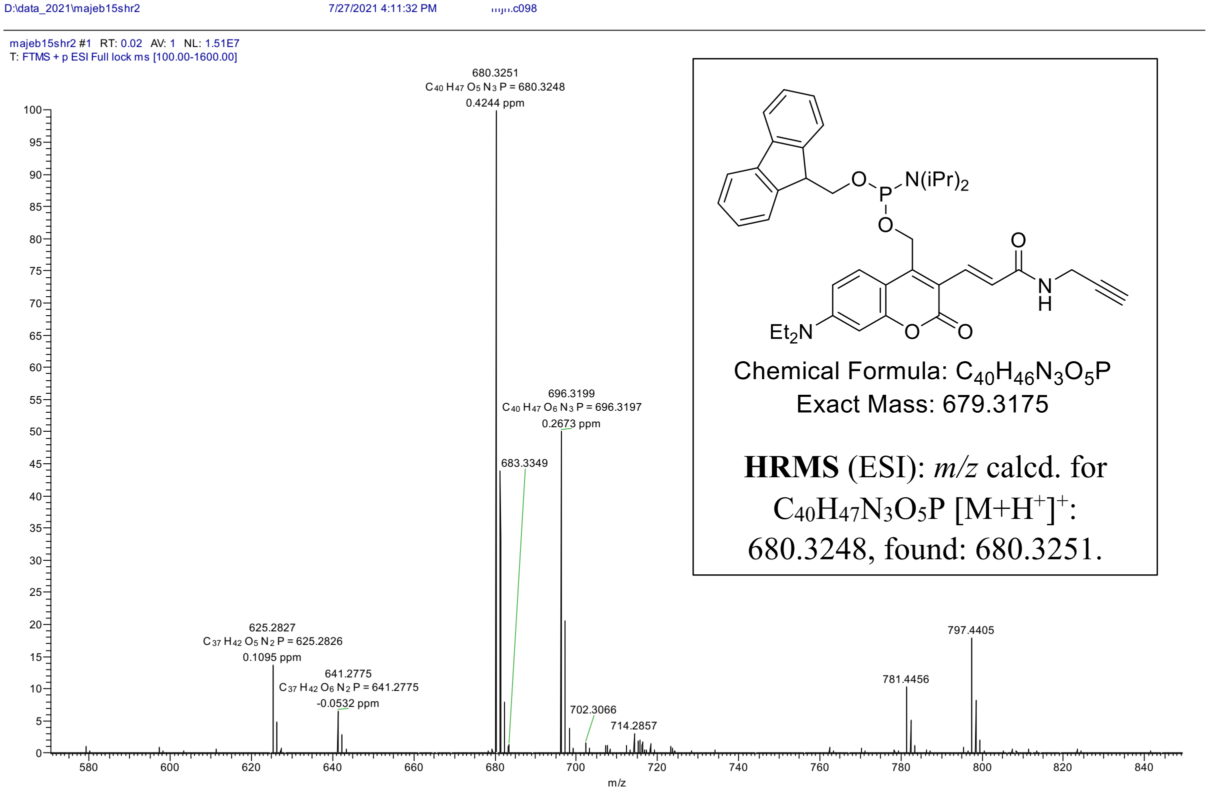

Figure 8.13. MASS spectrum of **5**.

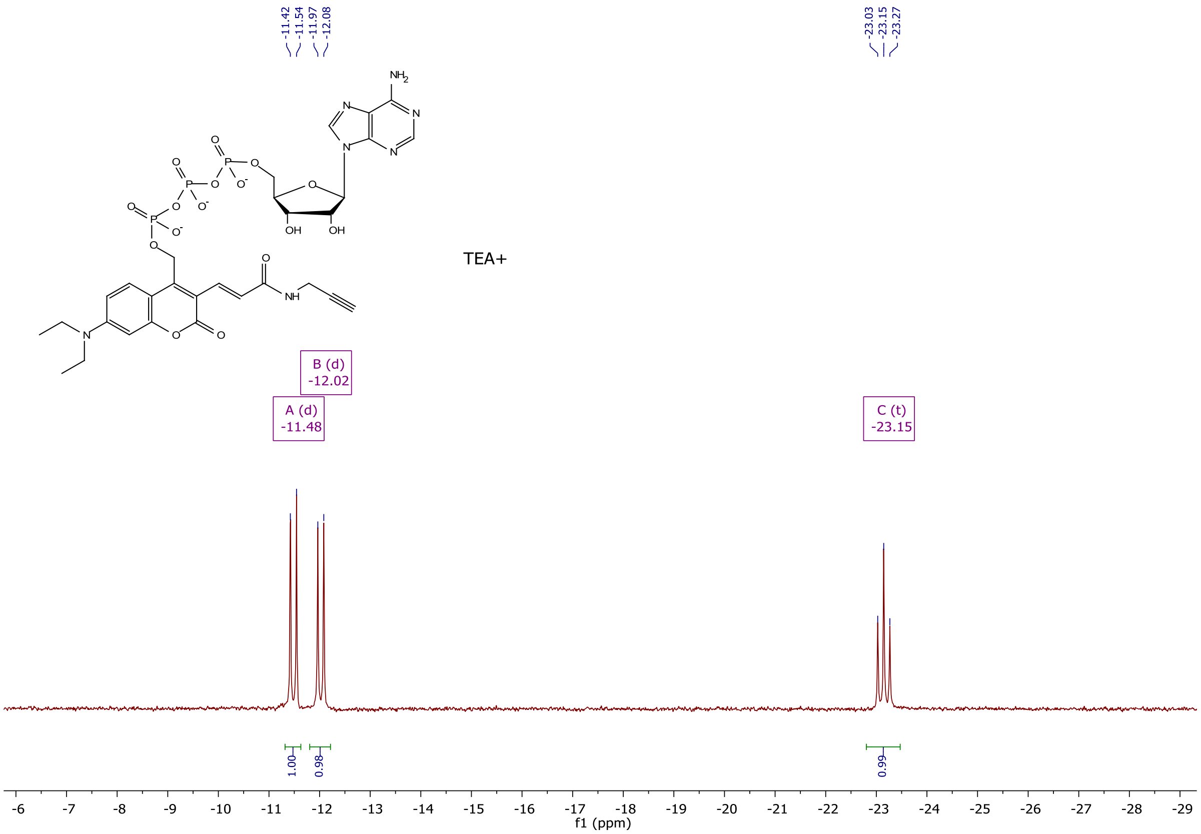

Figure 8.14. ^{31}P NMR spectrum of **6**.

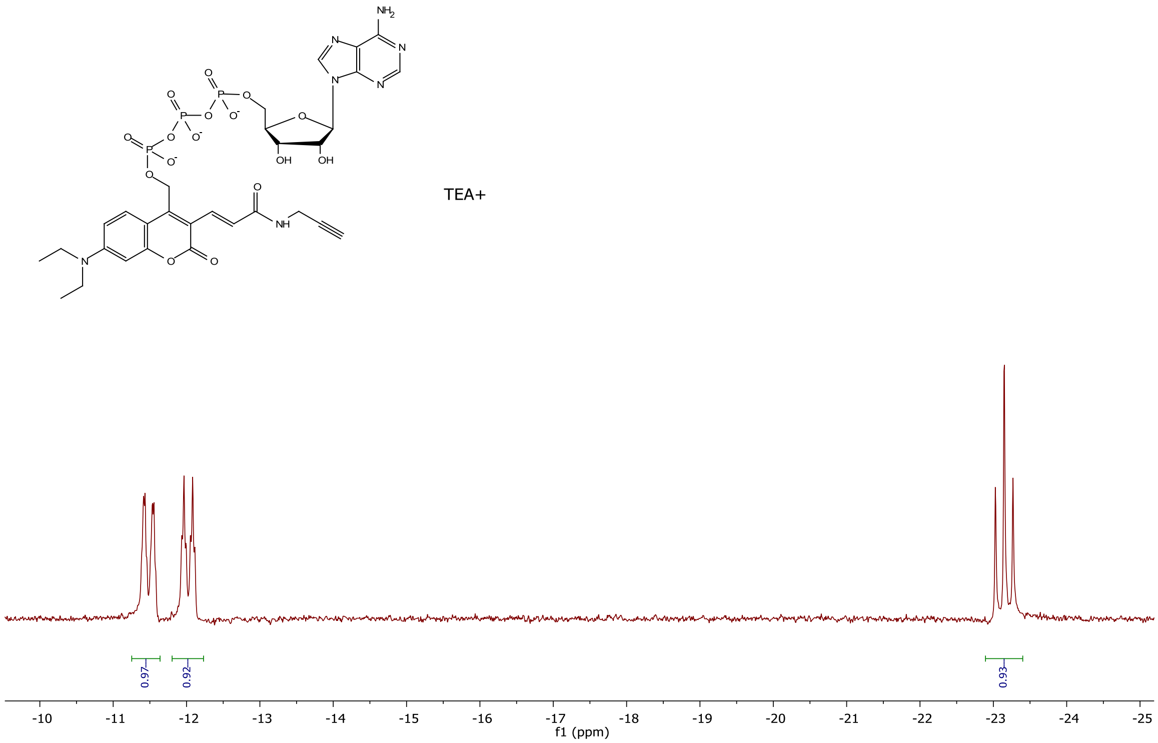

Figure 8.15. ^{31}P coupled NMR spectrum of **6**.

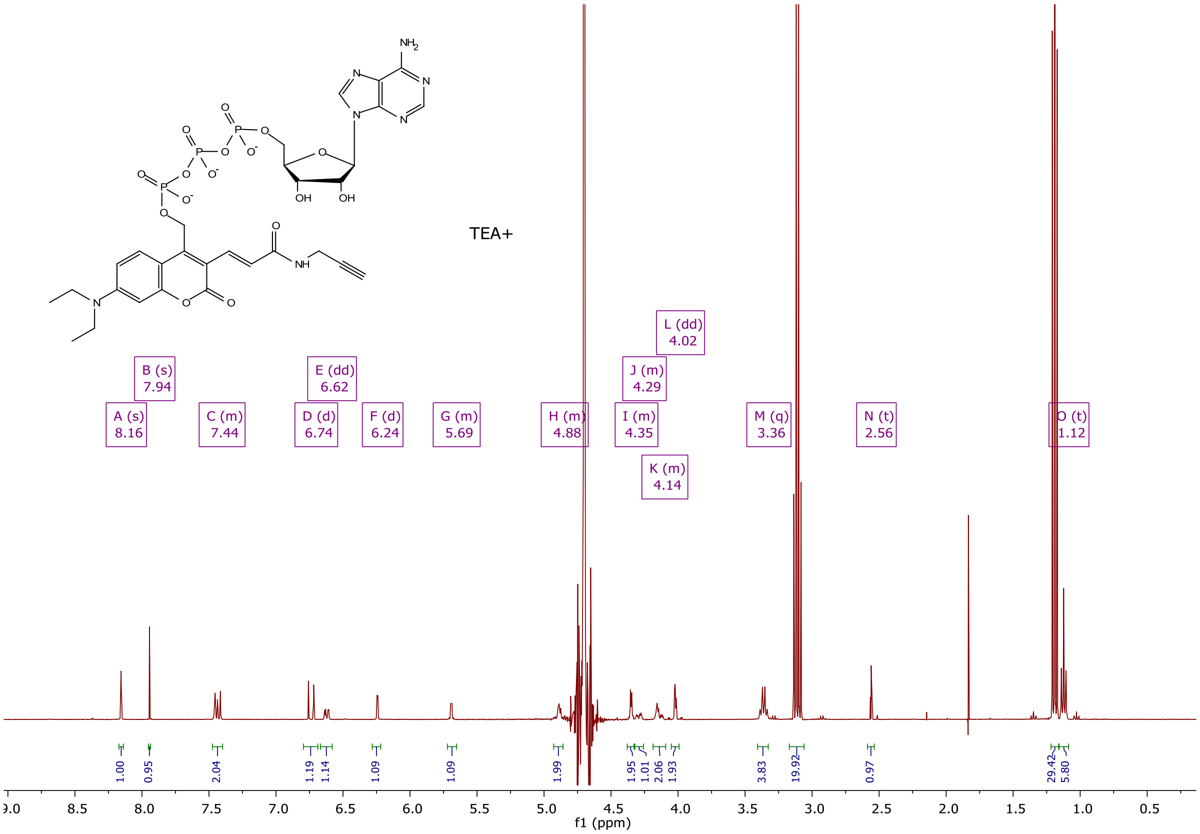

Figure 8.16. ^{1}H NMR spectrum of **6**.

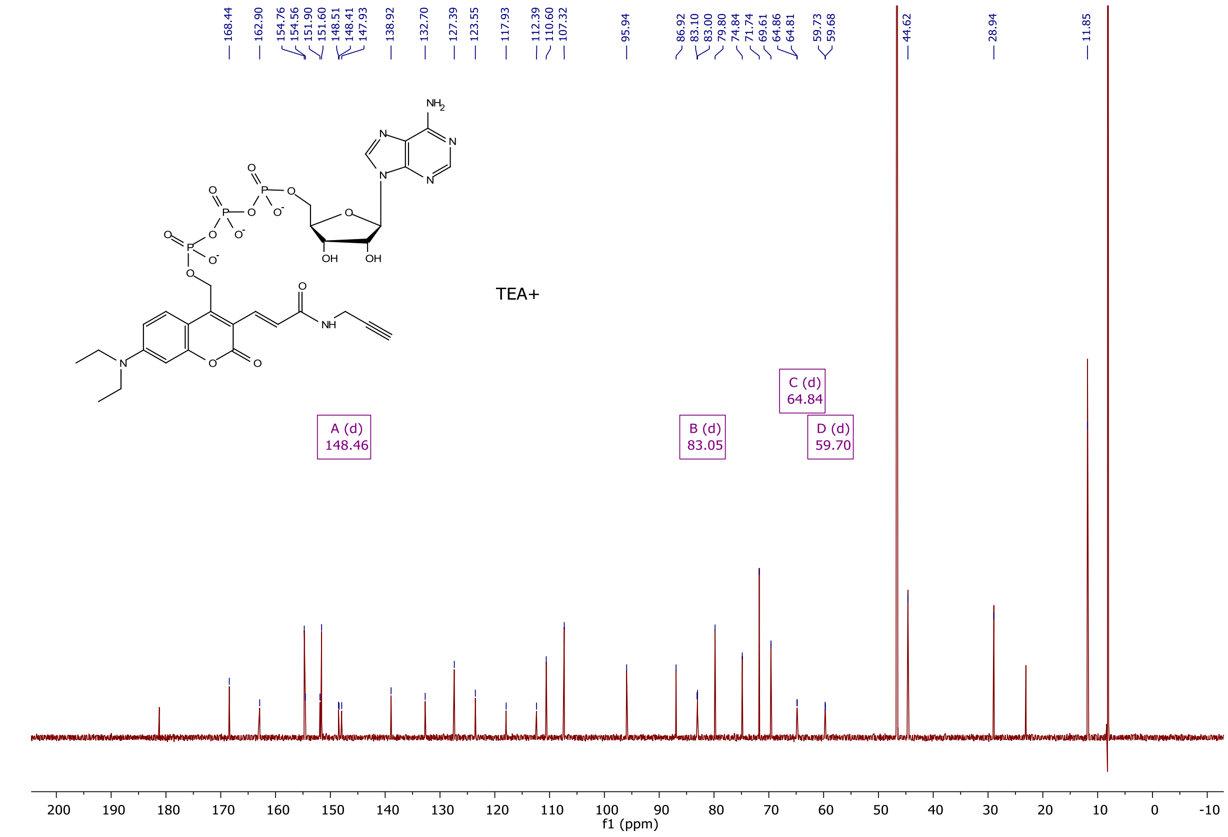

Figure 8.17. ^{13}C NMR spectrum of **6**.

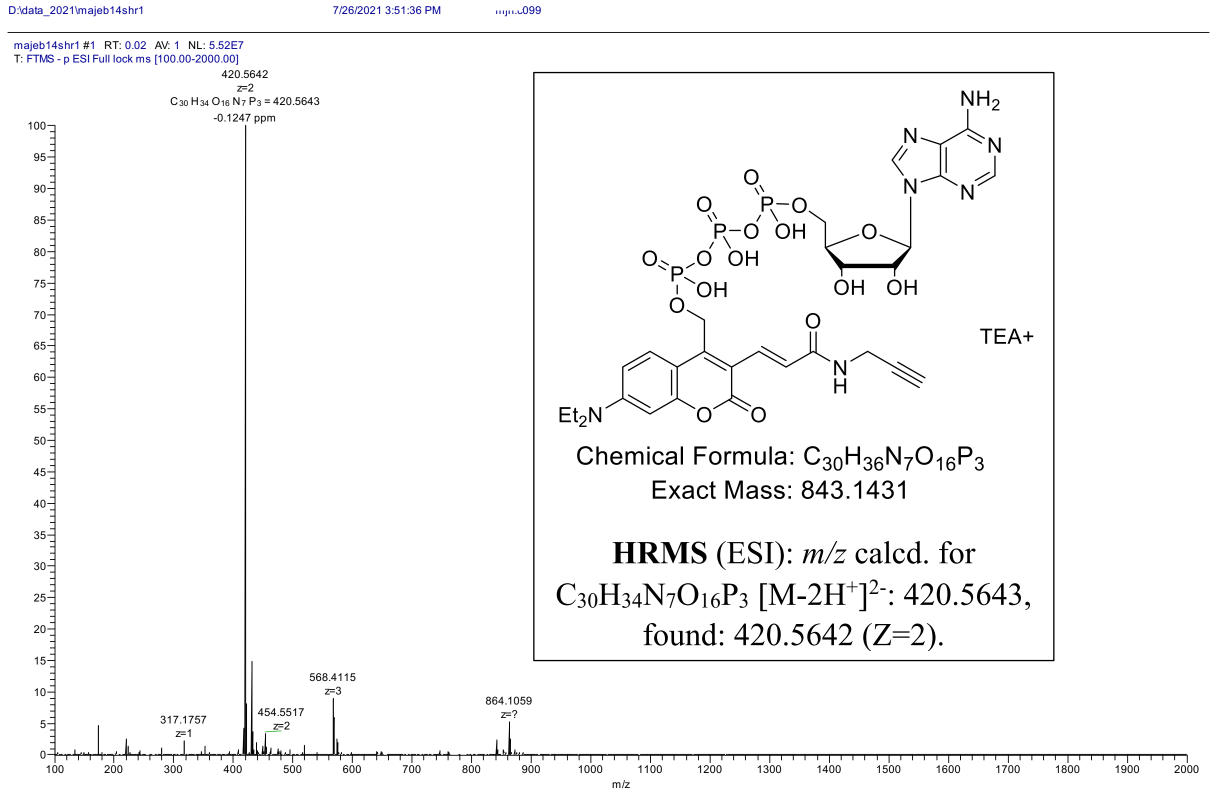

Figure 8.18. MASS spectrum of **6**.

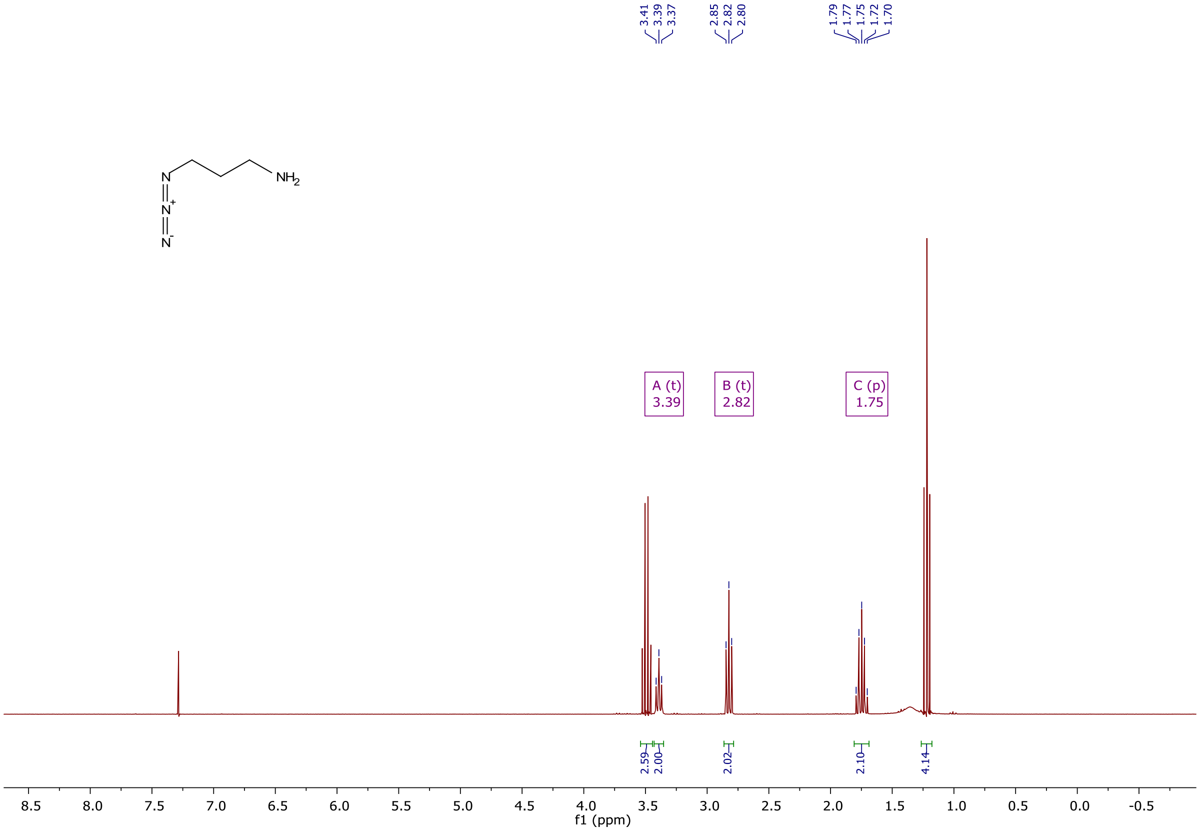

Figure 8.19. **^{1}H** NMR spectrum of **7**.

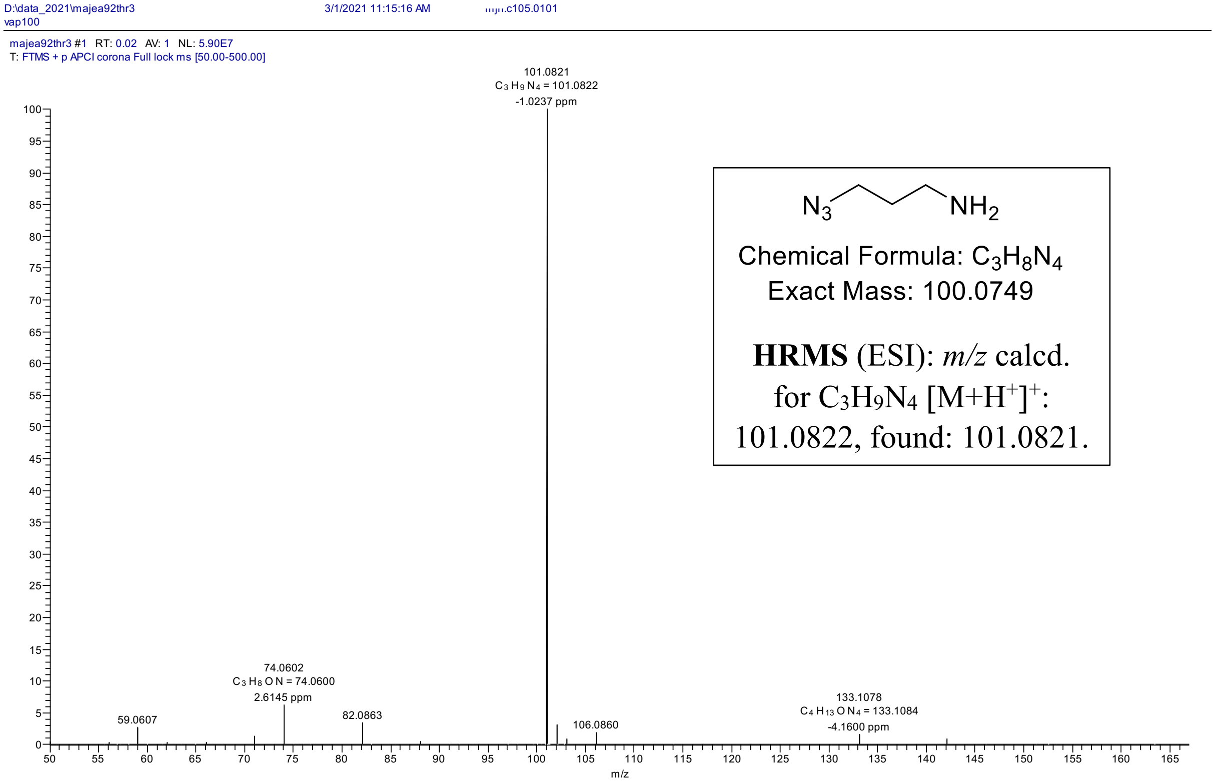

Figure 8.20. **MASS** spectrum of **7**.

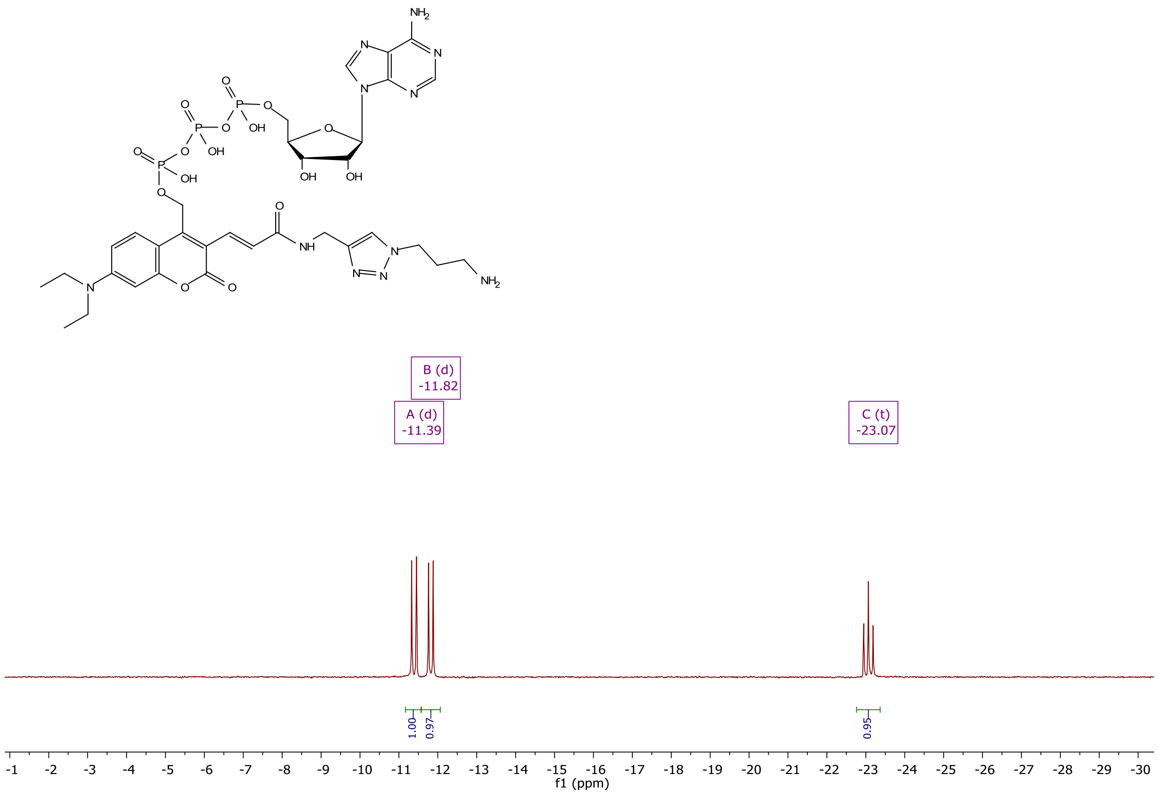

Figure 8.21. ^{31}**P** NMR spectrum of **15**.

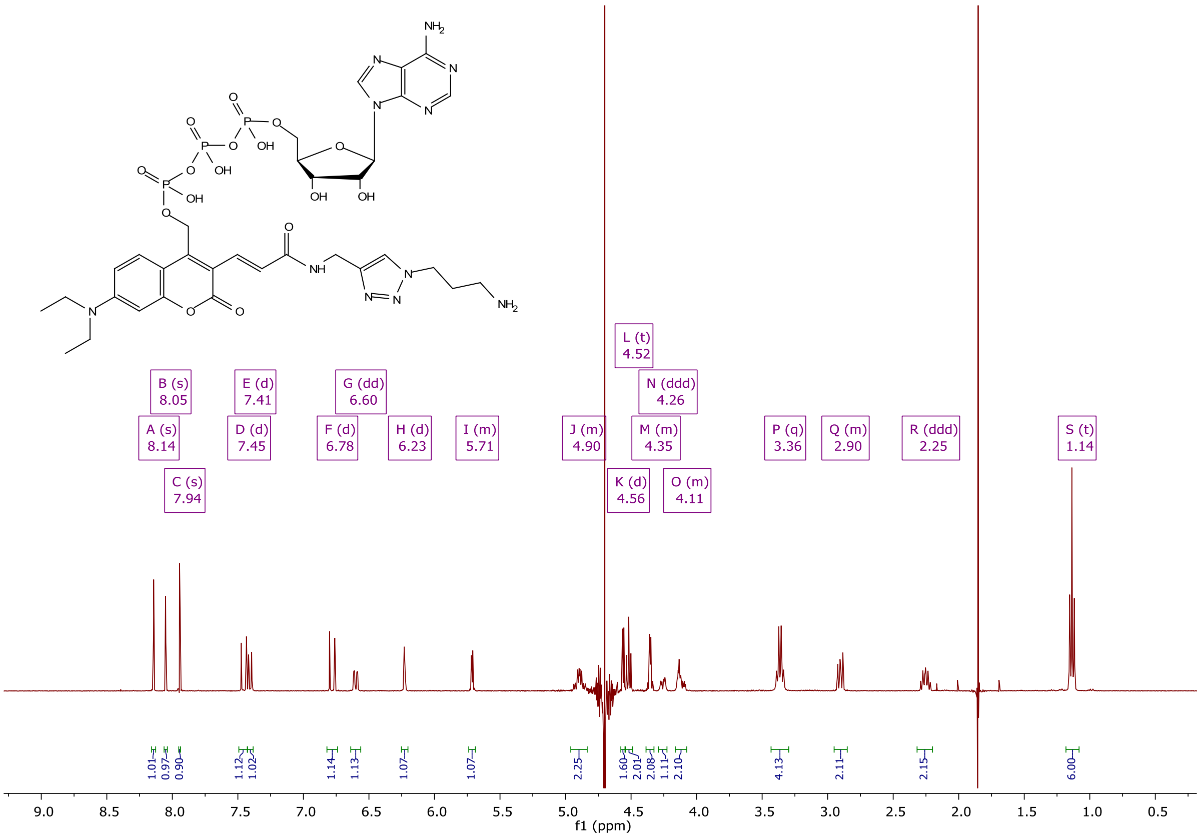

Figure 8.22. ^{1}**H** NMR spectrum of **15**.

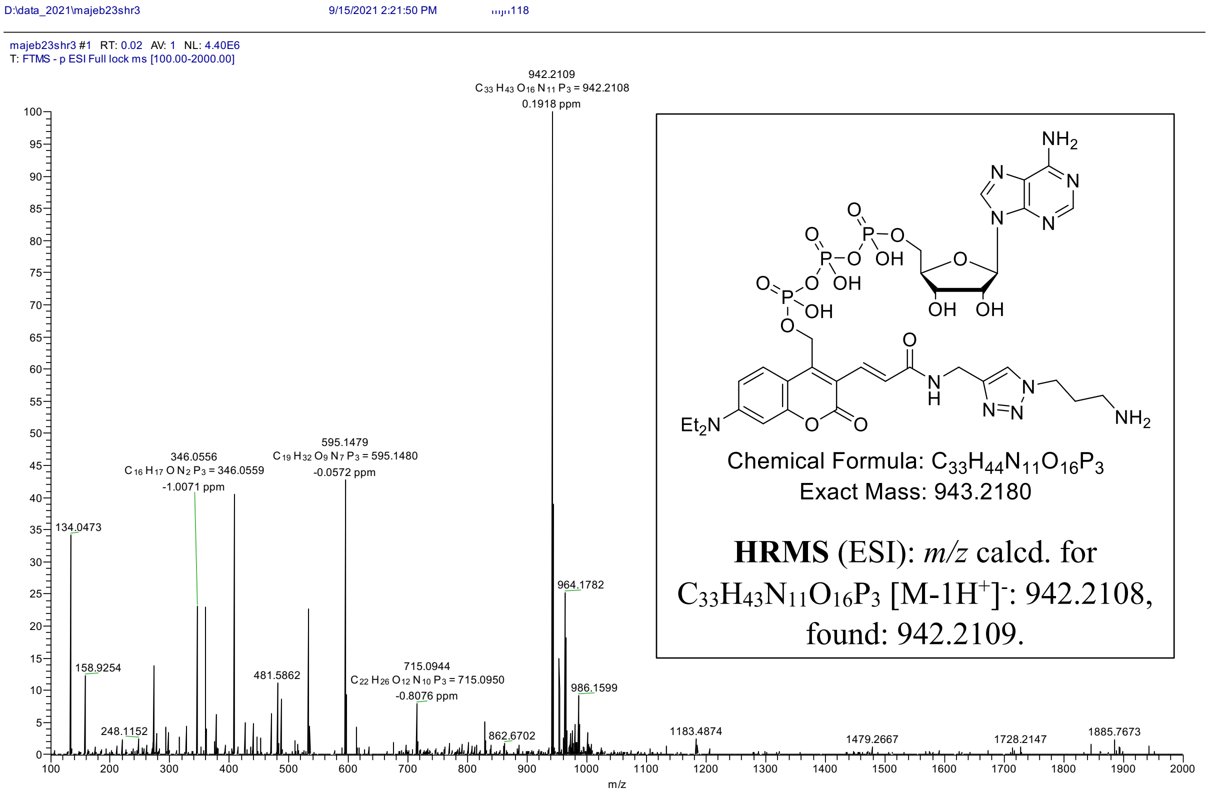

Figure 8.23. MASS spectrum of **15**.

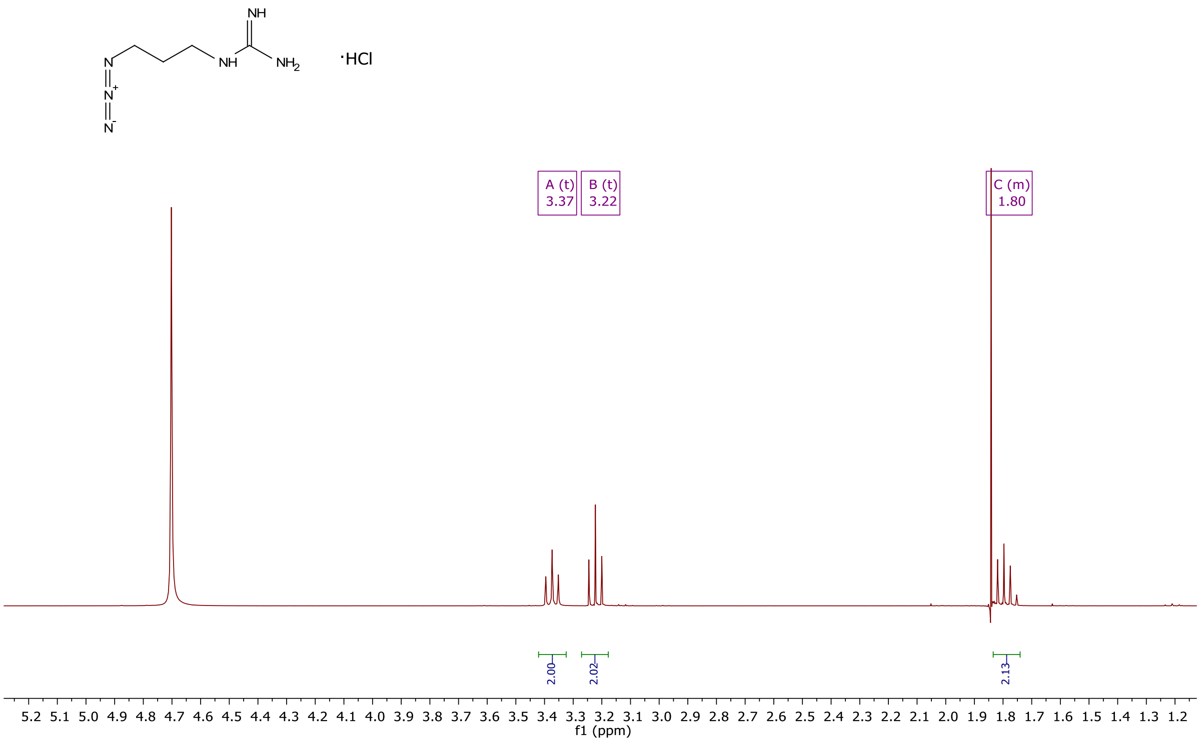

Figure 8.24. ^{1}**H** NMR spectrum of **8**.

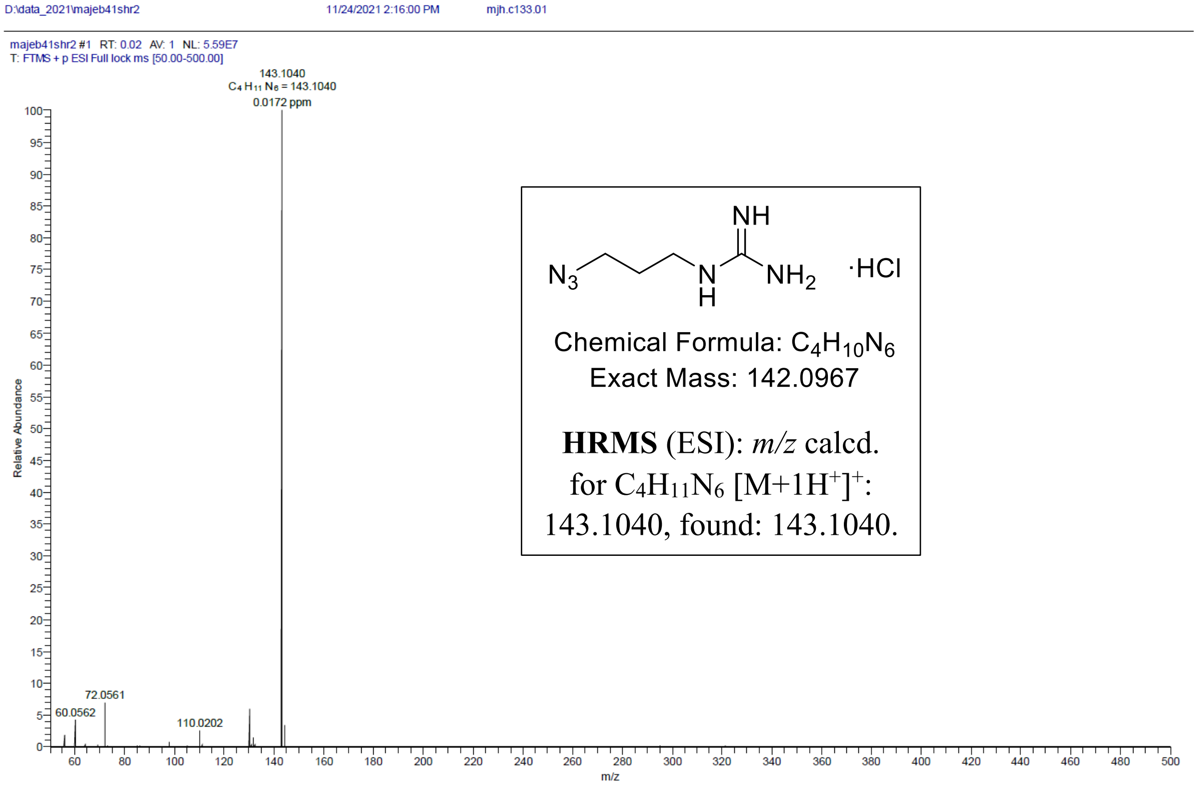

Figure 8.25. MASS spectrum of **8**.

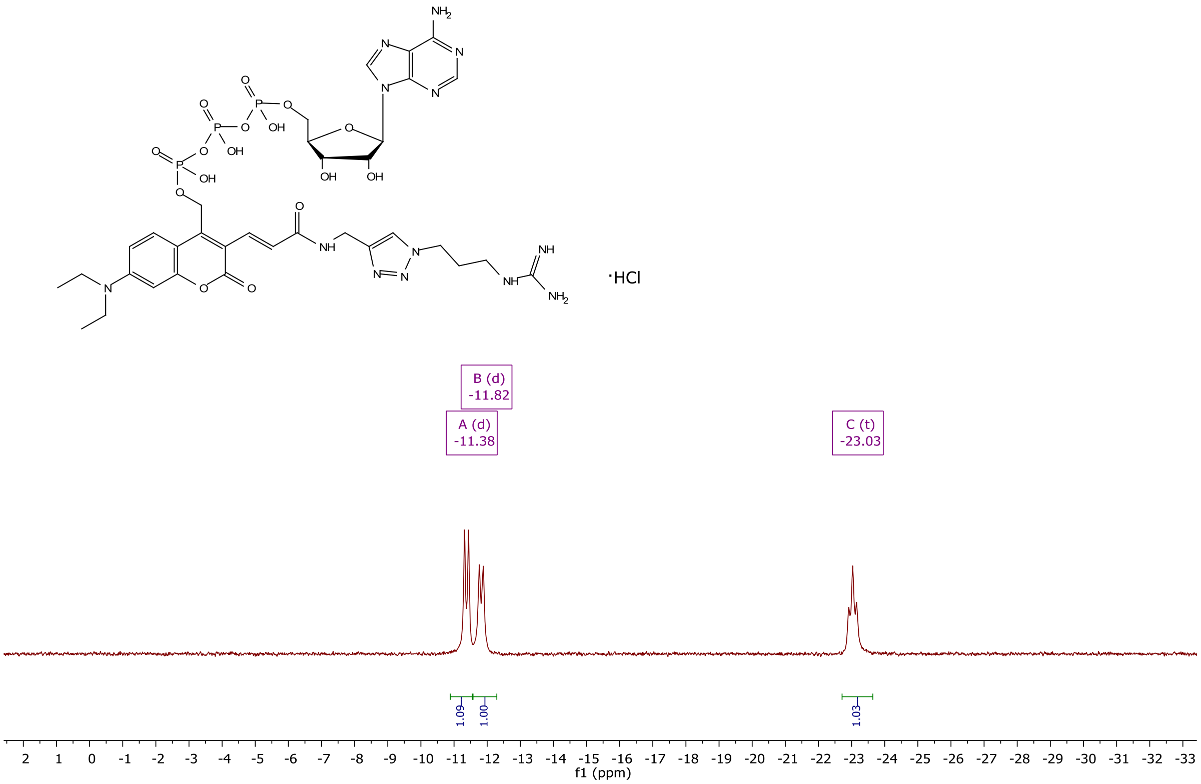

Figure 8.26. ^{31}**P** NMR spectrum of **16**.

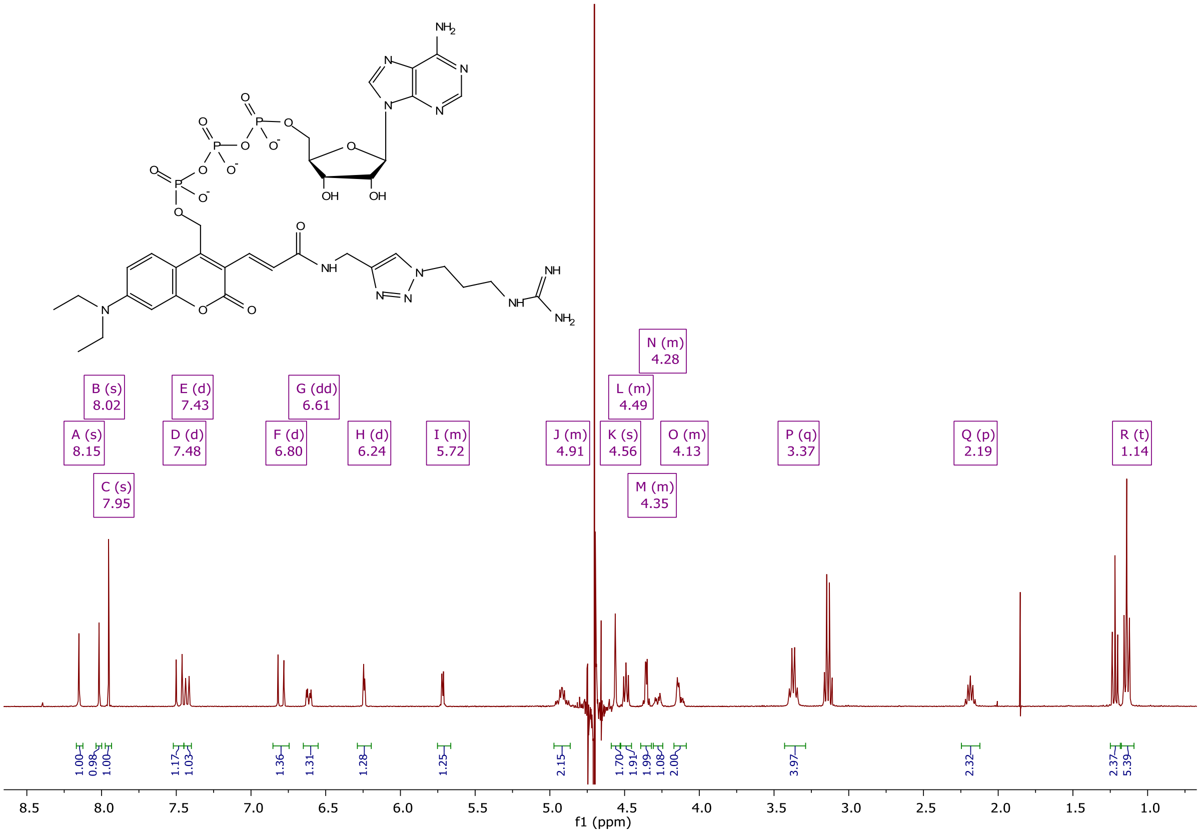

Figure 8.27. ^{1}**H** NMR spectrum of **16**.

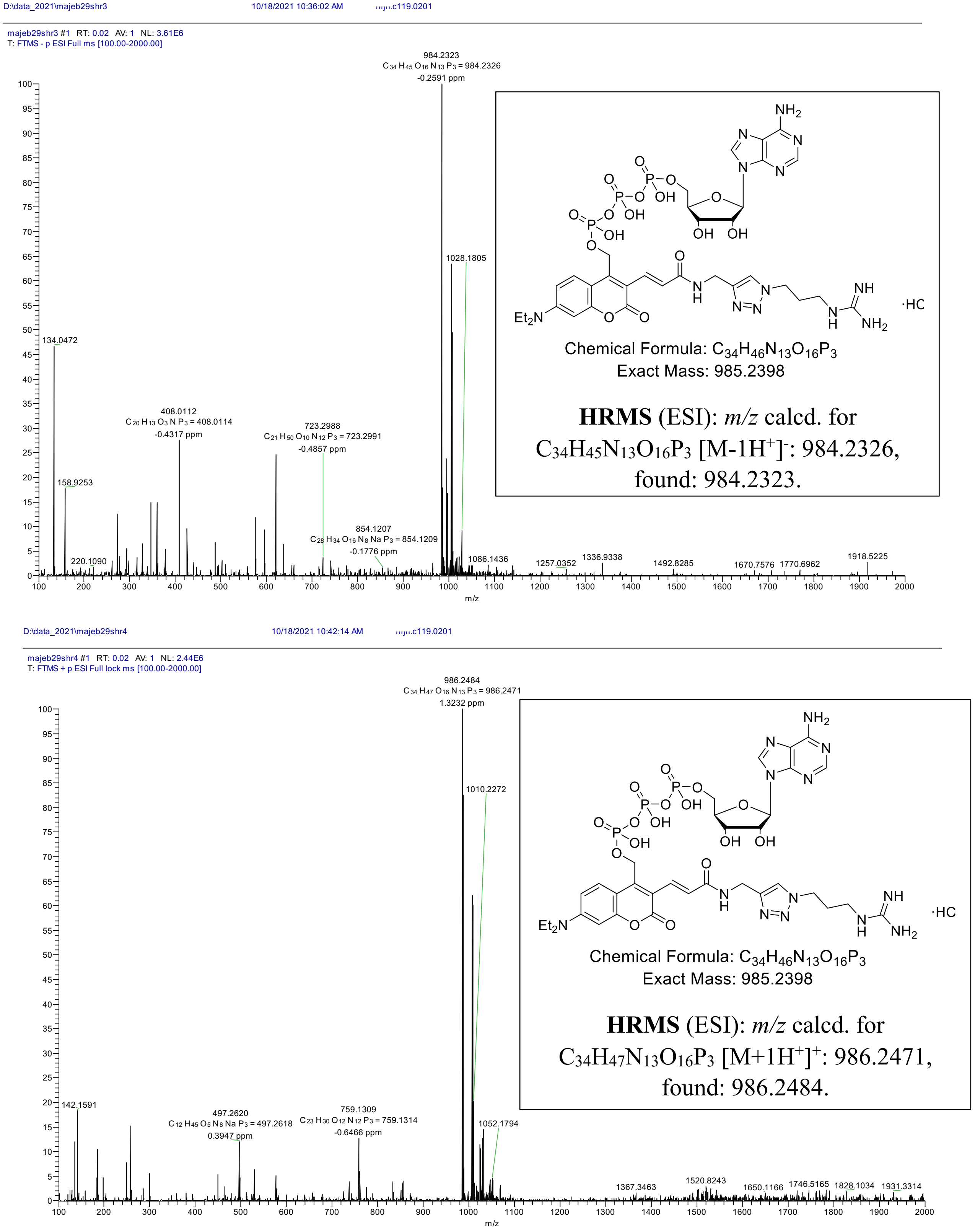

Figure 8.28. **MASS** spectra of **16**.

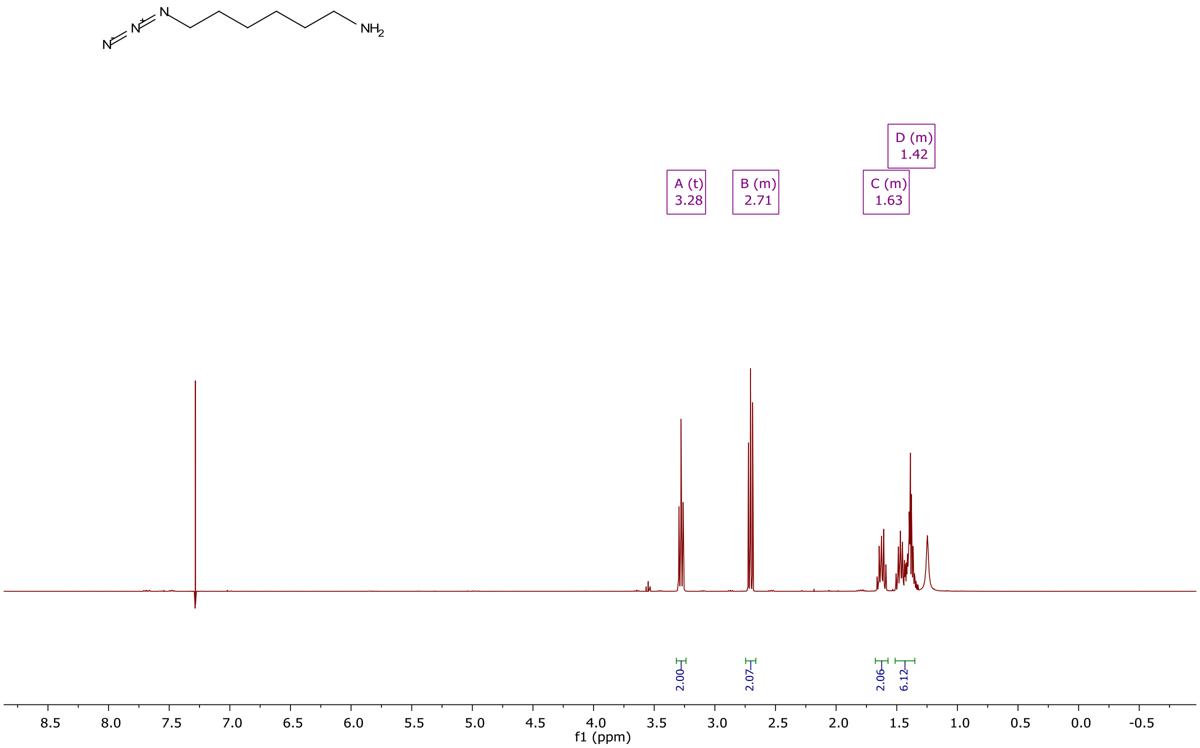

Figure 8.29. ^{1}**H** NMR spectrum of **9**.

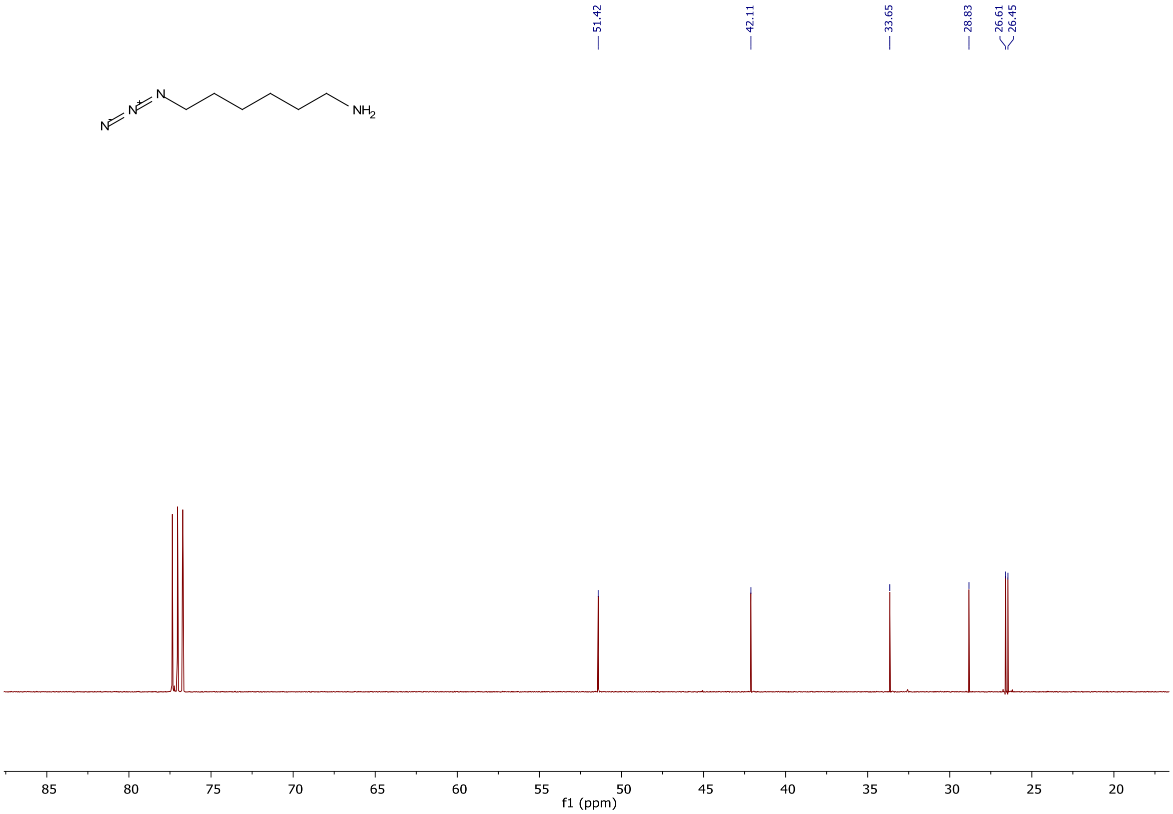

Figure 8.30. ^{13}**C** NMR spectrum of **9**.

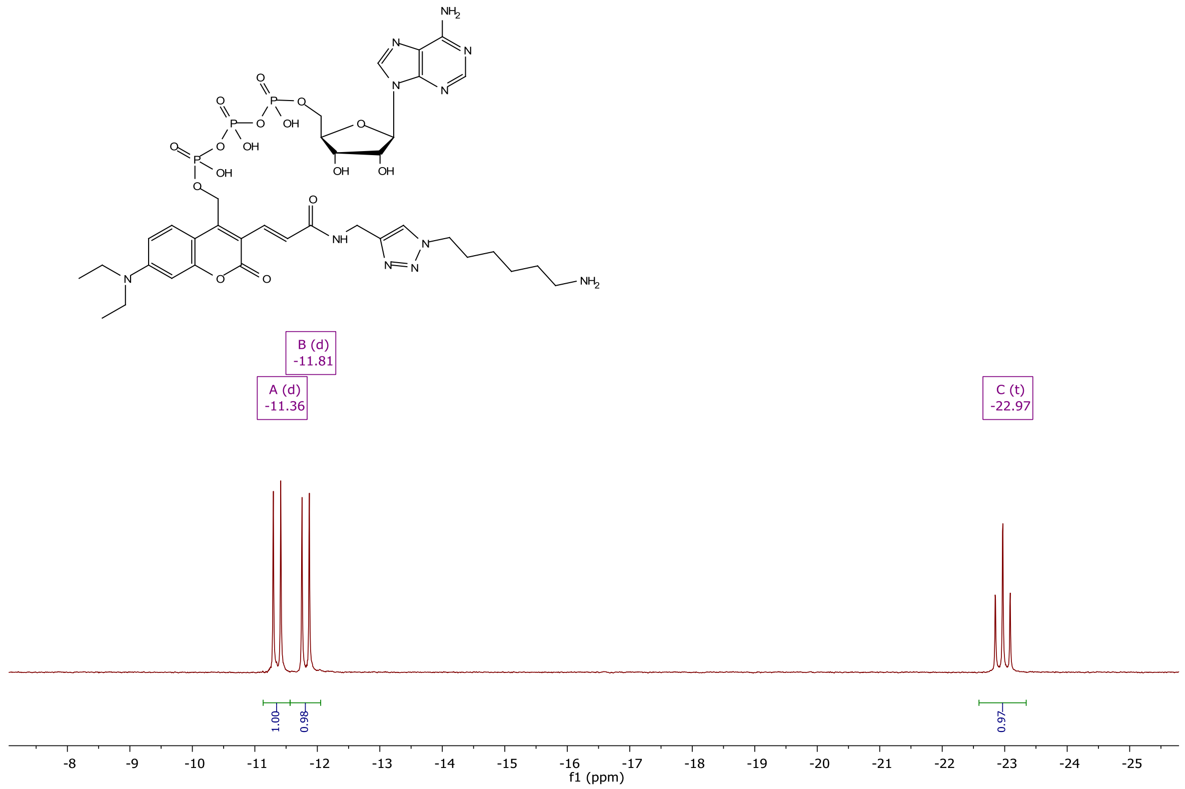

Figure 8.31. ^{31}**P** NMR spectrum of **17**.

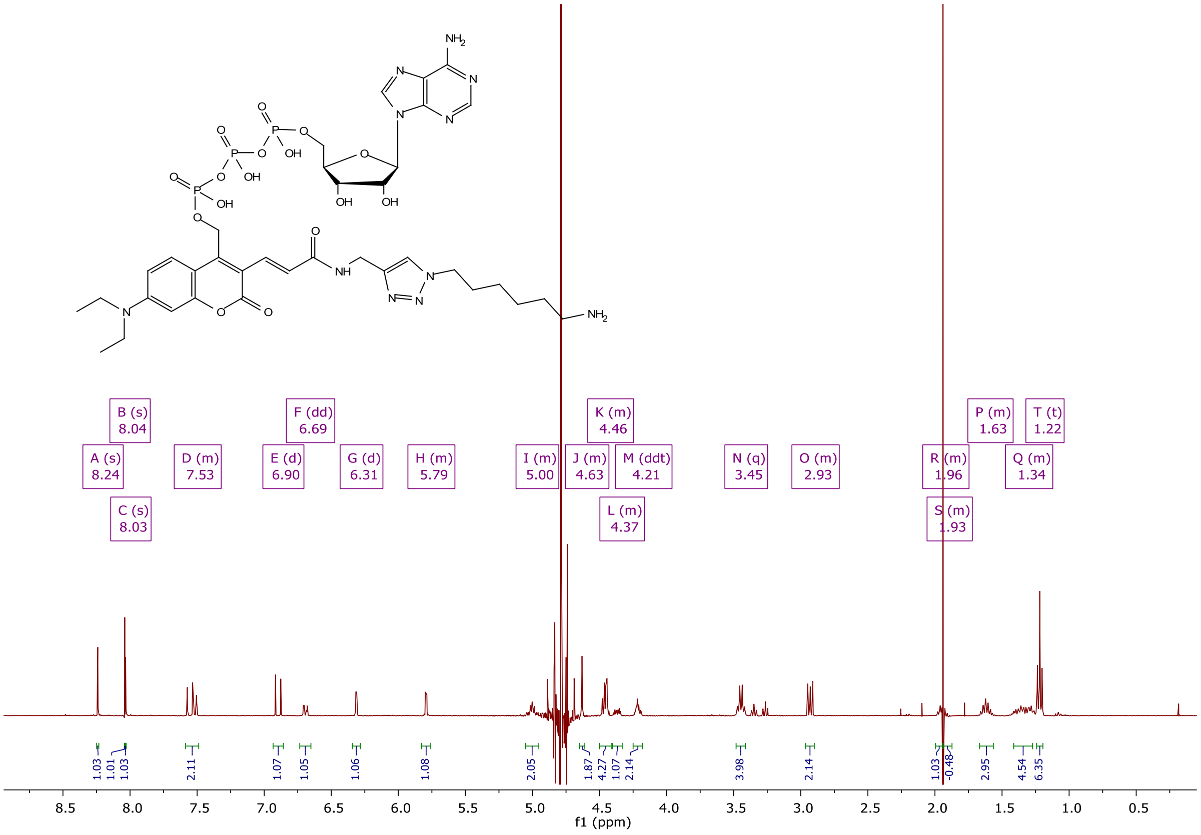

Figure 8.32. ^{1}**H** NMR spectrum of **17**.

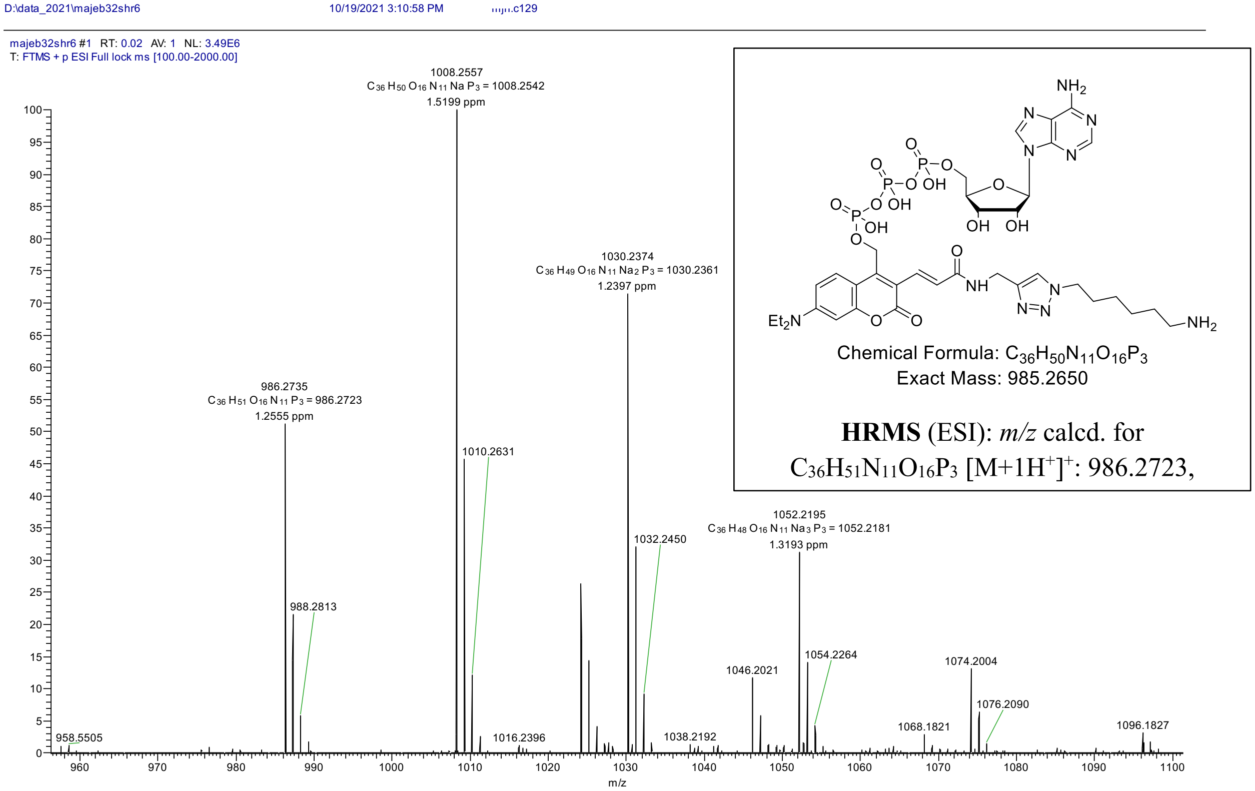

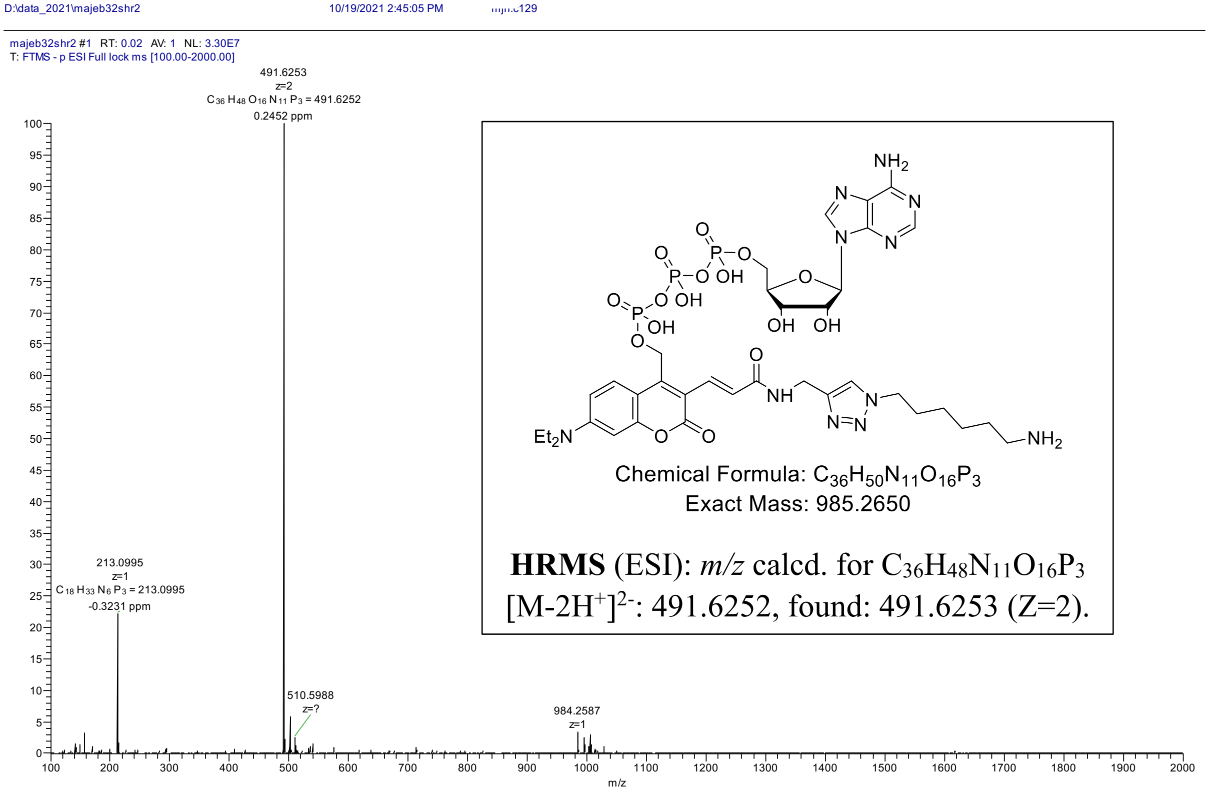

Figure 8.33. MASS spectra of **17**.

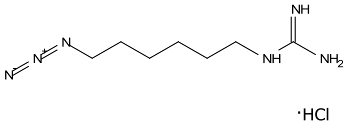

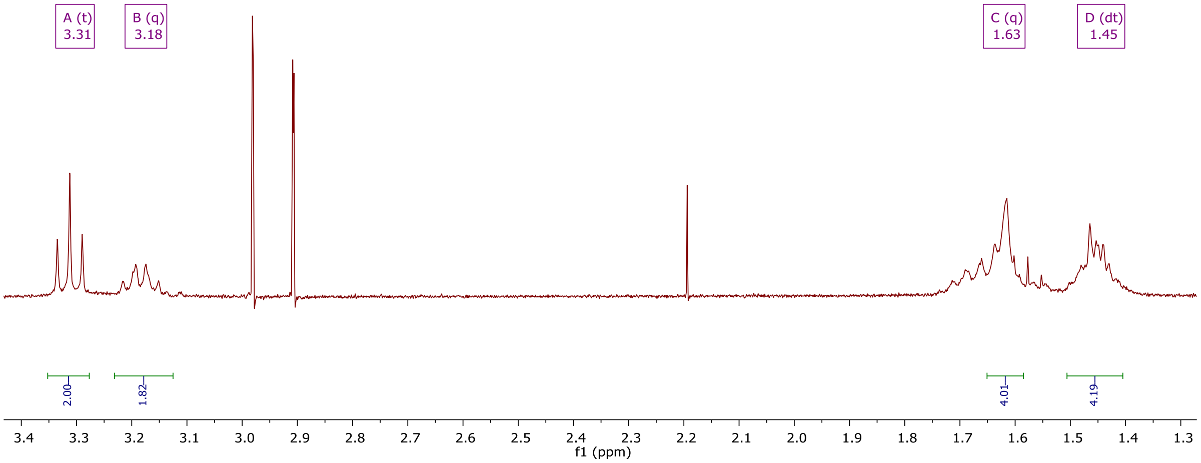

Figure 8.34. ^{1}**H** NMR spectrum of **10**.

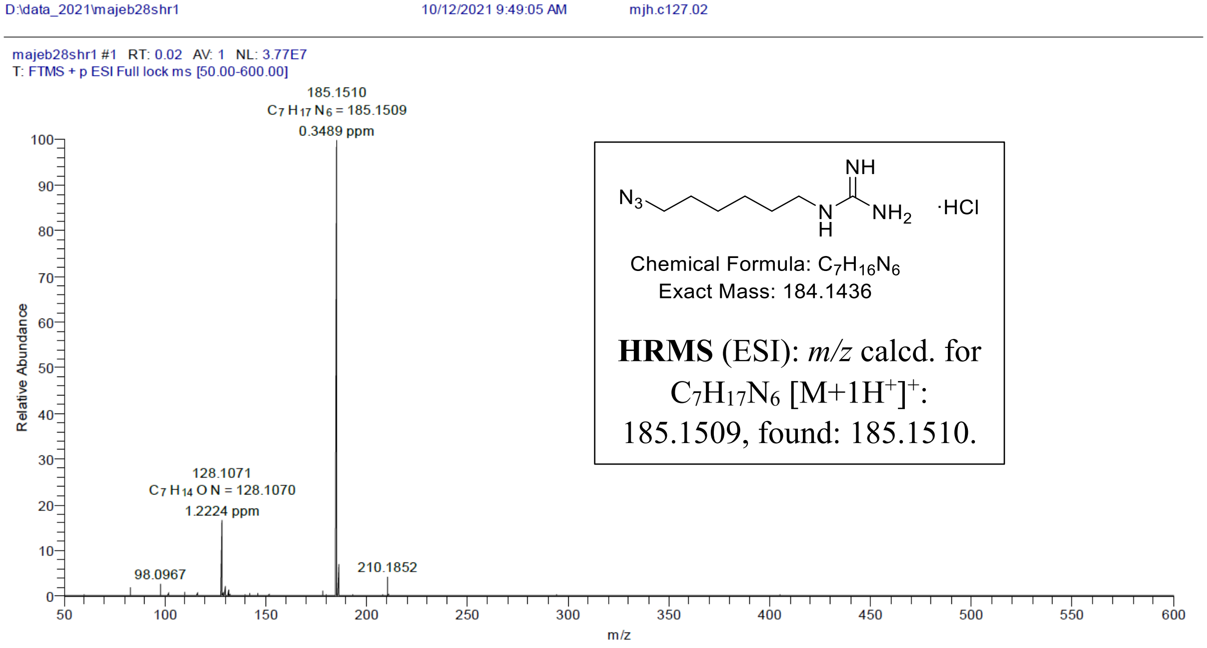

Figure 8.35. MASS spectrum of **10**.

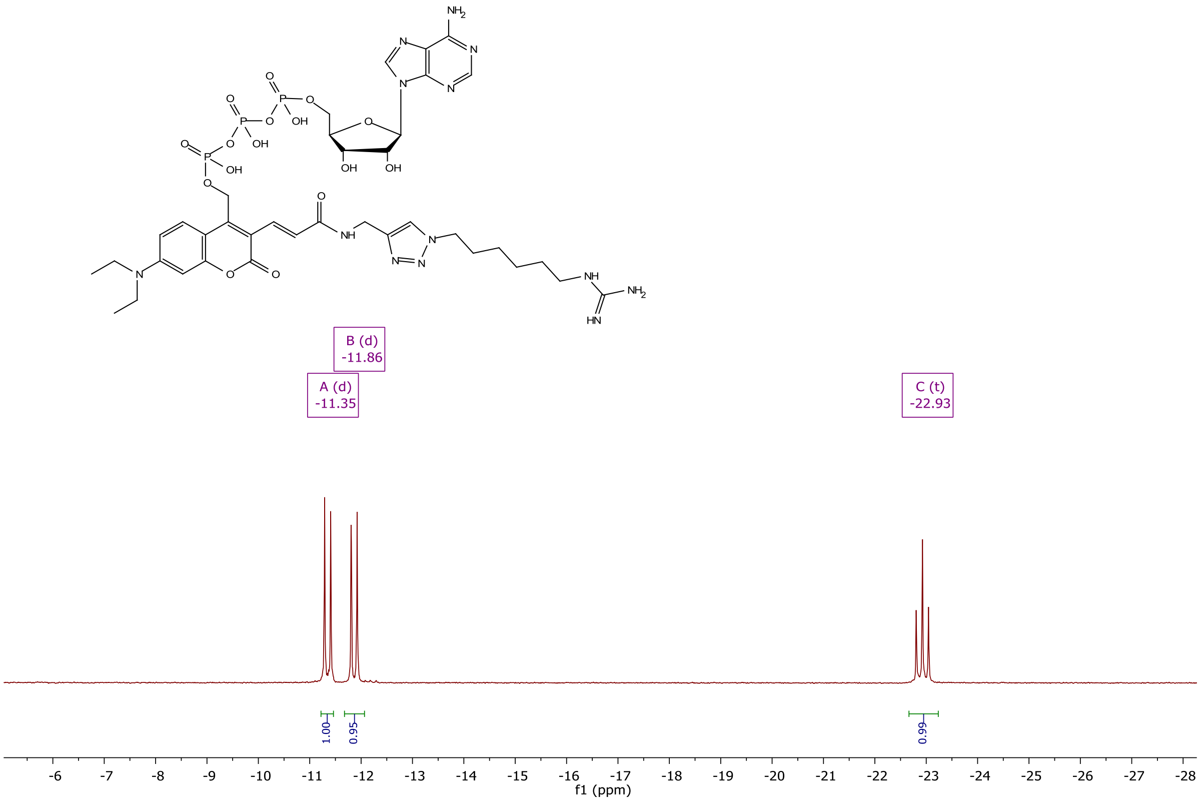

Figure 8.36. ^{31}**P** NMR spectrum of **18**.

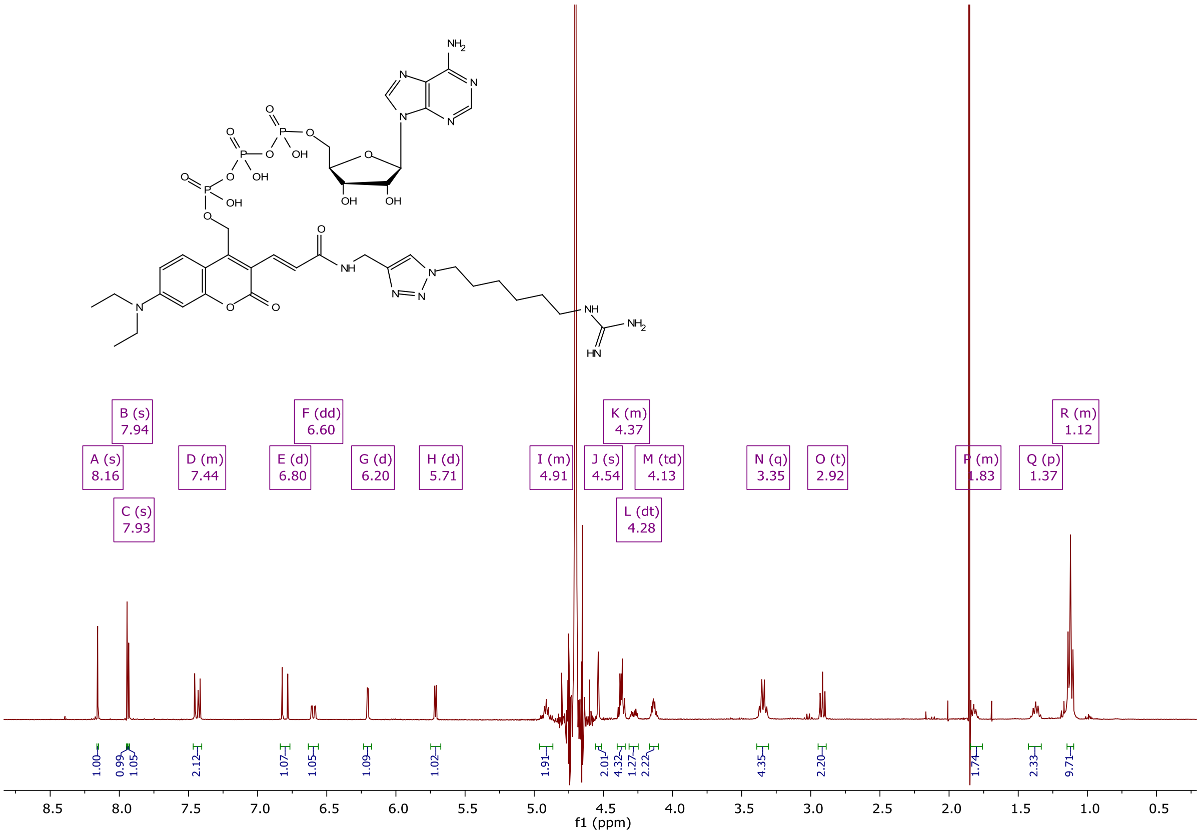

Figure 8.37. **^{1}H** NMR spectrum of **18**.

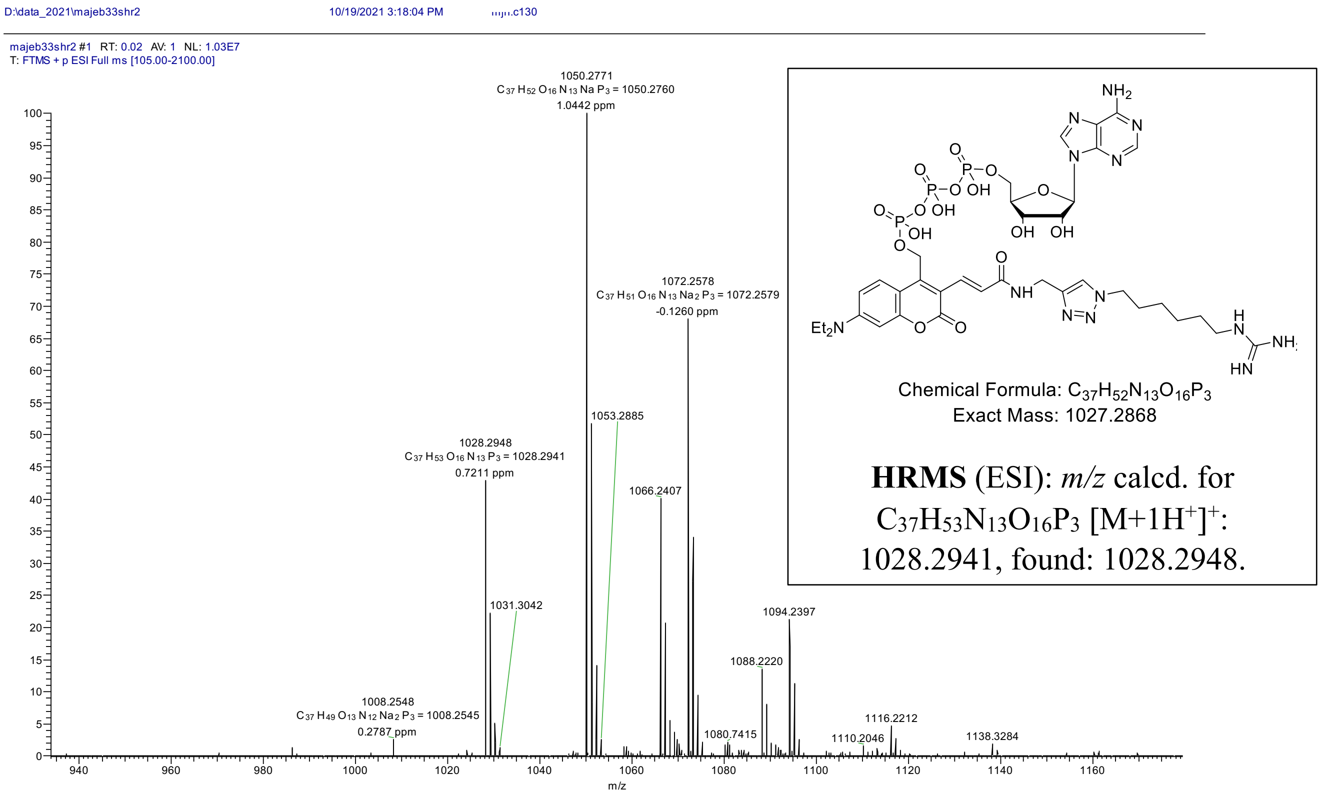

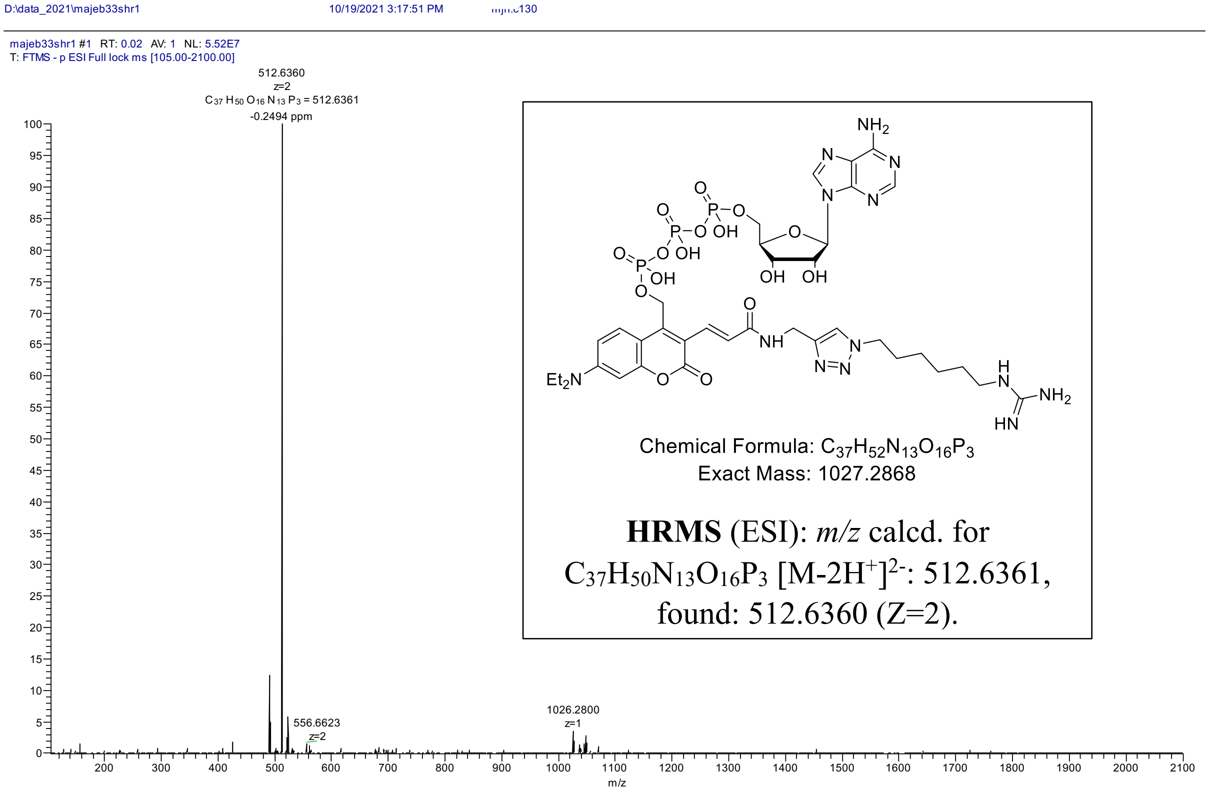

Figure 8.38. MASS spectra of **18**.

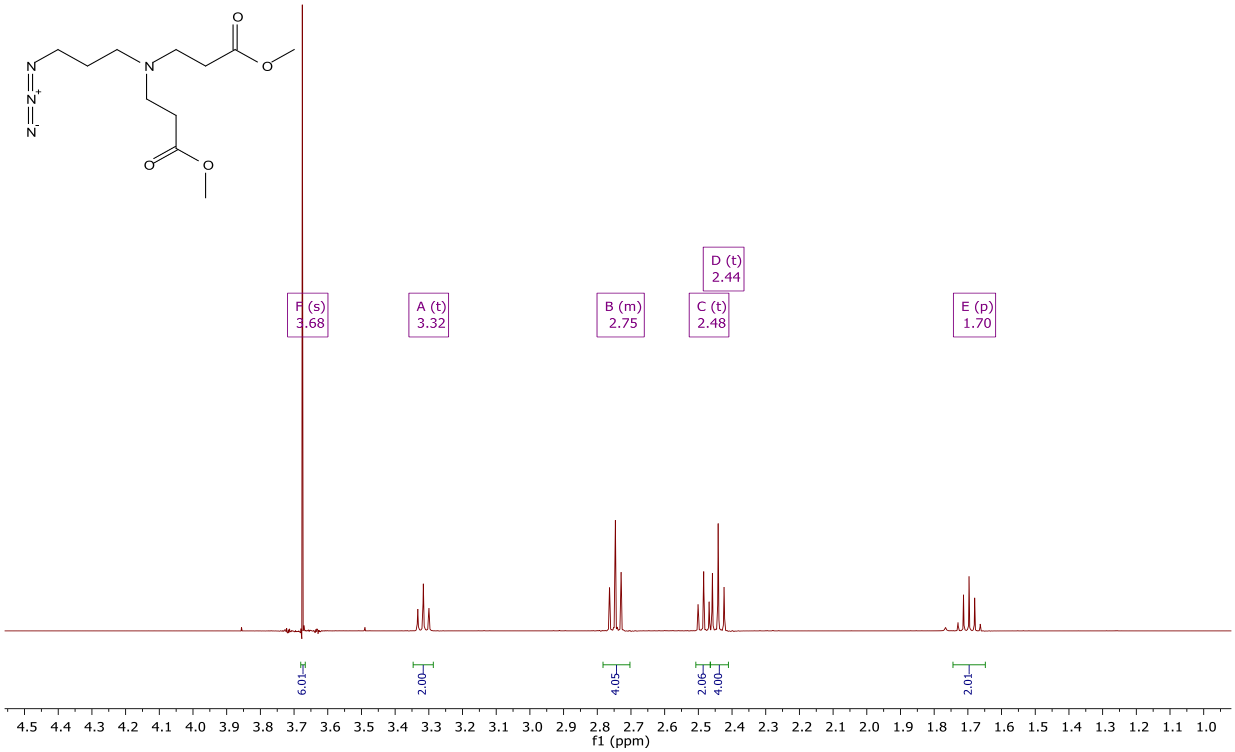

Figure 8.39. ^{1}**H** NMR spectrum of **G0.5 S9**.

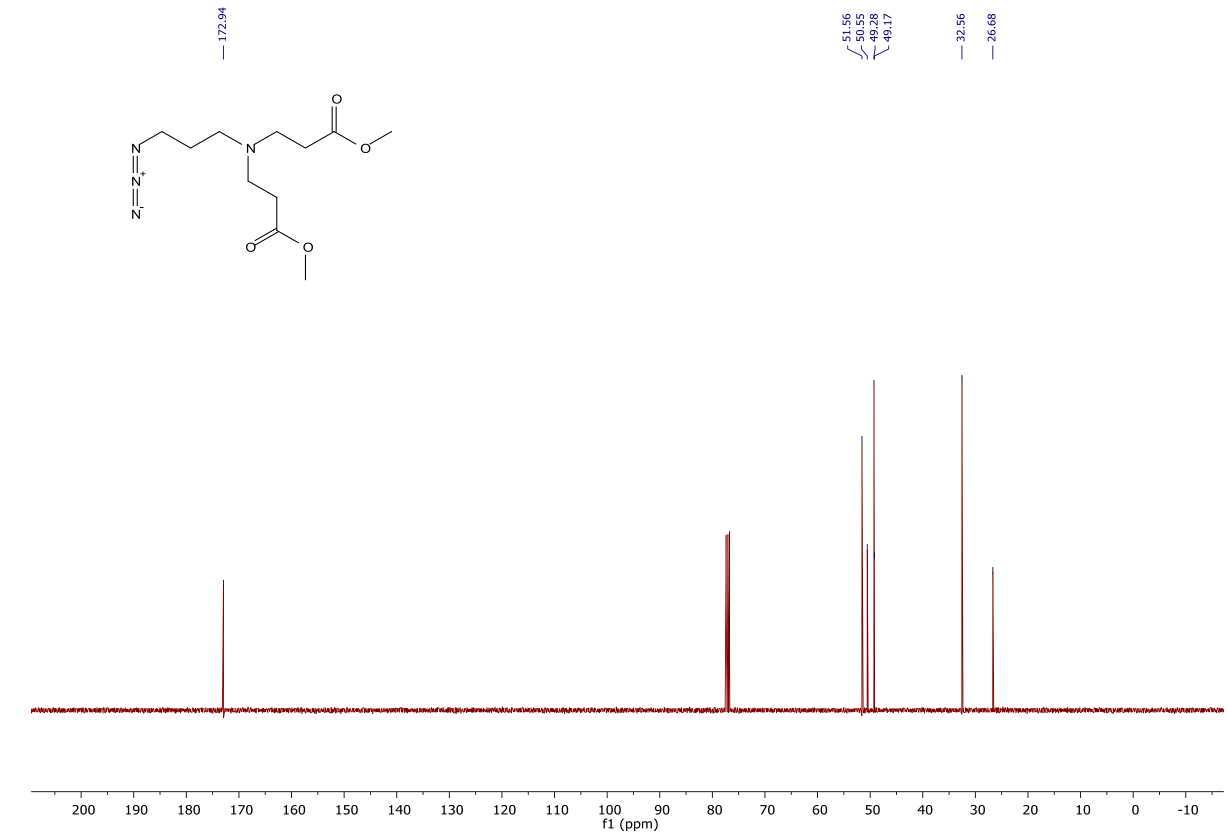

Figure 8.40. ^{13}C NMR spectrum of **G0.5 S9**.

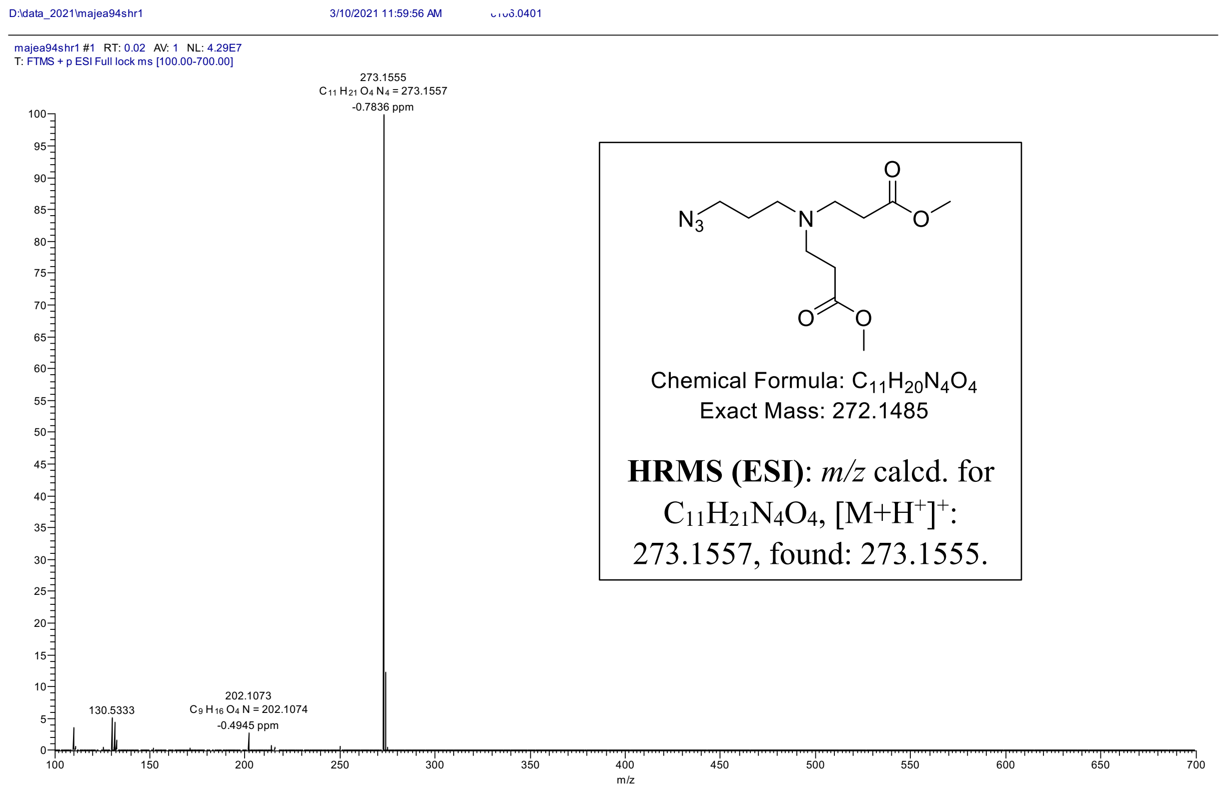

Figure 8.41. MASS spectrum of **G0.5 S9**.

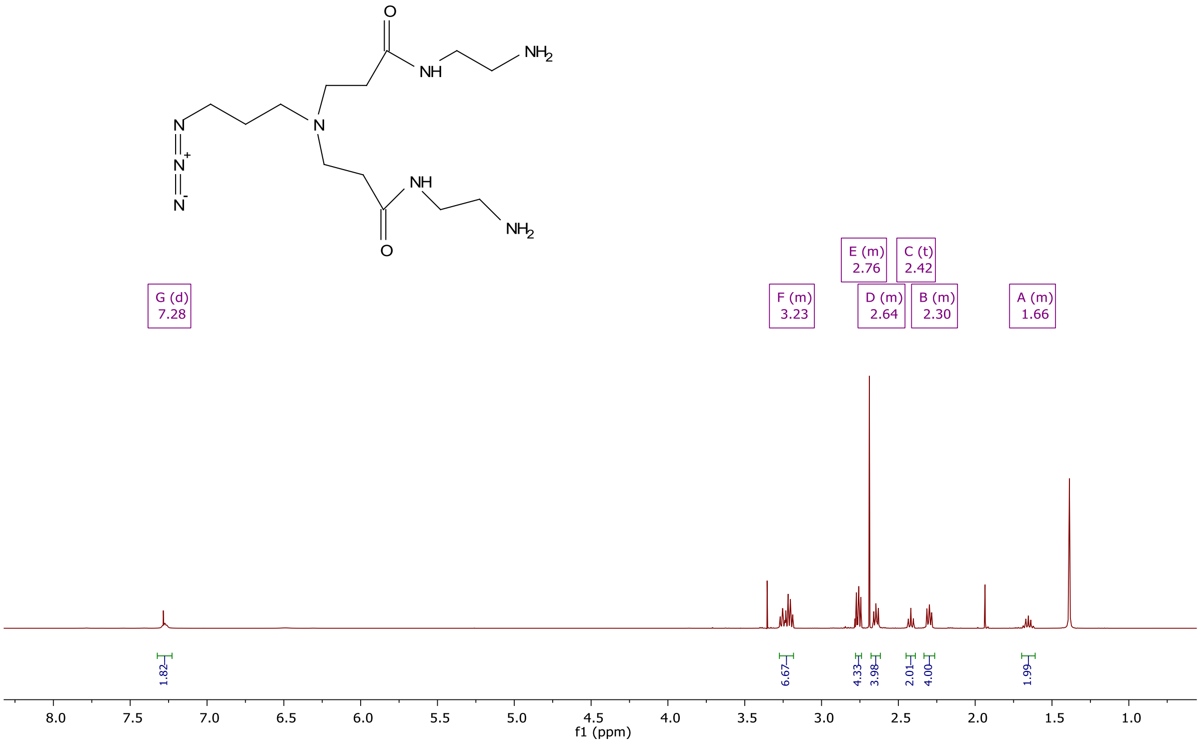

Figure 8.42. ^{1}**H** NMR spectrum of **11**.

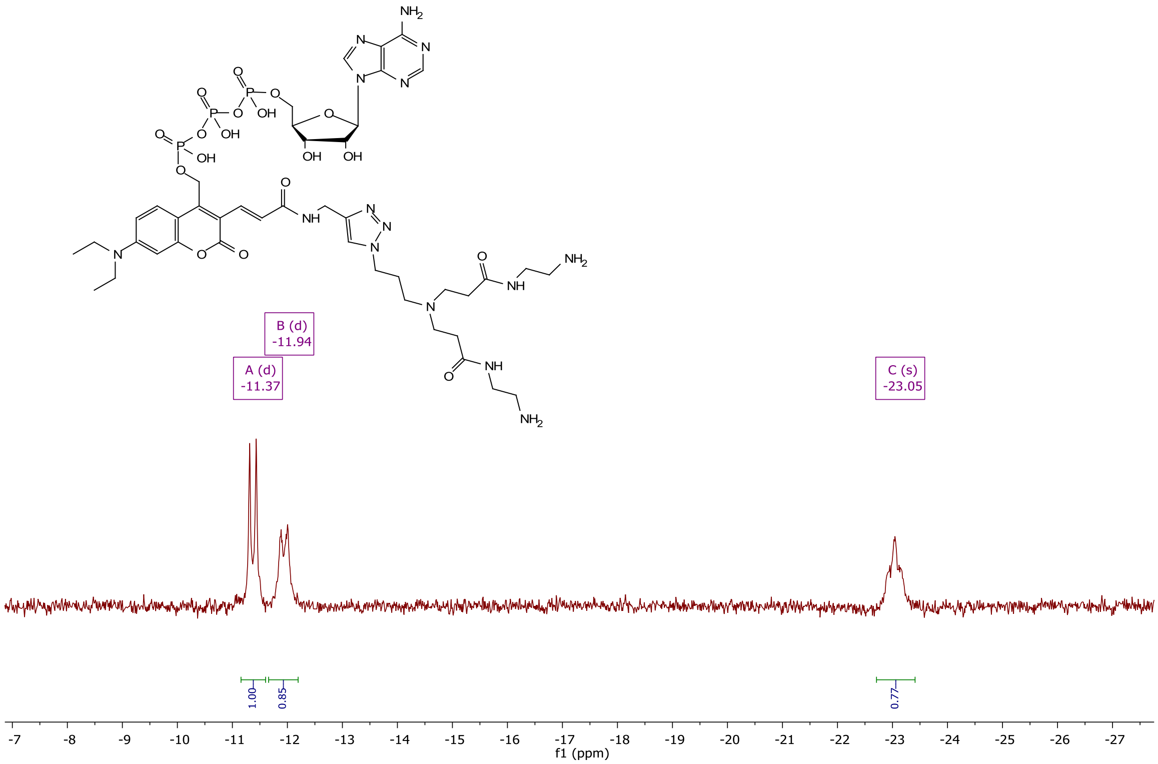

Figure 8.43. ^{31}**P** NMR spectrum of **19**.

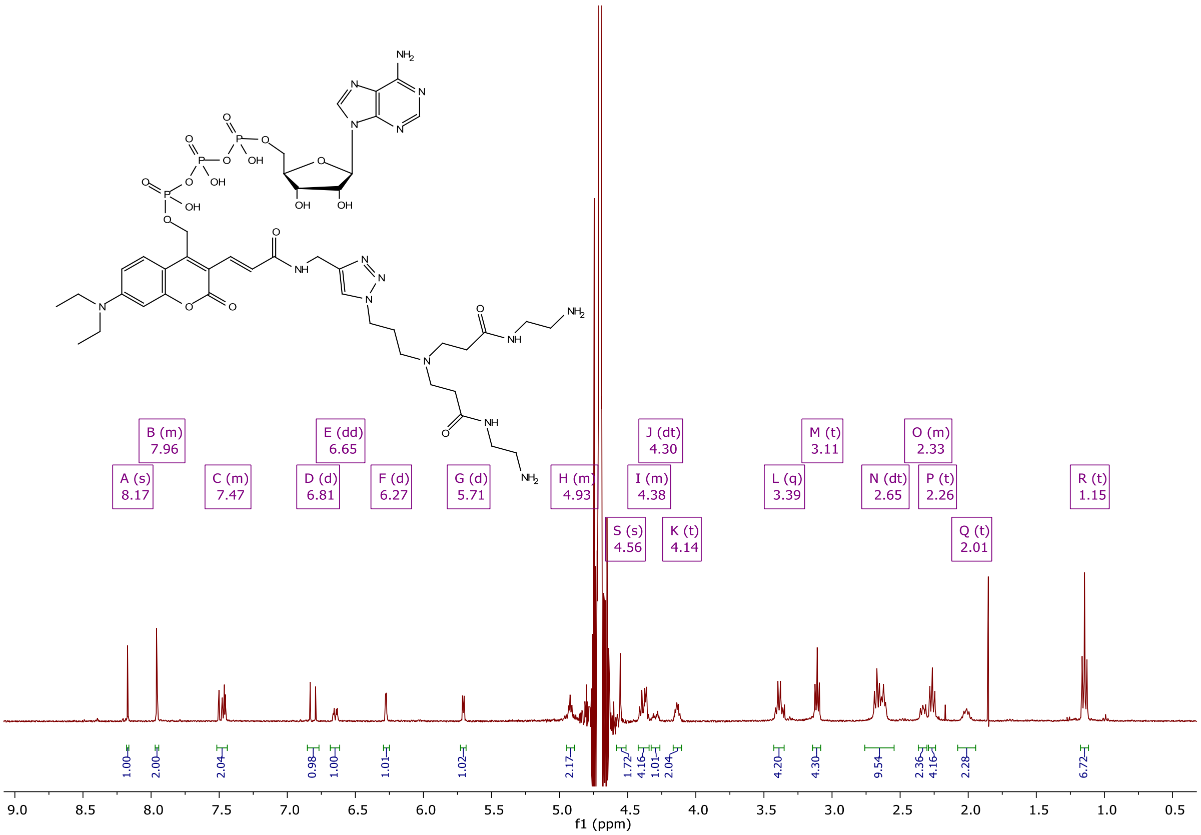

Figure 8.44. **^{1}H** NMR spectrum of **19**.

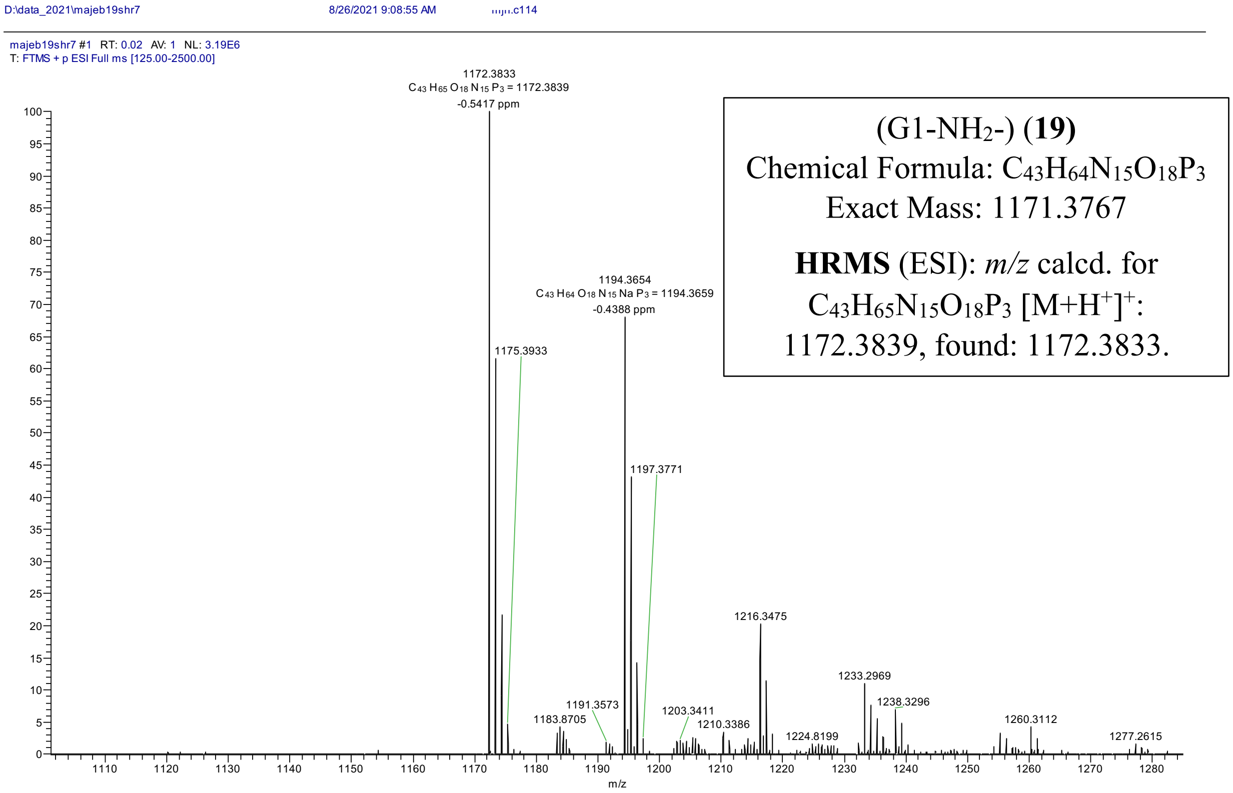

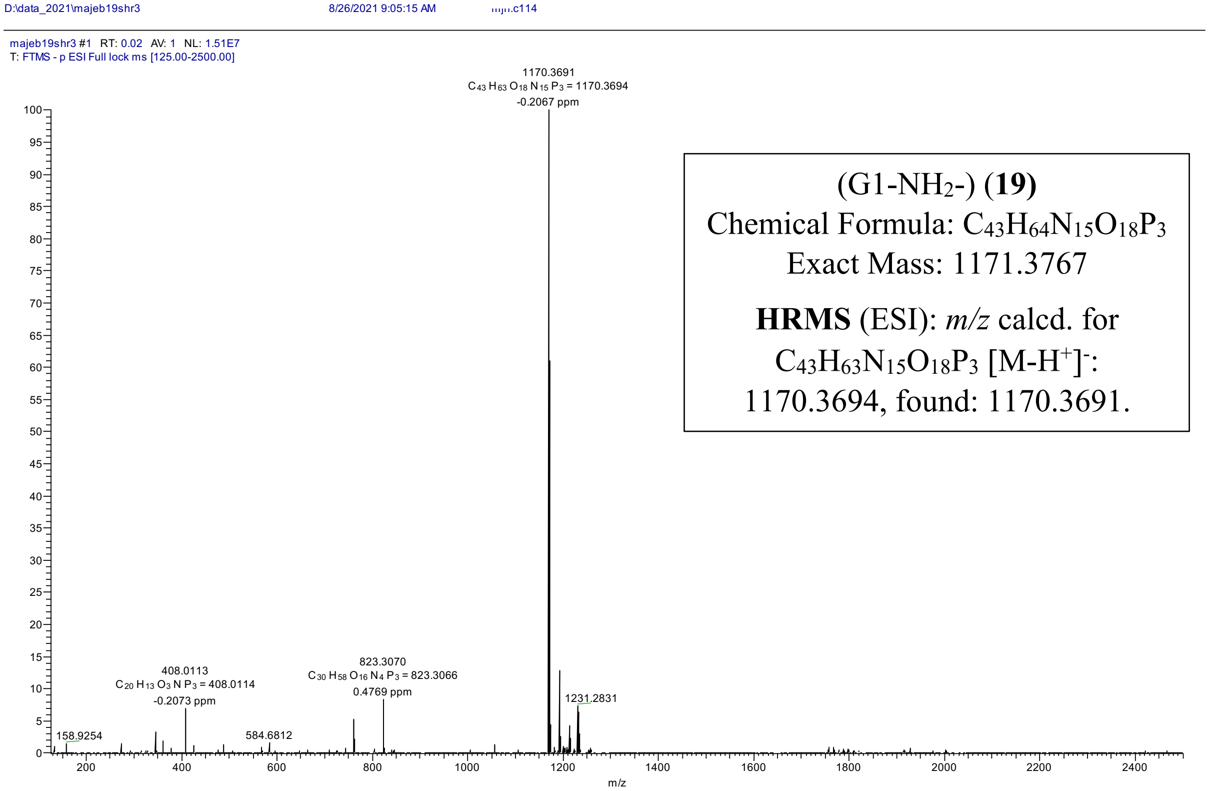

Figure 8.45. MASS spectra of **19**.

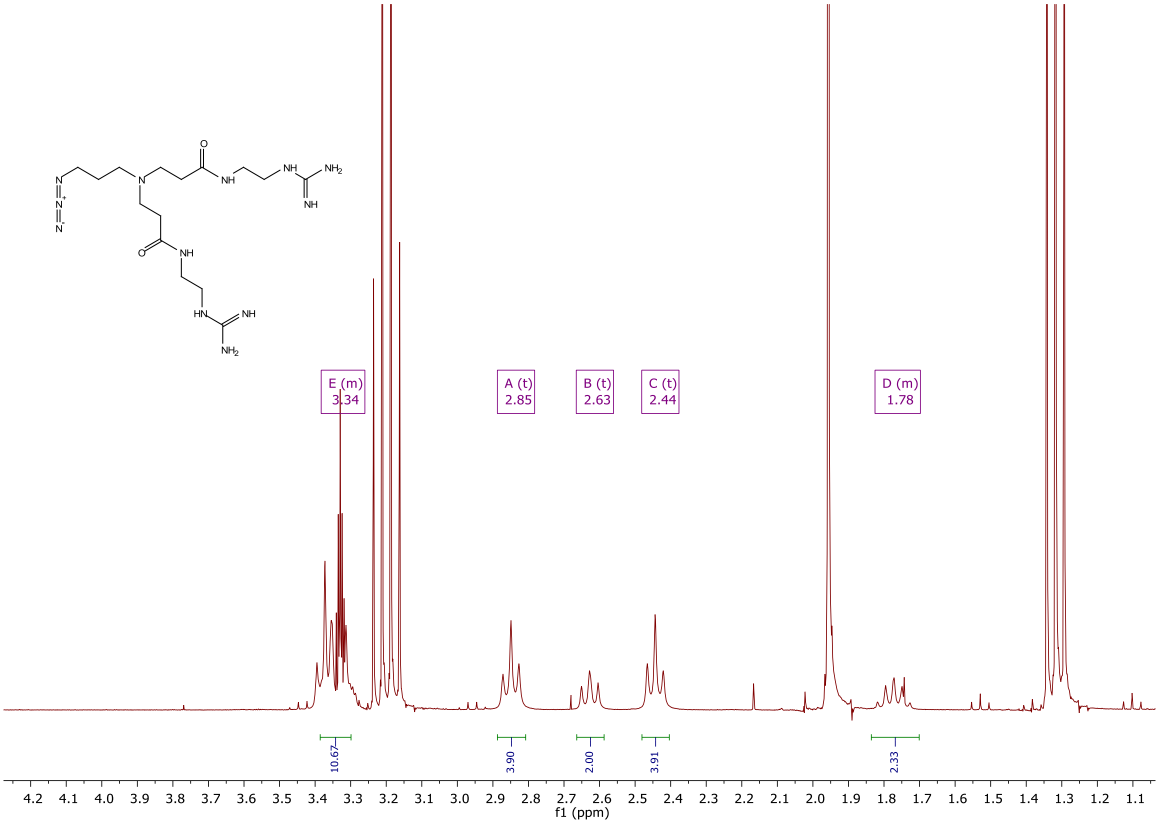

Figure 8.46. ^{1}H NMR spectrum of compound **12**.

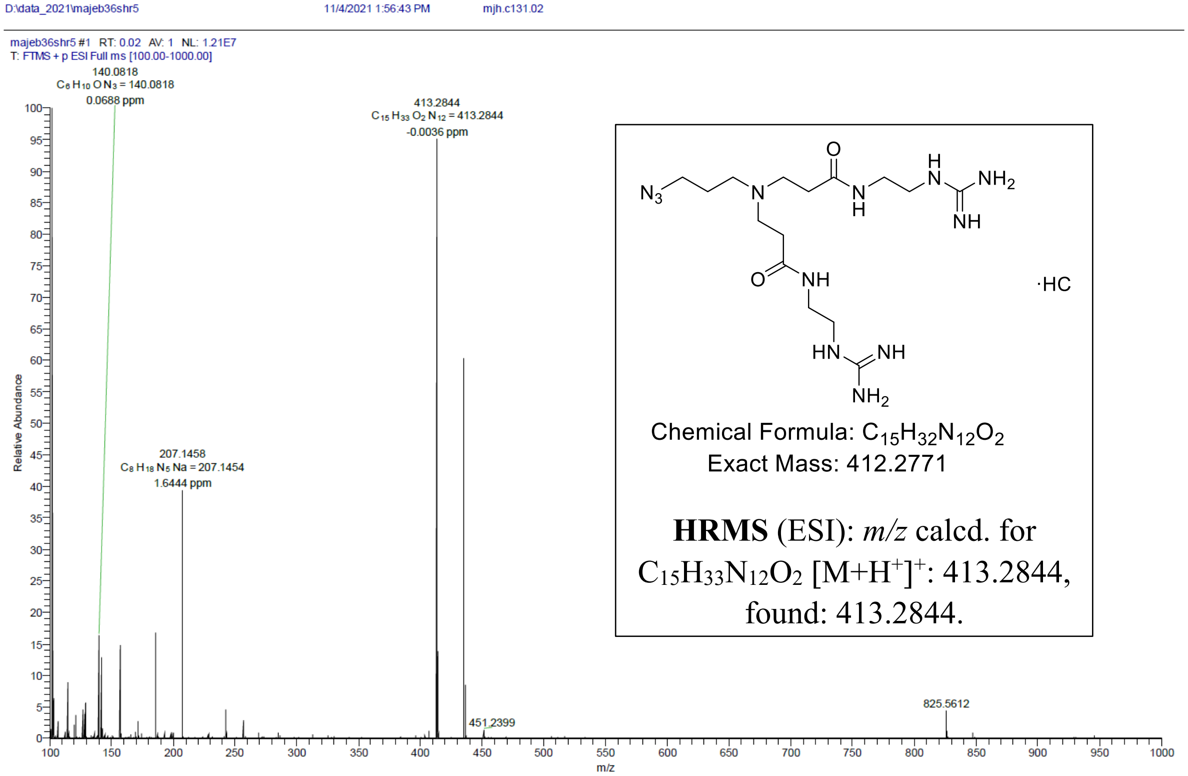

Figure 8.47. MASS spectrum of **12**.

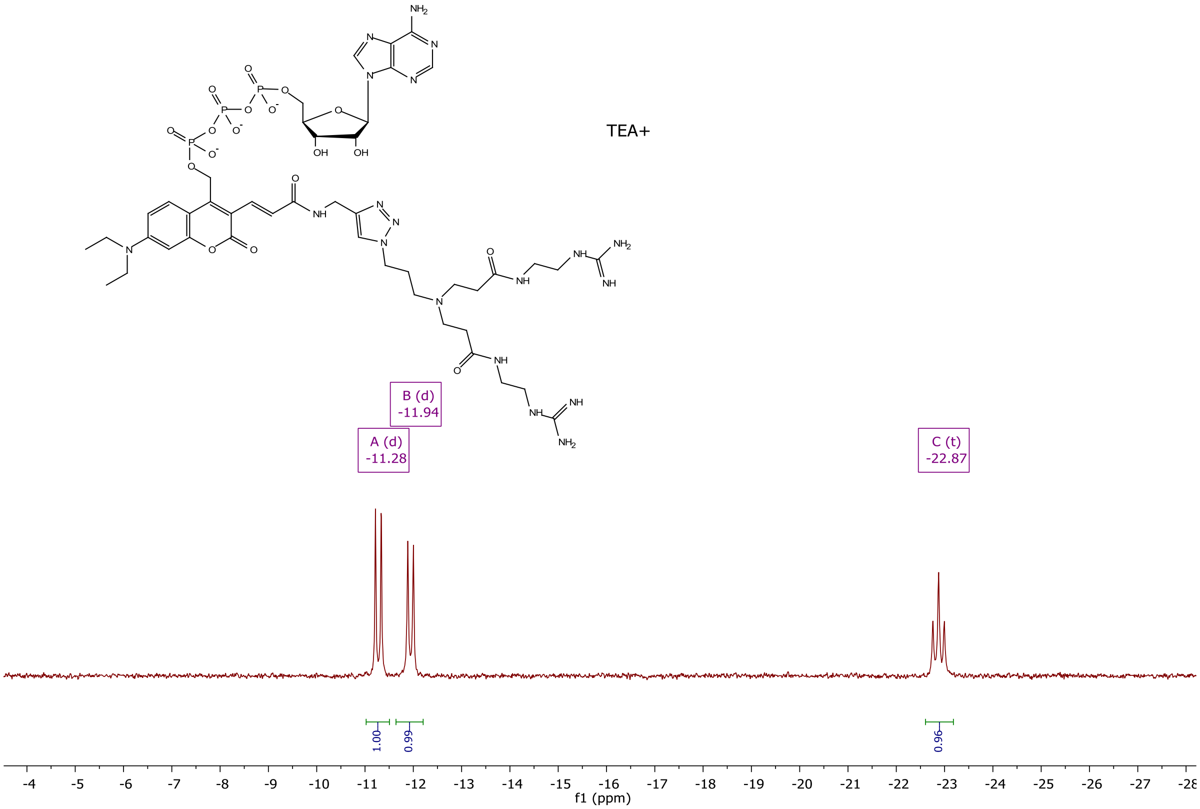

Figure 8.48. ^{31}**P** NMR spectrum of **20**.

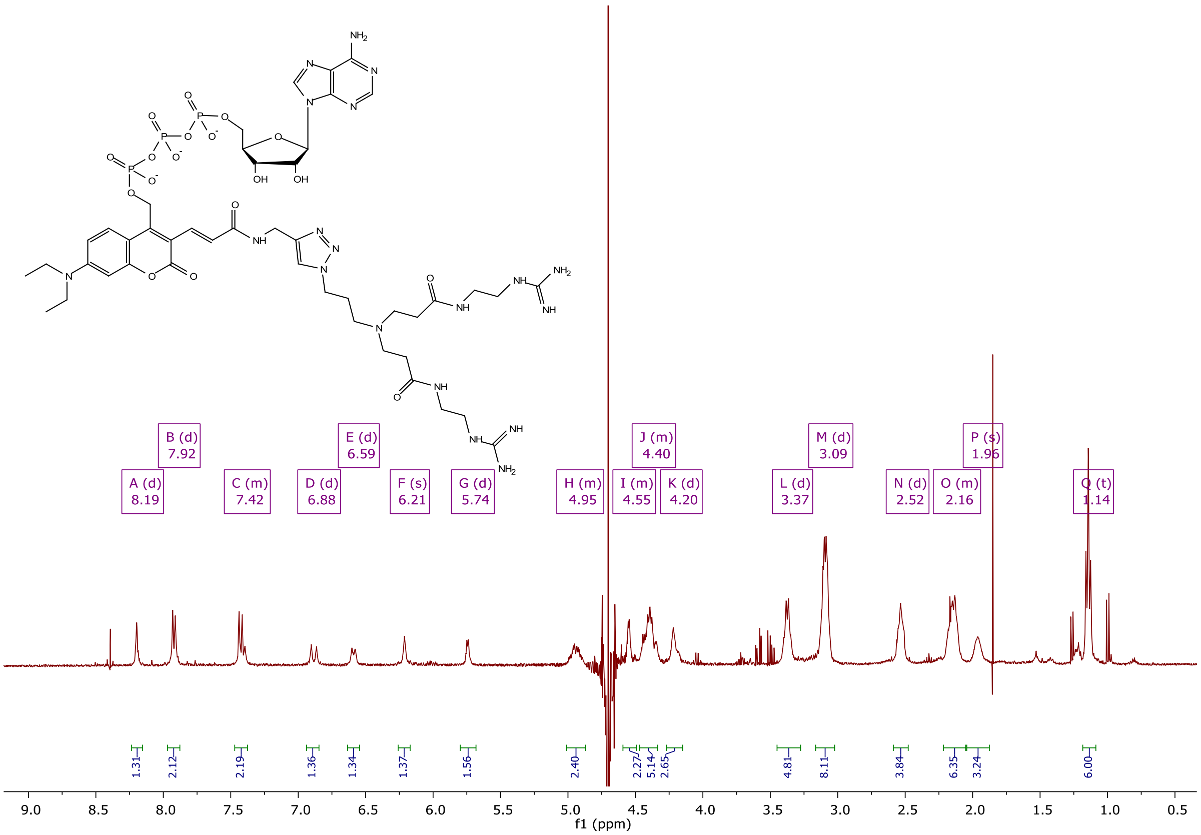

Figure 8.49. ^{1}**H** NMR spectrum of **20**.

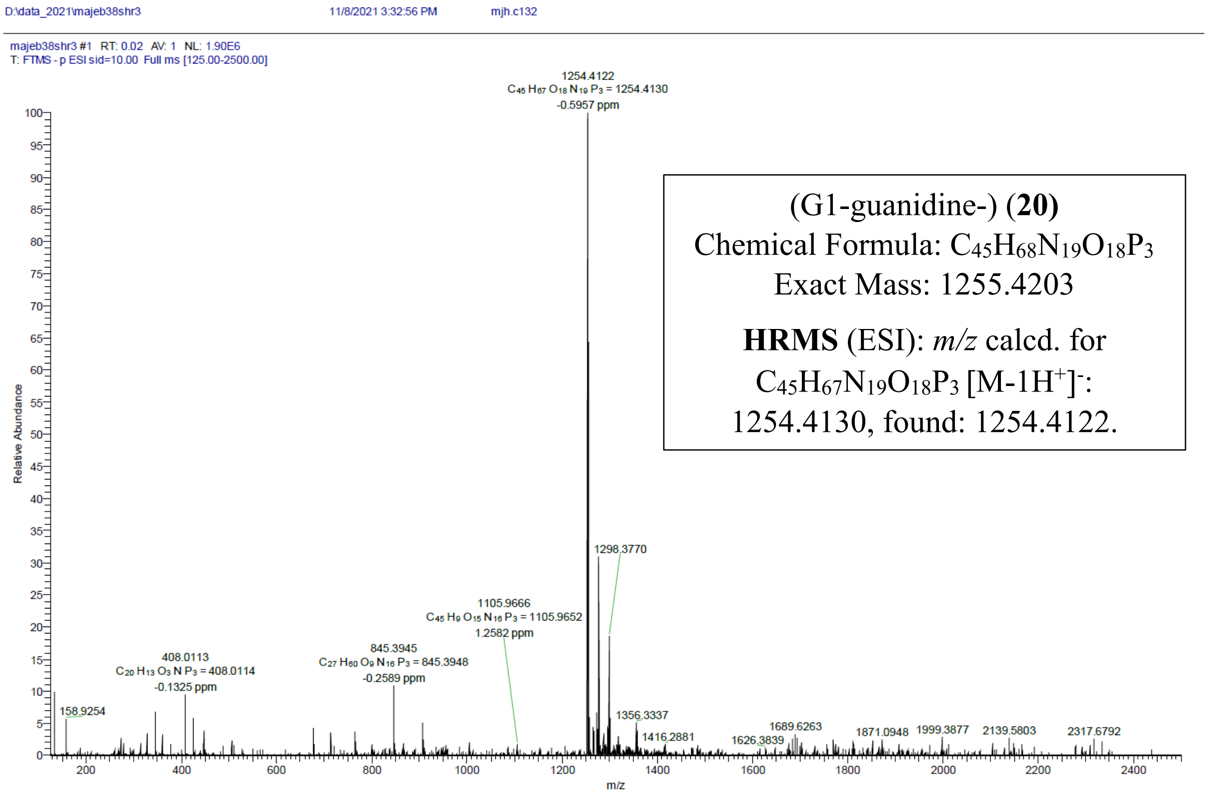

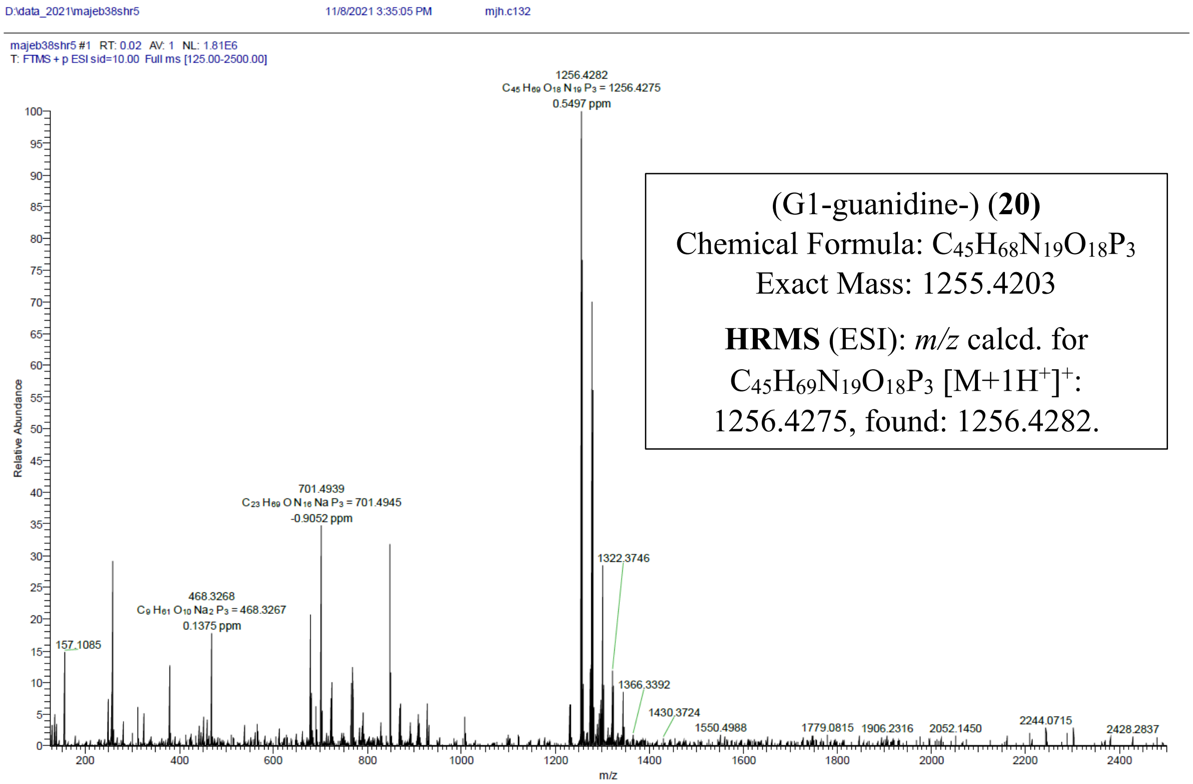

Figure 8.50. MASS spectra of **20**.

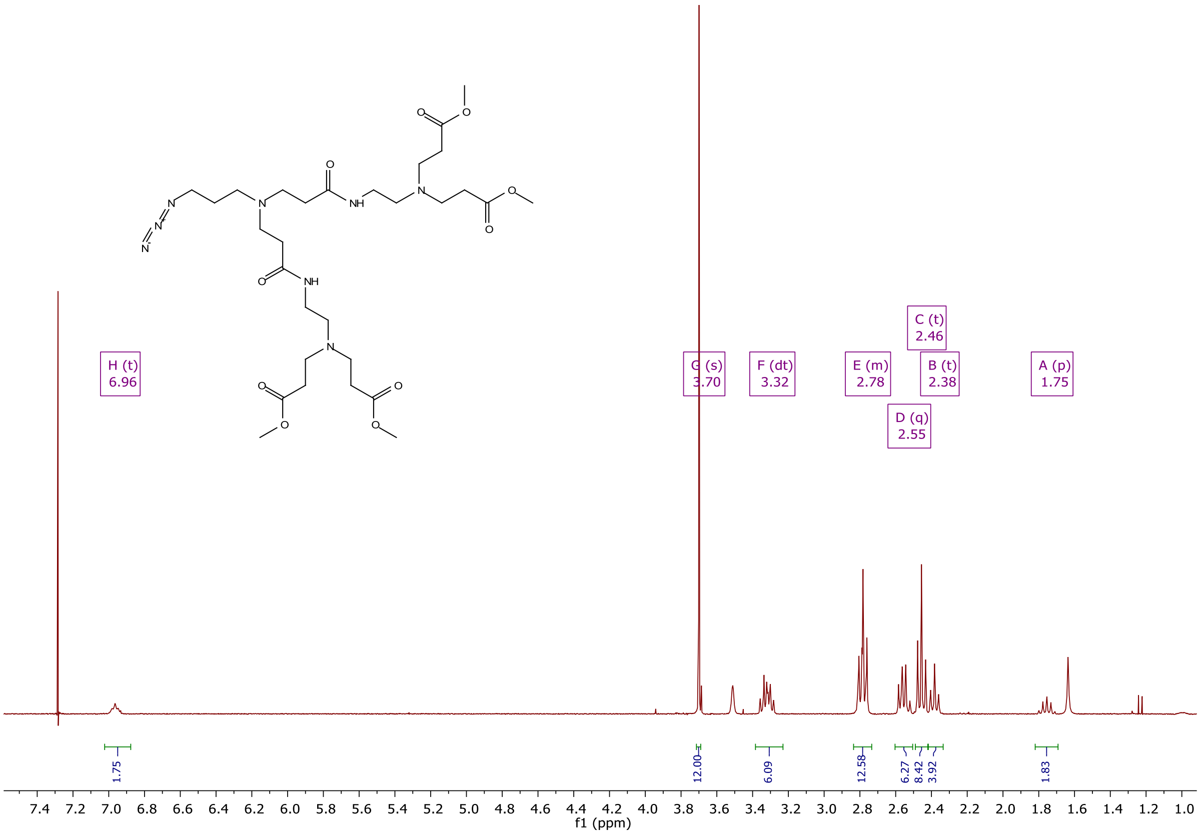

Figure 8.51. ^{1}**H** NMR spectrum of **G1.5 S10**.

Sample Spectra

+ Scan (rt: 0.090 min) Sub

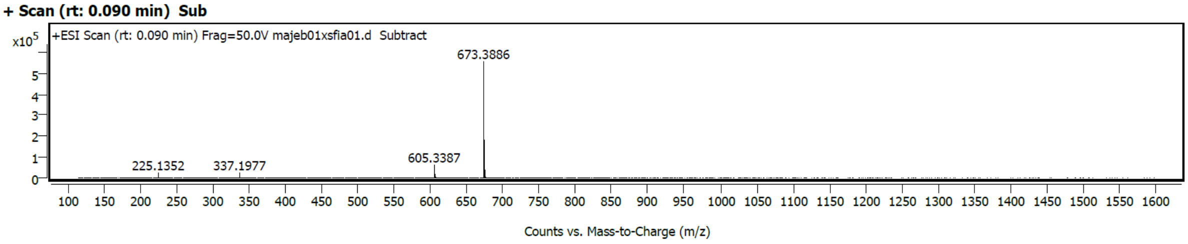

Spectrum Peaks

m/z	Z	Abund	Abund %	m/z (Calc)	Diff (ppm)	Ion Species	Formula	Ion Type
337.1977	2	25316	4.54	337.1976	0.20	(M+2H)+2	C29 H52 N8 O10	
337.6994	2	9668	1.73	337.6990	1.15	(M+2H)+2	C29 H52 N8 O10	
605.3387	1	62040	11.12	605.3379	1.42	(M+H)+	C25 H46 N7 O10	
606.3416	1	17683	3.17	606.3408	1.33	(M+H)+	C25 H46 N7 O10	
673.3886	1	557908	100.00	673.3879	1.04	(M+H)+	C29 H52 N8 O10	
674.3913	1	182126	32.64	674.3908	0.67	(M+H)+	C29 H52 N8 O10	
675.3928	1	38646	6.93	675.3933	-0.69	(M+H)+	C29 H52 N8 O10	
676.3964	1	6659	1.19	676.3958	0.97	(M+H)+	C29 H52 N8 O10	
225.1352	3	24139	4.33					
225.4704	3	9080	1.63					

Spectrum Identification Table

Best	ID Source	Name	Formula	Species	m/z	Diff (ppm)	CAS	Score	Score (Lib)	Score (DB)	Score (MFG)	Lib/DB
No	MFG		C29 H52 N8 O10	(M+H)+	673.3886	0.87		98.42			98.42	
No	MFG		C25 H46 N7 O10	(M+H)+	605.3387	1.58		97.18			97.18	
No	MFG		C29 H52 N8 O10	(M+2H)+2	337.1977	0.80		96.19			96.19	

Figure 8.52. MASS spectrum of **G1.5 S10**.

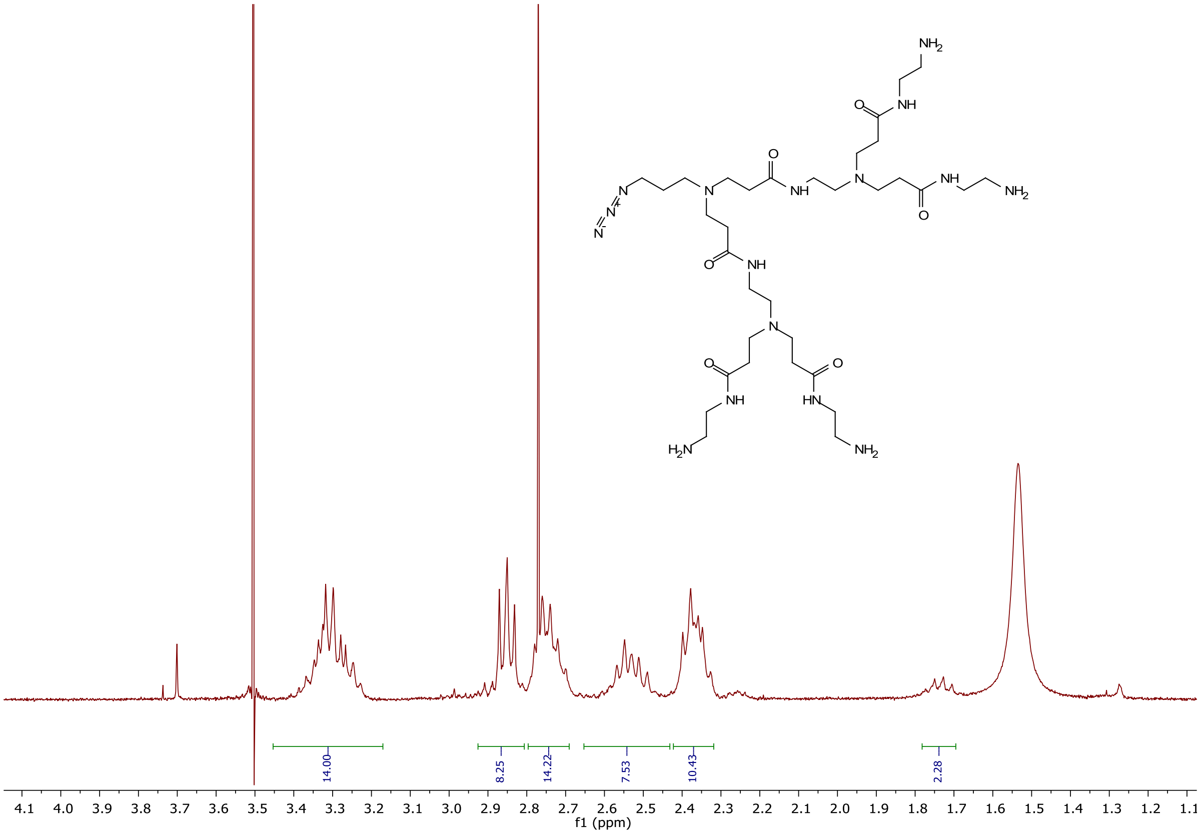

Figure 8.53. ^{1}H NMR spectrum of **13**.

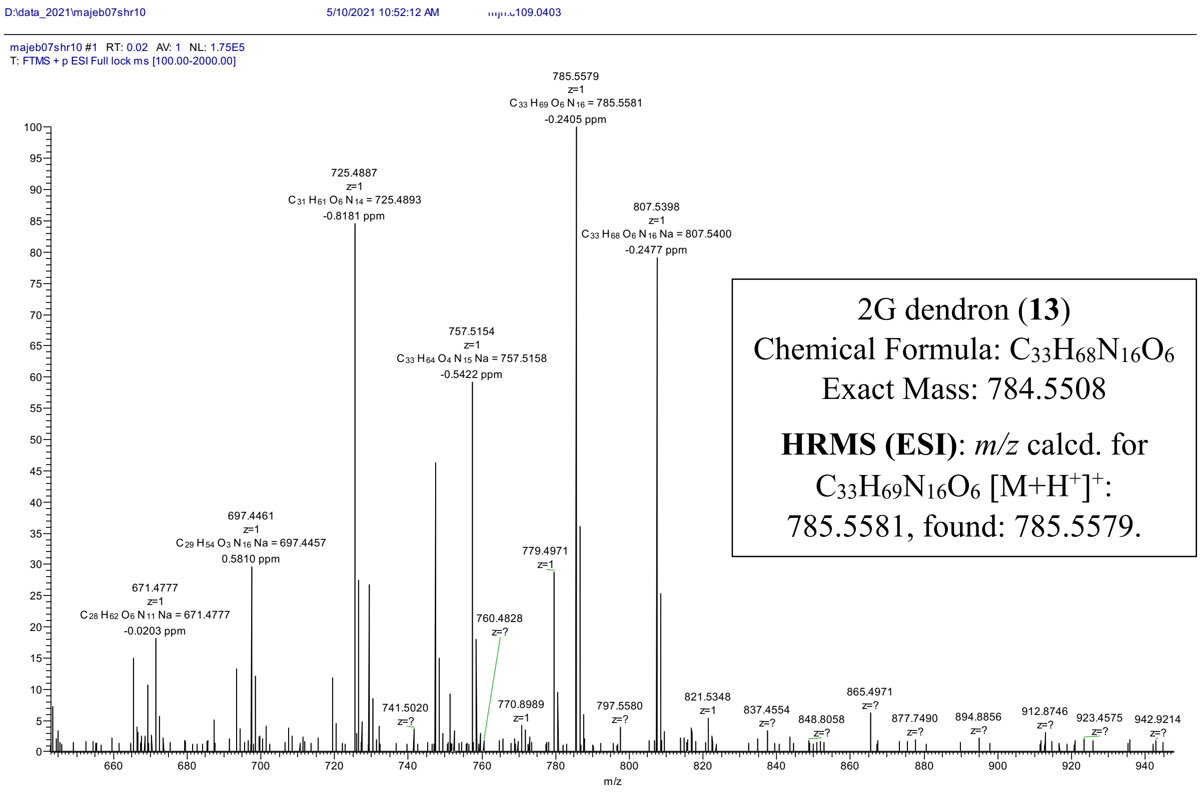

Figure 8.54. MASS spectrum of **13** (Reaction mixture after 10 days.).

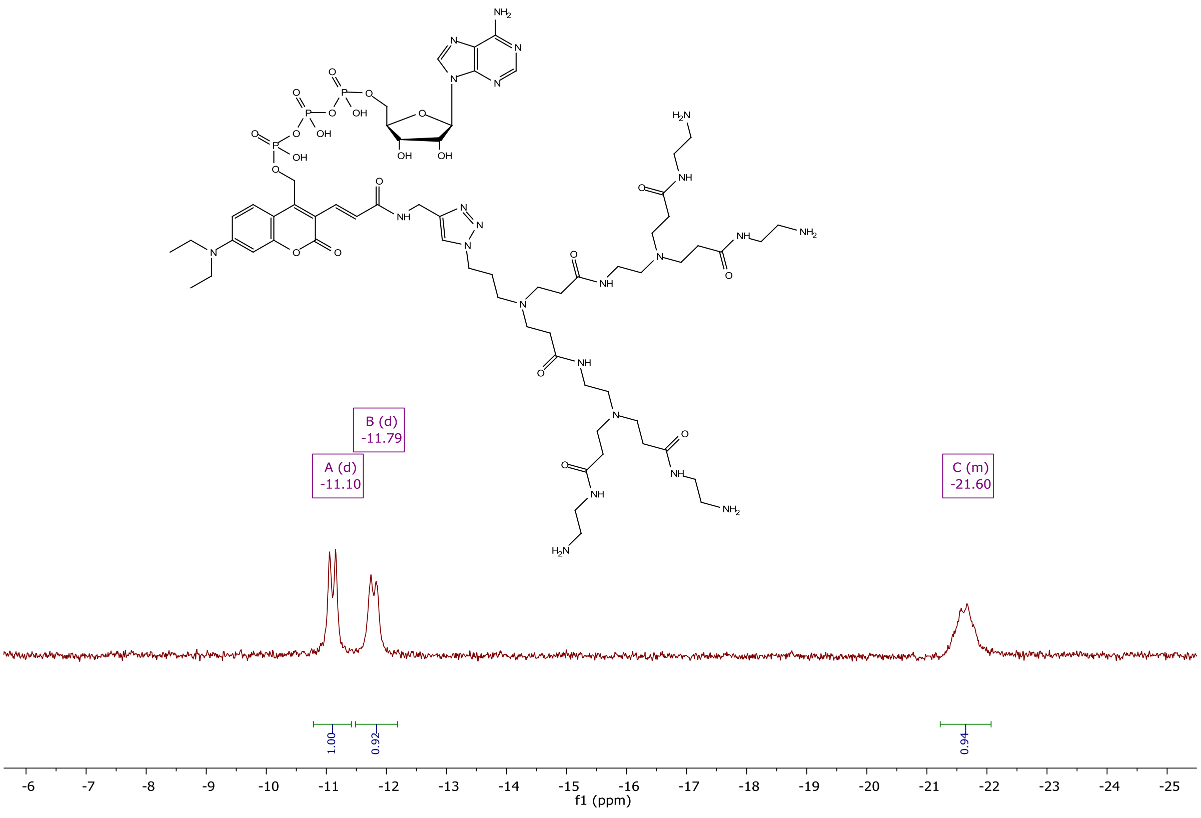

Figure 8.55. ^{31}P NMR spectrum of **21**.

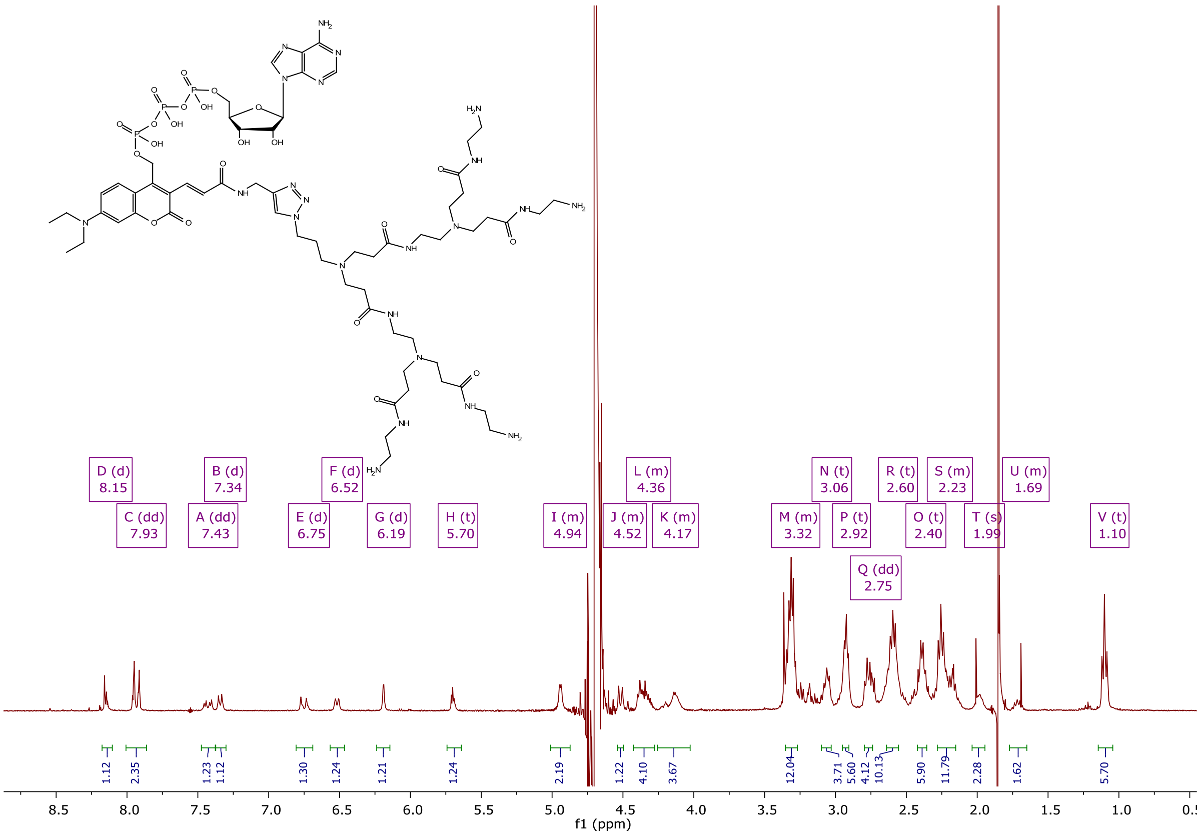

Figure 8.56. **^{1}H** NMR spectrum of **21**.

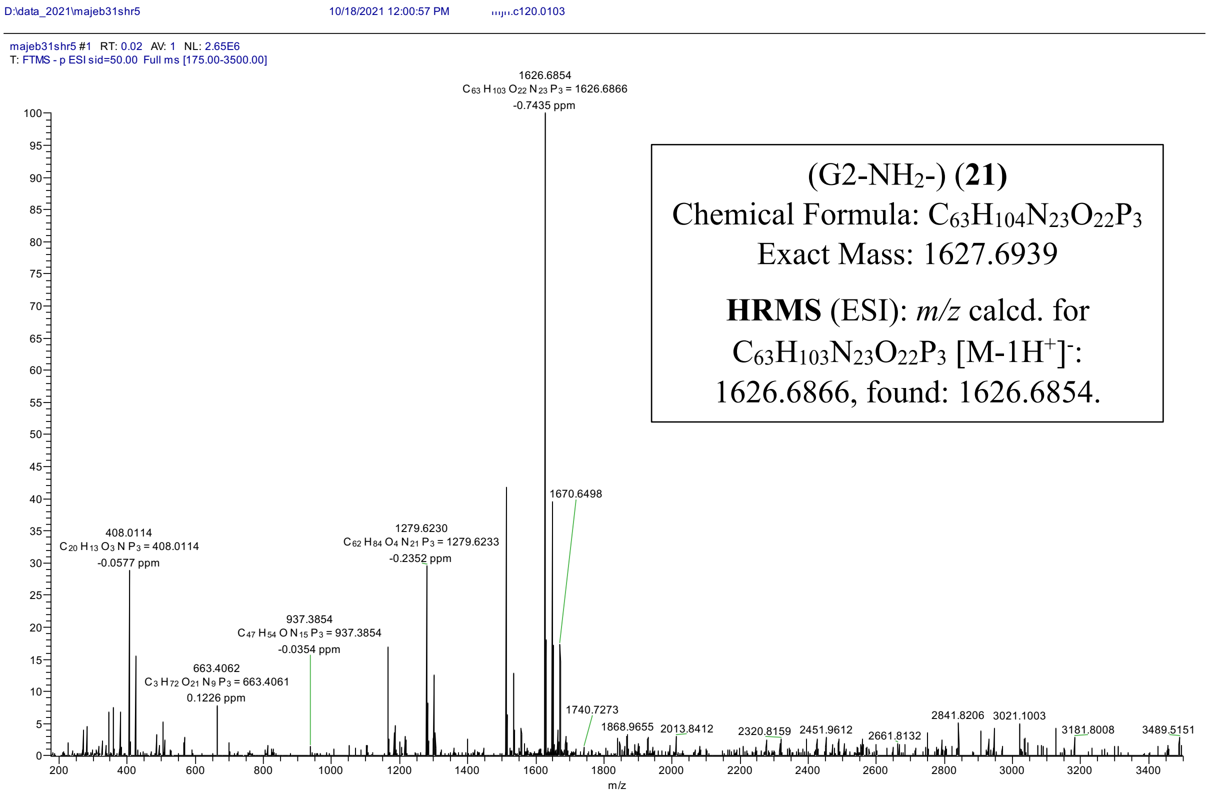

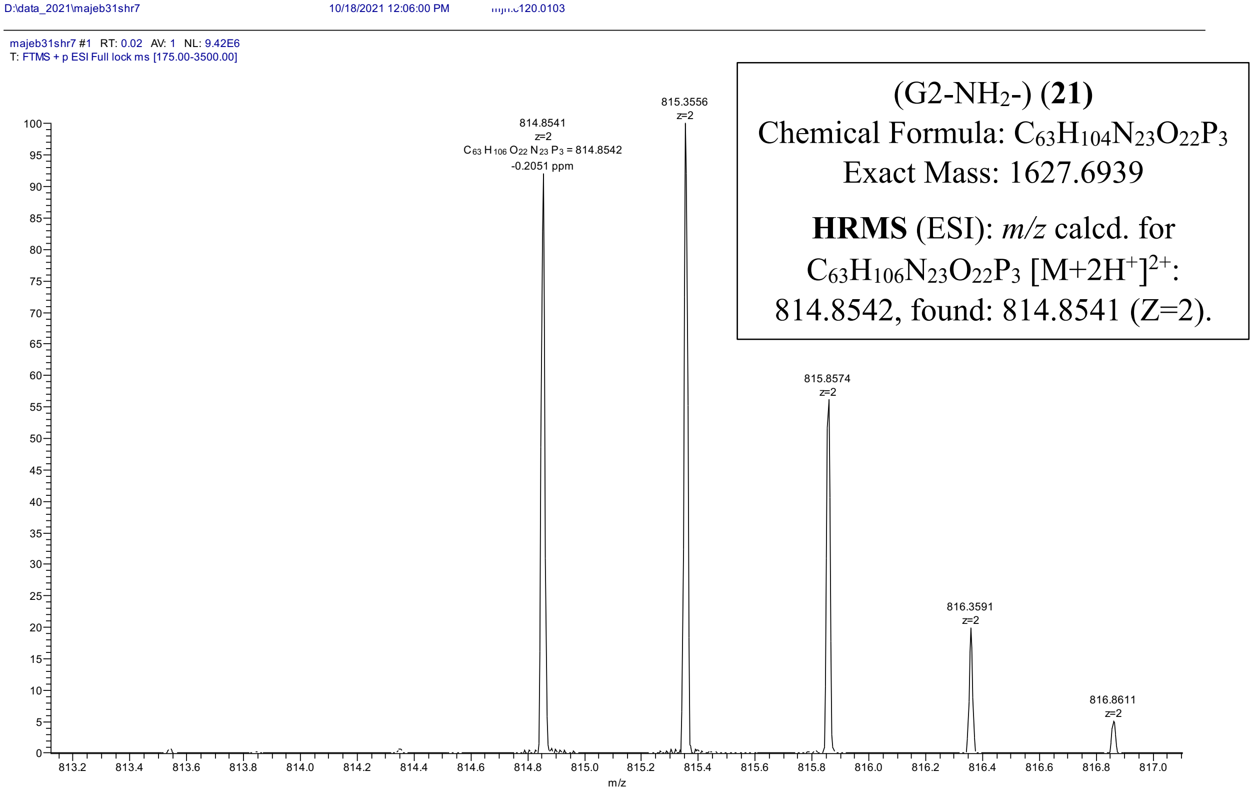

Figure 8.57. MASS spectra of **21**.

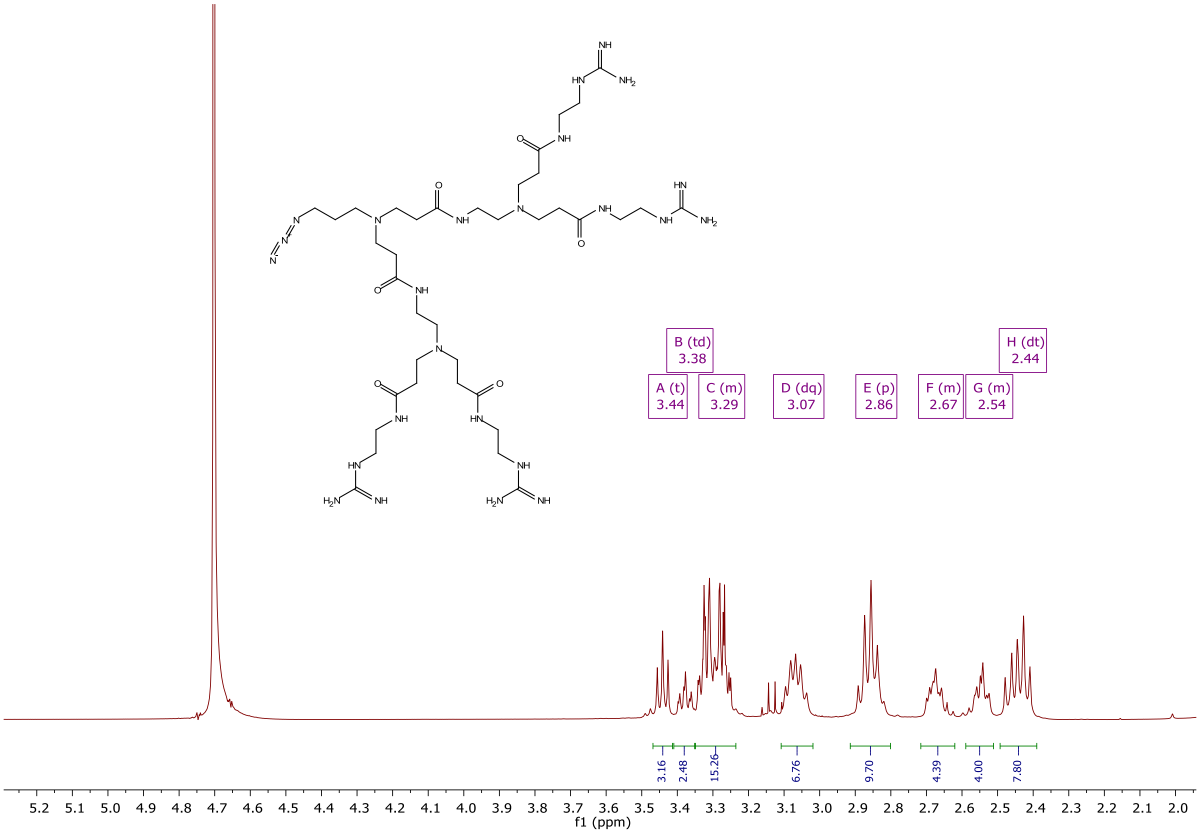

Figure 8.58. ^{1}H NMR spectrum of **14**.

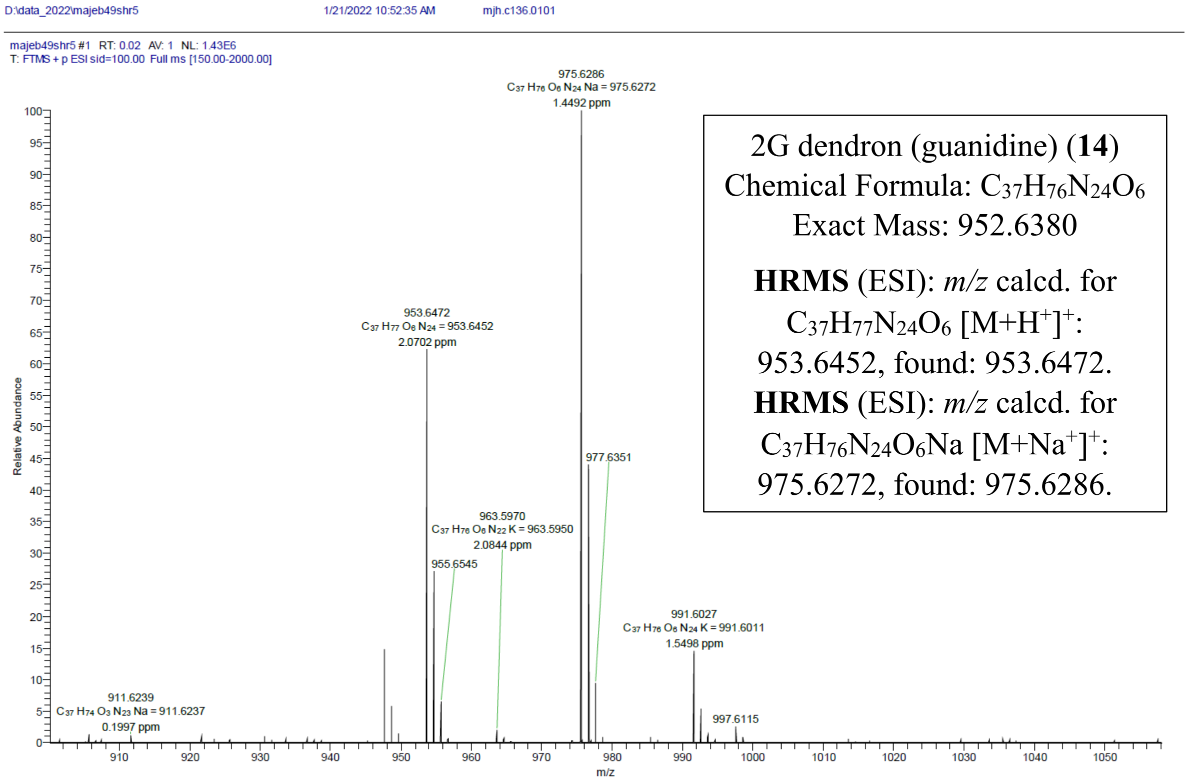

Figure 8.59. MASS spectrum of **14**.

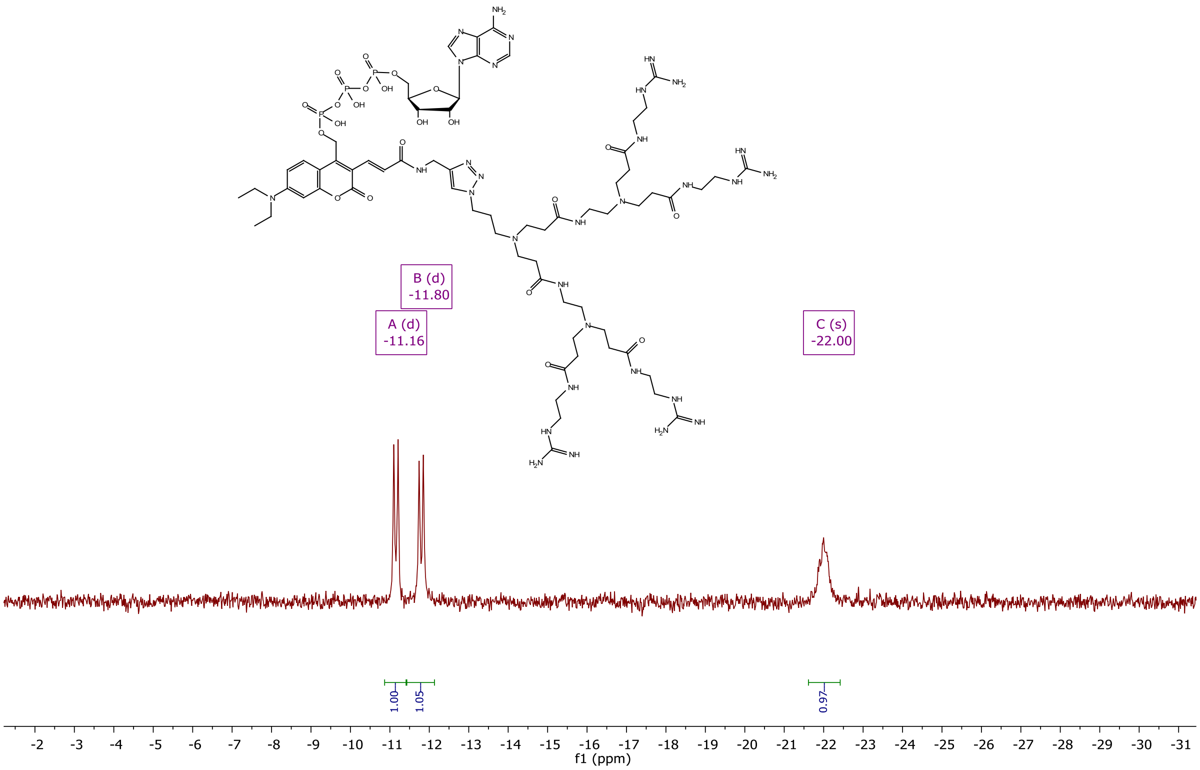

Figure 8.60. ^{31}**P** NMR spectrum of **22**.

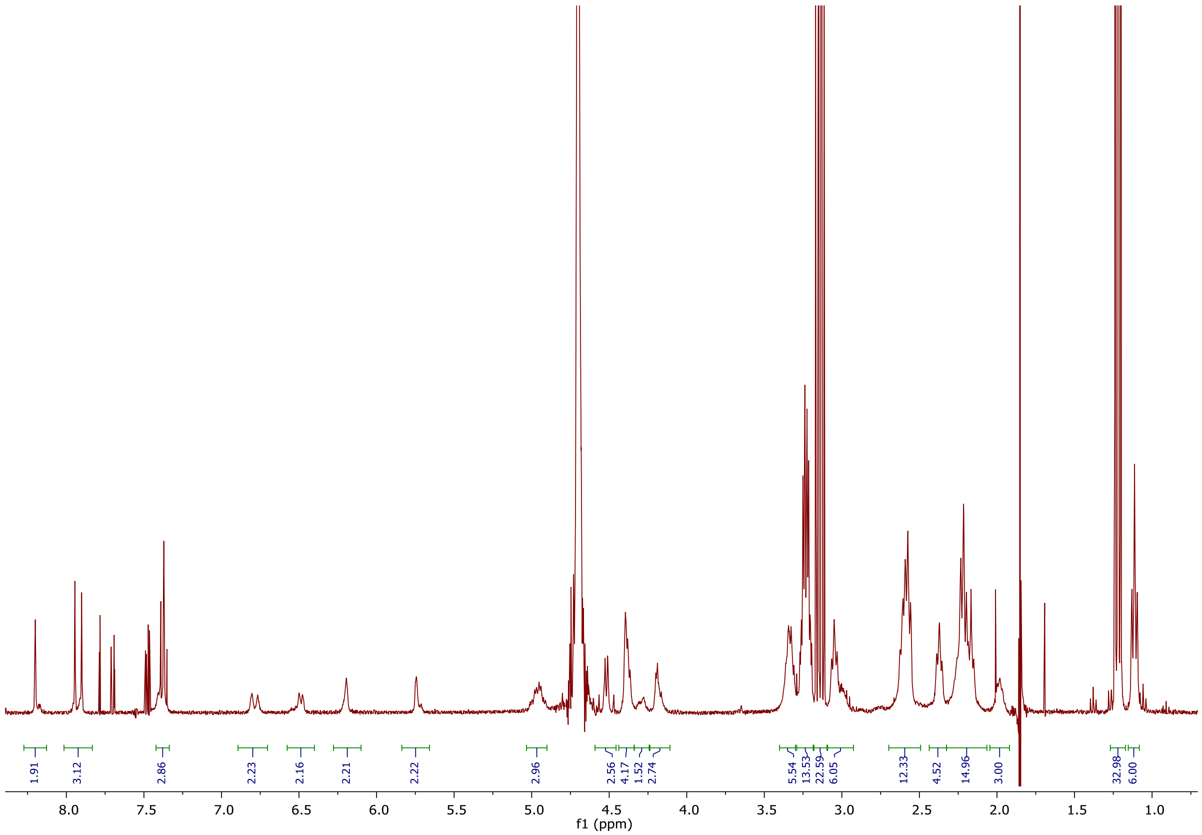

Figure 8.61. **^{1}H** NMR spectrum of **22.**

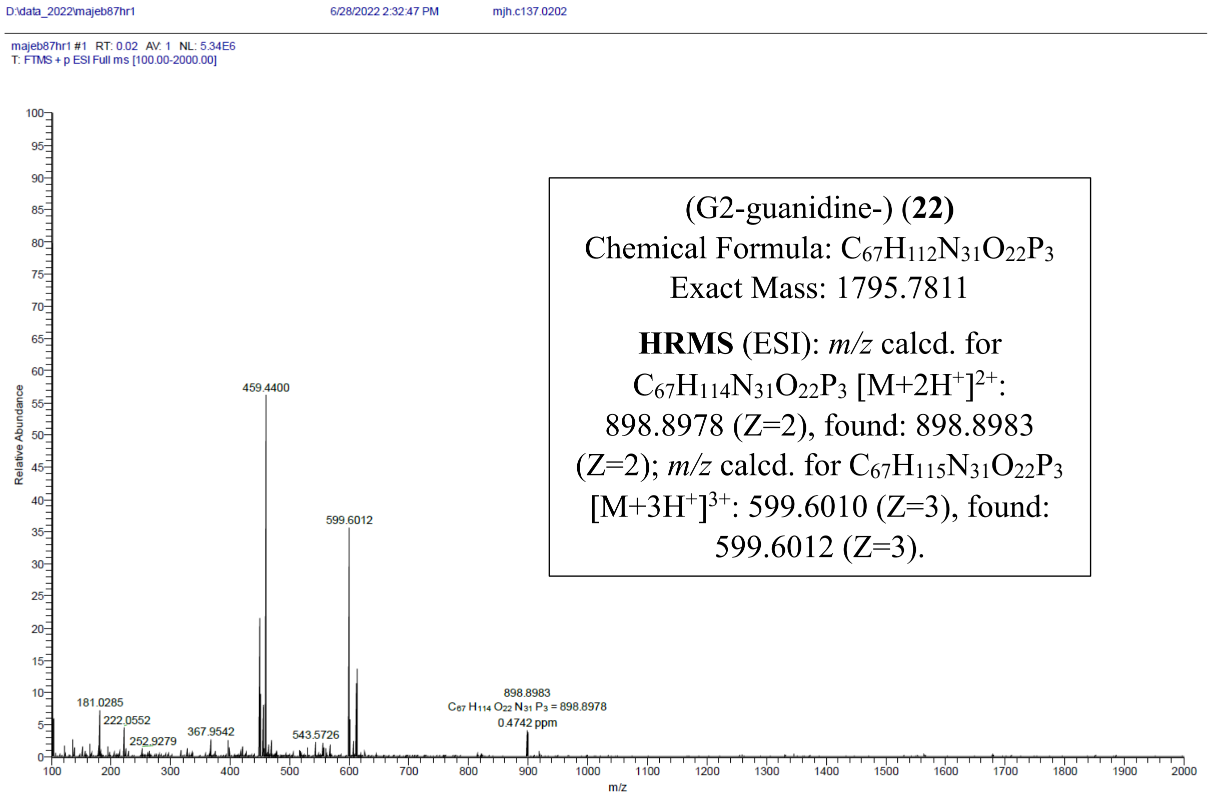

Figure 8.62. MASS spectrum of **22.**
